# Comparative analyses of the organelle genomes in *Jacaratia* *spinosa* (Caricaceae)

**DOI:** 10.3389/fpls.2025.1710417

**Published:** 2026-01-12

**Authors:** Liping Zuo, Zhicong Lin, Huiru Chen, Jianling Pan, Sihui Zhu, Ray Ming

**Affiliations:** 1 Center for Genomics and Biotechnology, Fujian Provincial Key Laboratory of Haixia Applied Plant Systems Biology, Fujian Agriculture and Forestry University, Fuzhou, China; 2 Key Laboratory of Genetics, Breeding and Multiple Utilization of Crops, Ministry of Education, Fujian Agriculture and Forestry University, Fuzhou, China; 3 College of Environmental and Biological Engineering, Putian University, Putian, China; 4 Fujian Provincial Key Laboratory of Ecology-Toxicological Effects & Control for Emerging Contaminants, Key Laboratory of Ecological Environment and Information Atlas (Putian University) Fujian Provincial University, Putian, China

**Keywords:** chloroplast genome, comparative analysis, gene transfer, *Jacaratia spinosa*, mitochondrial genome, phylogenetic analysis, repeat sequences, RNA editing events

## Abstract

**Introduction:**

*Jacaratia spinosa* (Aubl.) A. DC. (*J. spinosa*) is an important member of the Caricaceae family, valued for its edible properties and potential for protease development. However, organelle genome resources for this species have not been publicly available.

**Methods:**

To fill this gap, we applied a hybrid sequencing approach combining Illumina short reads and Nanopore long reads, and assembled the complete mitochondrial and chloroplast genomes of *J. spinosa* using established assembly pipelines, followed by comprehensive annotation and genomic feature analysis.

**Results:**

The circular mitochondrial genome spans 461,675 bp, and encodes 40 protein-coding genes (PCGs), 26 tRNA genes, and 3 rRNA genes. The complete chloroplast genome is 160,000 bp in length, comprising 84 PCGs, 37 tRNA genes, and 8 rRNA genes. Both genomes contain numerous repetitive sequences. Codon usage analysis revealed a preference for leucine and codons ending with A or U, and several non-canonical start and stop codons were corrected via RNA editing. We identified 34 homologous sequence fragments, indicating frequent intracellular gene transfer events between the mitochondrion and chloroplast. Phylogenetic analysis confirmed that *J. spinosa* is most closely related to *C. papaya* among the species included, forming a sister group. Synteny analysis revealed that while the chloroplast genome of *J. spinosa* is highly conserved, the mitochondrial genome exhibited high similarity but with notable structural rearrangements. Selection pressure analysis indicated that the mitochondrial genes *ccmFN* and *rps19*, as well as the chloroplast genes *ycf2* and *rps4*, are under positive selection.

**Discussion:**

These findings expand the organelle genome resources for Caricaceae and provide valuable molecular evidence for phylogenetic and evolutionary studies within the family.

## 1 Introduction

*Jacaratia* *spinosa* (Aubl.) A. DC. is an important member of the Caricaceae family, widely distributed throughout the Neotropical region from Guatemala to northeastern Argentina (Carvalho and Renner, 2012). It is a tall deciduous tree, reaching up to 15 meters in height, characterized by a narrow crown, short and stout conical spines on the trunk, and large, dark green, palmately lobed leaves with prominent palmate venation. The trunk is typically angular with distinct ridges, which is a diagnostic feature of the species. *J.* *spinosa* bears relatively small, elongated fruits that are rich in nutrients, making them edible for humans and an important food source for various Neotropical animals (Salas-Solano and Villalobos-Chaves, 2021). In Argentina, the stem is often used as a culinary ingredient due to its high water content and low lignin level (approximately 10%). Like other members of Caricaceae, which are known for producing abundant latex with proteolytic activity—particularly in the genera *Carica* and *Vasconcellea* (Scheldeman et al., 2007; Tigist et al., 2016)—the fruits of *J.* *spinosa* also exude copious latex. This enzyme-rich latex highlights the species’ potential biotechnological value as a natural source of proteolytic compounds.

The mitochondrial and chloroplast genomes in plants are two essential intracellular genetic systems that play central roles in energy metabolism and organismal development (Wang et al., 2024). Both organelles originated from ancient endosymbiotic events, with mitochondria derived from an ancestral *α*-proteobacterium and chloroplasts from a cyanobacterial symbiont (Dyall et al., 2004; Gray, 1992). Although most original genes have been lost during long-term co-evolution with the host, these organelles have retained a core set of genes essential for their basic functions (Timmis et al., 2004). Mitochondria generate ATP through oxidative phosphorylation and are involved in respiration, metabolite synthesis, programmed cell death, and signal transduction (Millar et al., 2011). While the number of protein-coding genes in plant mitochondria remains relatively conserved, mitochondrial genomes vary widely in size (200–700 Kb) and structure, often appearing as circular, multi-branched, or linear forms. They are typically rich in repetitive sequences and foreign DNA fragments, leading to frequent recombination and dynamic structural evolution (Gualberto and Newton, 2017). The largest known mitochondrial genome in angiosperms is that of *Silene conica*, which spans approximately 11.3 Mb and comprises 128 circular molecules (Sloan et al., 2012). In gymnosperms, *C. argyrophylla* possesses the largest reported mitochondrial genome to date, with a total size of 18.99 Mb (Huang et al., 2025). Moreover, the structural complexity of plant mitochondrial genomes is closely associated with important agronomic traits such as cytoplasmic male sterility (CMS) (Hanson and Bentolila, 2004).

Chloroplasts are the primary sites of photosynthesis in plants and also play vital roles in the metabolism of fatty acids and pigments (Neuhaus and Emes, 2000). The chloroplast genome is typically a double-stranded circular DNA molecule ranging from 120 to 170 kb in size, comprising a large single-copy region (LSC), a small single-copy region (SSC), and a pair of inverted repeats (IRs) (Palmer, 1985). Although the overall structure is relatively conserved, significant variations exist among species in gene order, IR expansion, and intergenic regions (Jansen and Ruhlman, 2012), providing valuable information for phylogenetic studies. Due to its maternal inheritance and moderate evolutionary rate, the chloroplast genome offers high resolution in distinguishing closely related species and has been widely used in plant evolutionary research (Vydianathan et al., 2007).

The family Caricaceae comprises approximately six genera and 40 species. Currently, only the mitochondrial genome of *C. papaya* and the chloroplast genomes of three species (*Carica papaya, Vasconcellea cundinamarcensis*, and *Vasconcellea carvalhoae*) have been reported (Rice et al., 2008; Tineo et al., 2022; Lin et al., 2020). Although *J.* *spinosa* is closely related to *C. papaya*, its phylogenetic position remains uncertain due to the lack of complete organelle genome data (Carvalho and Renner, 2012). In this study, we combined Illumina and Nanopore sequencing technologies to assemble and annotate the complete mitochondrial and chloroplast genomes of *J.* *spinosa*. We systematically analyzed features such as codon usage bias, RNA editing sites, and gene transfer events. Phylogenetic and comparative genomic analyses further clarified the evolutionary position of *J.* *spinosa* within Caricaceae. Our findings enrich the genomic resources of this family and provide valuable insights for future evolutionary studies.

## 2 Materials and methods

### 2.1 Plant materials

Fresh leaves of *J.* *spinosa* were collected from the Germplasm Greenhouse of Fujian Agriculture and Forestry University, Fuzhou, Fujian Province, China (geographic coordinates: 26°04′54″N, 119°13′51″E) (**Supplementary Figure S1A**). After collection, the leaves were rinsed with PBS buffer, air-dried at room temperature, and immediately frozen in liquid nitrogen, then stored at -80 °C until further use. Genomic DNA was extracted using the CTAB method (Doyle and Doyle, 1987). Total RNA was isolated using the MiniBEST Universal RNA Extraction Kit (Cat. No. 9767; Takara, Japan) according to the manufacturer’s instructions. The quality of the extracted DNA and RNA was assessed using a NanoDrop spectrophotometer (Thermo Fisher Scientific, USA), a Qubit fluorometer (Thermo Fisher Scientific, USA), and 1% agarose gel electrophoresis to evaluate the purity, concentration, and integrity of the nucleic acids.

### 2.2 Genome sequencing, assembly, and annotation

Paired-end short-read sequencing (2 × 150 bp) was conducted on the Illumina platform for both genome DNA and transcriptome RNA. The DNA library was used for genome assembly (hereafter referred to as DNA-Illumina), and the RNA library was used for RNA editing site detection (hereafter referred to as RNA-Illumina). The raw DNA-Illumina data (70 GB) were quality controlled using fastp (v0.23.4) (Chen, 2023) to remove low-quality reads, adapter sequences, and reads containing excessive ambiguous bases (N), yielding 68 GB of clean data. The same procedure was applied to the raw RNA-Illumina data (3.73 GB), resulting in 3.51 GB of clean data. Long-read sequencing for genome assembly was performed on the Oxford Nanopore PromethION platform, generating approximately 12.27 Gb of long reads. Reads shorter than 1 kb and the bottom 10% in quality score were filtered using Filtlong (v0.3.1) (Wick et al., 2023), and the quality of the filtered reads was assessed with NanoPlot (v1.46.1) (De Coster and Rademakers, 2023), resulting in 11.04 Gb of clean data.

Organelle genomes were assembled using two complementary methods for cross-validation. The mitochondrial genome was initially assembled from ONT long reads by NECAT (v0.01) (Chen et al., 2021) and polished with Illumina short reads using NextPolish (Hu et al., 2020). The mitochondrial contig of *J.* *spinosa* was identified by alignment to the reference genome of *C. papaya* (GenBank: EU431223.1). The chloroplast genome was assembled from Illumina paired-end data using GetOrganelle (v1.7.7.0) (Jin et al., 2020). To validate, PMAT (v1.5.3) (Bi et al., 2024) reassembled both organelle genomes from ONT reads with autoMito parameters and graphBuild. Sequences supported by both methods were selected as candidates. Assembly graphs were visualized with Bandage (v0.8.1) (Wick et al., 2015), and redundant repeats were removed based on sequencing depth, yielding finalized complete organelle genomes.

Mitochondrial and chloroplast genome annotations were performed using GeSeq (https://chlorobox.mpimp-golm.mpg.de/geseq.html) (Tillich et al., 2017). The annotation results were manually curated with Apollo (Lee et al., 2013). Organelle genome maps were generated using OGDRAW (v1.3.1) (https://chlorobox.mpimp-golm.mpg.de/OGDraw.html) (Greiner et al., 2019).

### 2.3 Identification of repetitive sequences and selection pressure analysis

Simple sequence repeats (SSRs) were identified using the online tool MISA (https://webblast.ipk-gatersleben.de/misa/) (Thiel et al., 2003) with the following parameters in misa.ini: 1-10, 2-5, 3-4, 4–3, 5-3, 6-3. Tandem repeats were detected using Tandem Repeats Finder (TRF, https://tandem.bu.edu/trf/) (Benson, 1999) with parameters set to: 2 7 7 80 10 50 500 -f -d -m. Dispersed repeats were identified using the online tool REPuter (https://bibiserv.cebitec.uni-bielefeld.de/reputer/) (Kurtz et al., 2001), with a minimum repeat length of 30 bp.

To assess selective pressures acting on PCGs, KaKs_Calculator (Wang et al., 2010) was used to estimate the rates of nonsynonymous (Ka) and synonymous (Ks) substitutions and to compute their ratio (Ka/Ks). A Ka/Ks value less than 1 indicates purifying selection, a value near 1 suggests neutral evolution, and a value greater than 1 implies possible positive selection.

### 2.4 Codon usage bias analysis and identification of IGT events

PCG sequences were extracted from the organelle genomes using PhyloSuite (v1.1.12) (Zhang et al., 2020). Relative synonymous codon usage (RSCU) values were calculated with MEGA (v7.0.26) (Kumar et al., 2016), where RSCU = 1 indicates no bias, >1 indicates preference, and <1 indicates low usage.

Homologous segments between mitochondrial and chloroplast genomes were identified by BlastN (v2.14.0) (Altschul et al., 1990) with an E-value cutoff of 1 × 10^^−5^. Their chromosomal distribution was visualized using the Advanced Circos plugin in TBtools (Chen et al., 2020).

### 2.5 RNA editing site analysis

The organelle genomes were indexed using HISAT2 (v2.1.0) (Kim et al., 2019), and RNA-seq reads were aligned to these genomes. SAM files were converted to BAM format with samtools (v1.15.1) (Li et al., 2009), and variants were called using bcftools (v1.19) (Danecek et al., 2021). Sites with quality scores ≥20 and supported by at least 20 reads were retained, followed by manual filtering of false positives in IGV (Robinson et al., 2011). To confirm the authenticity of RNA editing sites, primers were designed for PCR amplification, and Sanger sequencing was performed.

### 2.6 Phylogenetic and synteny analysis

Chloroplast genome annotation files of 11 closely related species were retrieved from NCBI (**Table S12**). A set of 69 shared single-copy orthologous genes was extracted using PhyloSuite (Zhang et al., 2020), and aligned with MAFFT (v7.450) (Katoh and Standley, 2013) under default parameters. A maximum likelihood (ML) phylogenetic tree was inferred using IQ-TREE (v1.6.8) (Nguyen et al., 2014), with the “GTR+F+I+G4” substitution model selected as the best-fit model according to the Bayesian Information Criterion (BIC). The resulting phylogenetic tree was visualized and annotated using Interactive Tree of Life (iTOL) (https://itol.embl.de) (Letunic and Bork, 2021), allowing for clear presentation of species relationships and clade support values.

Synteny between mitochondrial and chloroplast genomes of Caricaceae species was analyzed using Mauve (v2.3.1) (Darling et al., 2004) with an LCB (Locally Collinear Block) weight of 1068. Structural variation in chloroplast genomes was further examined using the mVISTA (Frazer et al., 2004) online tool.

## 3 Results

### 3.1 Mitogenome and chloroplast assembly and genomic features of *J.* *spinosa*

**Figure 1:**
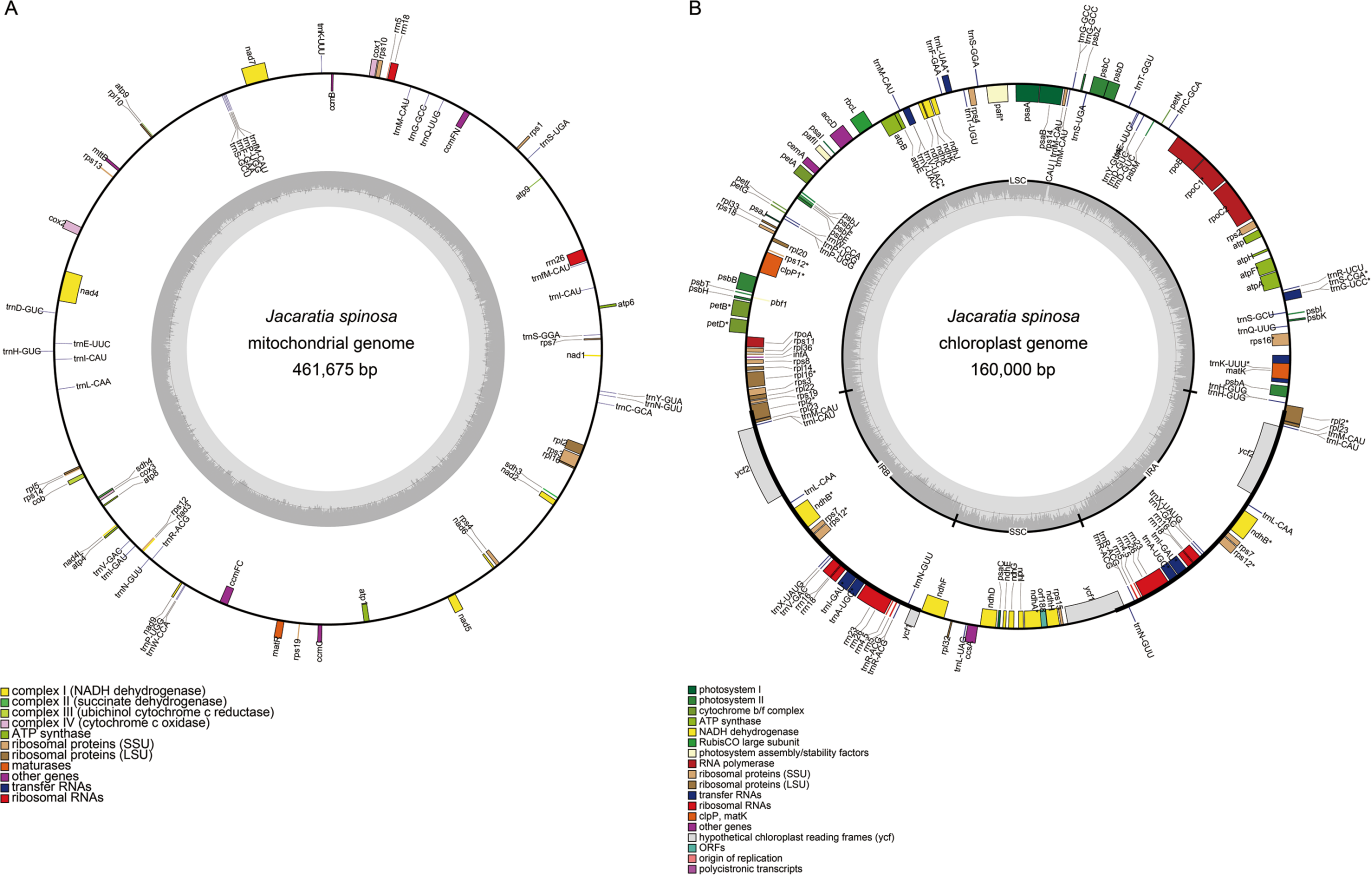
Assembly results of the *J.spinosa.***(A)** Mitogenome **(B)** Chloroplast. Genes with different functions are color-coded. Genes on the outside and inside of the circle are transcribed clockwise and counterclockwise, respectively. The innermost gray plot shows GC content, with the central gray line indicating the 50% threshold.

The mitochondrial genome of *J.* *spinosa* was assembled into a typical circular molecule, with a total length of 461,675 bp and a GC content of 44.79%. The base composition is as follows: adenine (A) 27.60%, thymine (T) 27.62%, cytosine (C) 22.54%, and guanine (G) 22.25%. (**Supplementary Figure S1B**, **Figure 1A**). Genome annotation revealed that protein-coding sequences account for 7.25% of the total genome, rRNA genes for 1.17%, and tRNA genes for 0.43%. In total, 69 functional genes were identified, including 40 protein-coding genes (PCGs)—comprising 16 core and 24 variable genes—along with 26 tRNA genes and 3 rRNA genes. Intron distribution analysis showed that *nad1*, *nad2*, *nad5*, *nad7* each contain four introns; *nad4* contains three introns; and *ccmFC*, *cox2*, *rpl2*, *rps10*, and *rps3* each contain one intron. Notably, the *atp9* gene is present in two copies in *J.* *spinosa*, and four tRNA genes—*trnfM-CAU*, *trnI-CAU*, *trnN-GUU*, and *trnP-UGG*—are also duplicated (**Table 1**).

**Table 1:** Gene composition in this mitochondrial genome of *J. spinosa*.

Category of genes	Group of genes	Name of genes
core genes	Subunit of ATPase	*atp1, atp4, atp6, atp8, atp9* (×2)
	Cytochrome c biogenesis	ccmB, ccmC, ccmFC*, ccmFN
	Apocytochrome b	*cob*
	Subunit of cytochrome c oxidase	cox1, cox2*, cox3
	Maturase R	*matR*
	Transport membrane protein	*mttB*
variable genes	Subunit of NADH dehydrogenase	nad1****, nad2****, nad3, nad4***, nad4L, nad5****, nad6, nad7****, nad9
	Small subunit of ribosome	rps1, rps10*, rps12, rps13, rps14, rps19, rps3*, rps4, rps7
	Large subunit of ribosome	rpl10, rpl16, rpl2*, rpl5
	Subunit of succinate dehydrogenase	*sdh3, sdh4*
tRNA genes	tRNA genes	*trnI-GAU,trnC-GCA,trnD-GUC,trnE-UUC,trnF-GAA,trnG-GCC,trnH-GUG,trnK-UUU,trnL-CAA,trnM-CAU,trnfM-CAU* (×2)*, rnI-CAU* (×2)*, trnN-GUU* (×2)*, trnP-UGG* (×2)*, trnQ-UUG,trnR-ACG,trnS-GCU,trnS-GGA,trnS-UGA,trnV-GAC,trnW-CCA,trnY-GUA*
rRNA genes	rRNA genes	*rrn18, rrn26, rrn5*

*intron number; Gene (×2): Number of copies of multi-copy genes. *** represents a gene with 3 introns; **** represents a gene with 4 introns.

The chloroplast genome of *J.* *spinosa* exhibited a typical quadripartite structure and was assembled into a circular molecule with a total length of 160,000 bp and a GC content of 36.83% (**Supplementary Figure S1C**, **Figure 1B**). It comprises a pair of inverted repeat (IR) regions of 25,588 bp (GC, 42.67%) each, a large single-copy (LSC) region of 90,197 bp (GC, 34.74%), and a small single-copy (SSC) region of 18,627 bp (GC, 30.90%). A total of 129 genes were annotated, including 84 protein-coding genes, 8 rRNA genes, and 37 tRNA genes. Intron distribution analysis showed that the *atpF, ndhA, ndhB, rps16, rpl2, rpoC1, petB, petD* and *rpl16* each contain one intron, while the *rps12, ycf3*, and *clpP* genes each contain two introns. Due to the presence of the inverted repeat (IR) regions, a total of 18 genes were duplicated (**Table 2**).

**Table 2:** Gene composition in this chloroplast genome of *J. spinosa*.

Category of genes	Group of genes	Name of genes
Genes for photosynthesis	Subunits of ATP synthase	atpA, atpB, atpE, atpF^*^, atpH, atpI
	Subunits of photosystem II	psbA, psbB, psbC, psbD, psbE, psbF, psbH,psbI, psbJ, psbK, psbL, psbM, psbN, psbT, psbZ, ycf3**
	Subunits of NADH-dehydrogenase	ndhA*, ndhB (×2)*, ndhC, ndhD, ndhE, ndhF, ndhG, ndhH, ndhI, ndhJ, ndhK
	Subunits of cytochrome b/f complex	petA, petB*, petD*, petG, petL, petN
	Subunits of photosystem I	*psaA, psaB, psaC, psaI, psaJ*
	Subunit of rubisco	*rbcL*
Self replication	Large subunit of ribosome	rpl14, rpl16*, rpl2 (×2)*, rpl20, rpl22, rpl23(×2)*, rpl32, rpl33, rpl36*
	DNA dependent RNA polymerase	rpoA, rpoB, rpoC1^*^, rpoC2
	Small subunit of ribosome	*rps11, rps12*(×2)**, rps14, rps15, rps16*, rps18, rps19, rps2, rps3, rps4, rps7(×2), rps8
	Ribosomal RNA genes (rRNA)	*rrn16S*(×2)*, rrn23S*(×2)*, rrn5S*(×2)*, rrn4.5S*(×2)
	Transfer RNA genes (tRNA)	*trnA-UGC*(×2)*, trnC-GCA, trnD-GUC, trnE-UUC, trnF-GAA, trnG-GCC, trnG-UCC, trnH-GUG, trnI-CAU*(×2)*, trnI-GAU*(×2)*, trnK-UUU, trnL-CAA*(×2)*, trnL-UAA, trnL-UAG, trnM-CAU*(×2)*, trnN-GUU*(×2*), trnP-UGG, trnQ-UUG, trnR-ACG*(×2)*, trnR-UCU, trnS-GCU, trnS-GGA, trnS-UGA, trnT-GGU, trnT-UGU, trnV-GAC*(×2)*, trnV-UAC, trnW-CCA, trnY-GUA*
Other genes	Subunit of Acetyl-CoA-arboxylase	*accD*
	c-type cytochrom synthesis gene	*ccsA*
	Envelop membrane protein	*cemA*
	Protease	clpP**
	Maturase	*matK*
Unknown	Conserved open reading frames	*ycf1, ycf2*(×2)*, ycf4*

*intron number; Gene (×2): Number of copies of multi-copy genes. ** represents a gene with 2 introns.

### 3.2 Repetitive sequences

**Figure 2:**
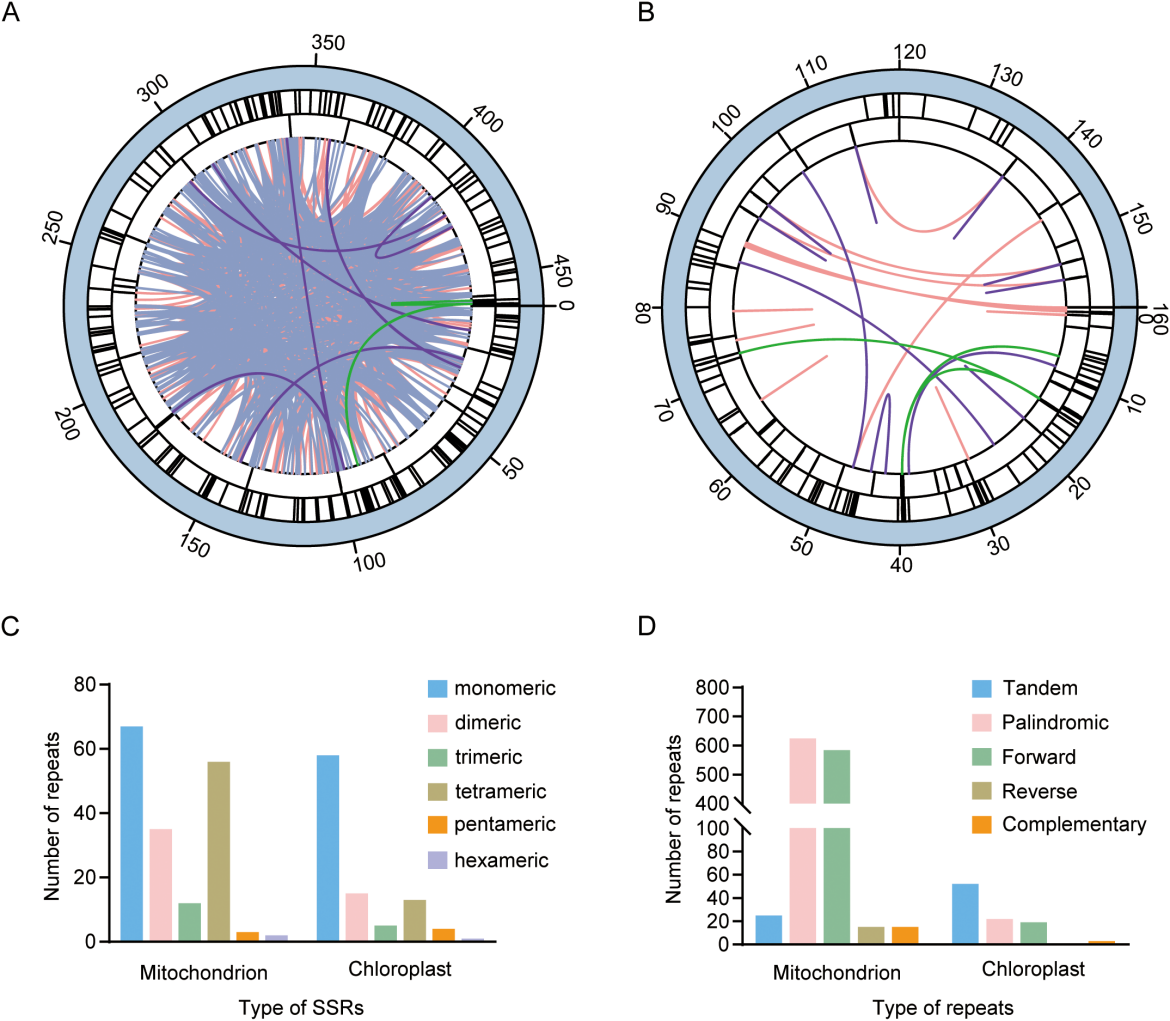
Repetitive sequences in the mitochondrial and chloroplast genomes of *J. spinosa*. **(A, B)** Distribution of repetitive sequences in the mitochondrial and chloroplast genomes. The lines in the outer, middle, and inner circles represent the distribution of SSRs, tandem repeats, and dispersed repeats in the genome, respectively. The green, purple, blue, and red lines in the innermost circle represent complementary repeats, forward repeats, palindromic repeats, and reverse repeats, respectively. **(C)** Types and numbers of SSRs. **(D)** Types and numbers of tandem and dispersed repeats.

We analyzed the distribution of SSRs, tandem repeats, and dispersed repeats in the mitochondrial and chloroplast genomes of *J.* *spinosa* and mapped their genomic locations (**Figures 2A, B**). In the mitochondrial genome, we identified 175 SSRs, 25 tandem repeats, and 1,238 dispersed repeats. The most abundant SSRs types were mononucleotide (67, 38.29%) and tetranucleotide (56, 32%), with 91.04% of the mononucleotide repeats consisting of A repeats. Among the dinucleotide repeats, AT repeats were the most frequent, accounting for 60%. Only three pentanucleotide and two hexanucleotide SSRs were detected (**Figure 2C**, **Supplementary Table S1**). Tandem repeats ranged from 2 to 57 bp in length (**Supplementary Table S2**). The dispersed repeats included 584 forward, 15 reverse, 15 complementary, and 642 palindromic repeats, ranging from 30 to 330 bp (**Figure 2D**; **Supplementary Table S3**).

In the chloroplast genome, we identified 96 SSRs, 52 tandem repeats, and 44 dispersed repeats. The SSRs were mainly composed of mononucleotide (60.42%) and dinucleotide (15.63%) repeats, with T mononucleotide repeats being the most abundant (50%) (**Figure 2C**, **Supplementary Table S4**). Only one hexanucleotide SSR was detected. Tandem repeats ranged from 2 to 38 bp in length (**Supplementary Table S5**). The dispersed repeats included 19 forward, 1 reverse, 2 complementary, and 22 palindromic repeats, ranging from 31 to 613 bp in length (**Figure 2D**, **Supplementary Table S6**). These repetitive sequences provide important resources for assessing genetic diversity and developing molecular markers in *J.* *spinosa*.

### 3.3 Codon usage analysis of PCGs

The mitochondrial and chloroplast genomes possess independent genetic systems, and analyzing their codon usage bias can provide important insights into evolutionary history and adaptive mechanisms (Murray et al., 1989). We analyzed codon usage patterns in both organelle genomes of *J.* *spinosa*. The 40 mitochondrial PCGs span 33,483 bp and encode 11,738 codons, while the 84 chloroplast PCGs span 78,576 bp and encode 26,192 codons. In both genomes, leucine (Leu) was the most frequently used amino acid (10.74%, 1,261 instances in the mitochondrion; 10.52%, 2,756 instances in the chloroplast), whereas cysteine (Cys) and tryptophan (Trp) were the least frequently used (1.45%/170 and 1.58%/186 in the mitochondrion; 1.15%/302 and 1.75%/458 in the chloroplast, respectively) (**Supplementary Tables S7**, **S8**).

**Figure 3:**
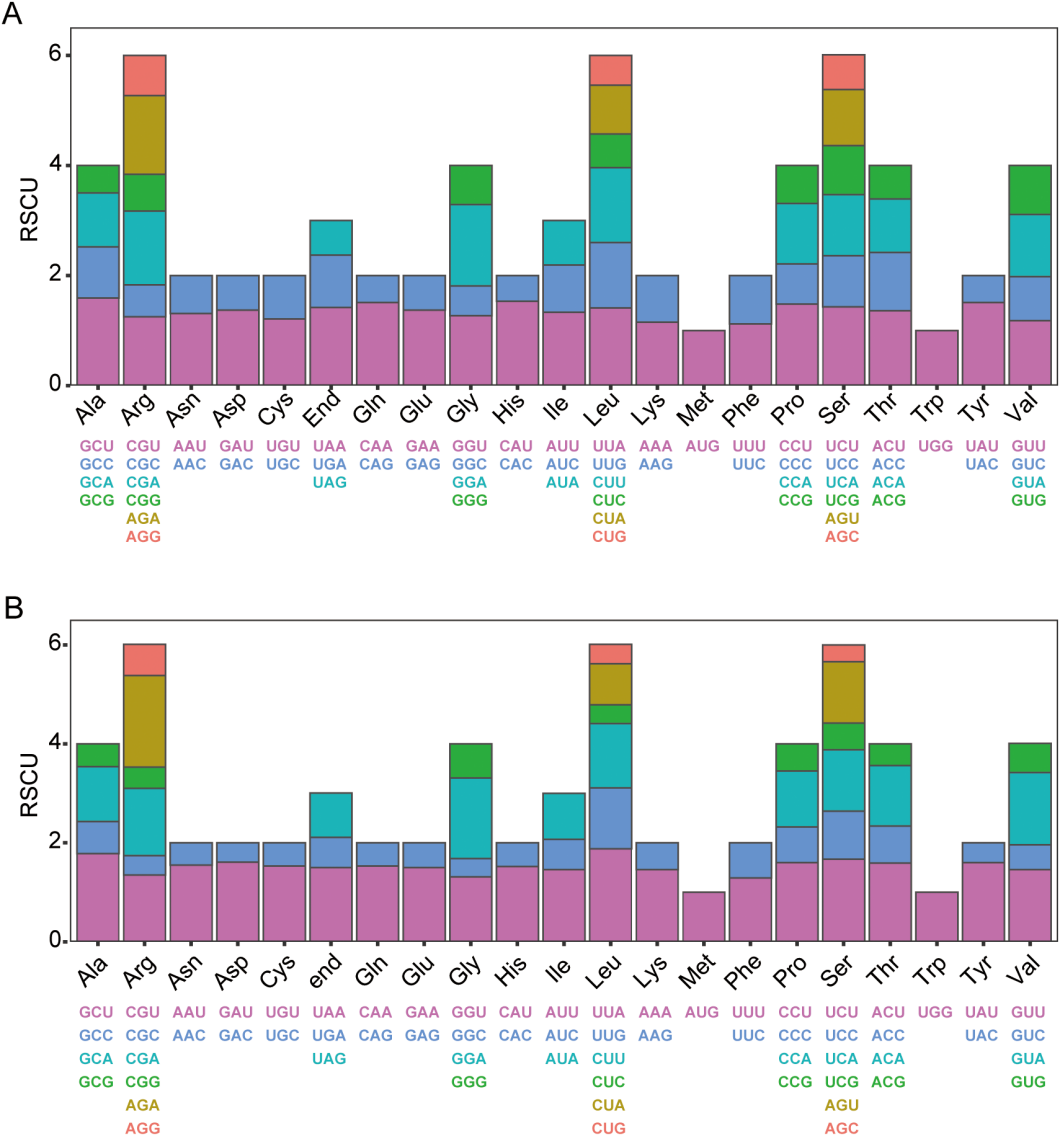
Codon preference of *J. spinosa*. **(A)** Mitochondria genome. **(B)** Chloroplast genomes. The x-axis shows amino acids, with their corresponding codons displayed below. The y-axis represents relative synonymous codon usage (RSCU), where a value of 1 indicates no codon bias, values >1 indicate preferential usage, and values <1 indicate lower usage. Different colors distinguish codons encoding the same amino acid.

Relative synonymous codon usage (RSCU) analysis identified 29 and 30 high-frequency codons (RSCU > 1) in the mitochondrial and chloroplast genomes, respectively. Among these, 3 mitochondrial and 7 chloroplast codons exhibited strong preference (RSCU > 1.5) (**Figures 3A, B**). In both genomes, UAA was the most preferred stop codon (**Supplementary Tables S7**, **S8**). The prevalence of A- or U-ending high-frequency codons reflects a pronounced codon usage bias, consistent with their AT-rich composition of *J.* *spinosa* organelle genomes.

Both mitochondrial and chloroplast genomes contain several non-canonical start and stop codons, prompting a detailed investigation. Although most PCGs initiate translation with the standard start codon ATG, notable exceptions were observed in the mitochondrial genome: *cox1, nad1, nad4L*, and *rps4* initiate with ACG; *rpl16* with GTG; and *mttB* with ATA. Stop codon usage was also diverse, with five different types identified. While most genes terminate with typical stop codons (TAA TAG and TGA), *atp6* ends with CAA, and *atp9* and *ccmFC* used non-standard codon CGA.

In contrast, the chloroplast genome exhibited greater conservation. All PCGs used ATG as the start codon, except *rps19*, which initiates with GTG. All genes terminated with standard stop codons. Overall, codon usage patterns in *J.* *spinosa* closely resembled those of *C. papaya*, suggesting strong conservation of mitochondrial protein-coding mechanisms as a high degree of functional and evolutionary consistency between *J.* *spinosa* and *C. papaya*.

### 3.4 RNA editing sites prediction

**Figure 4:**
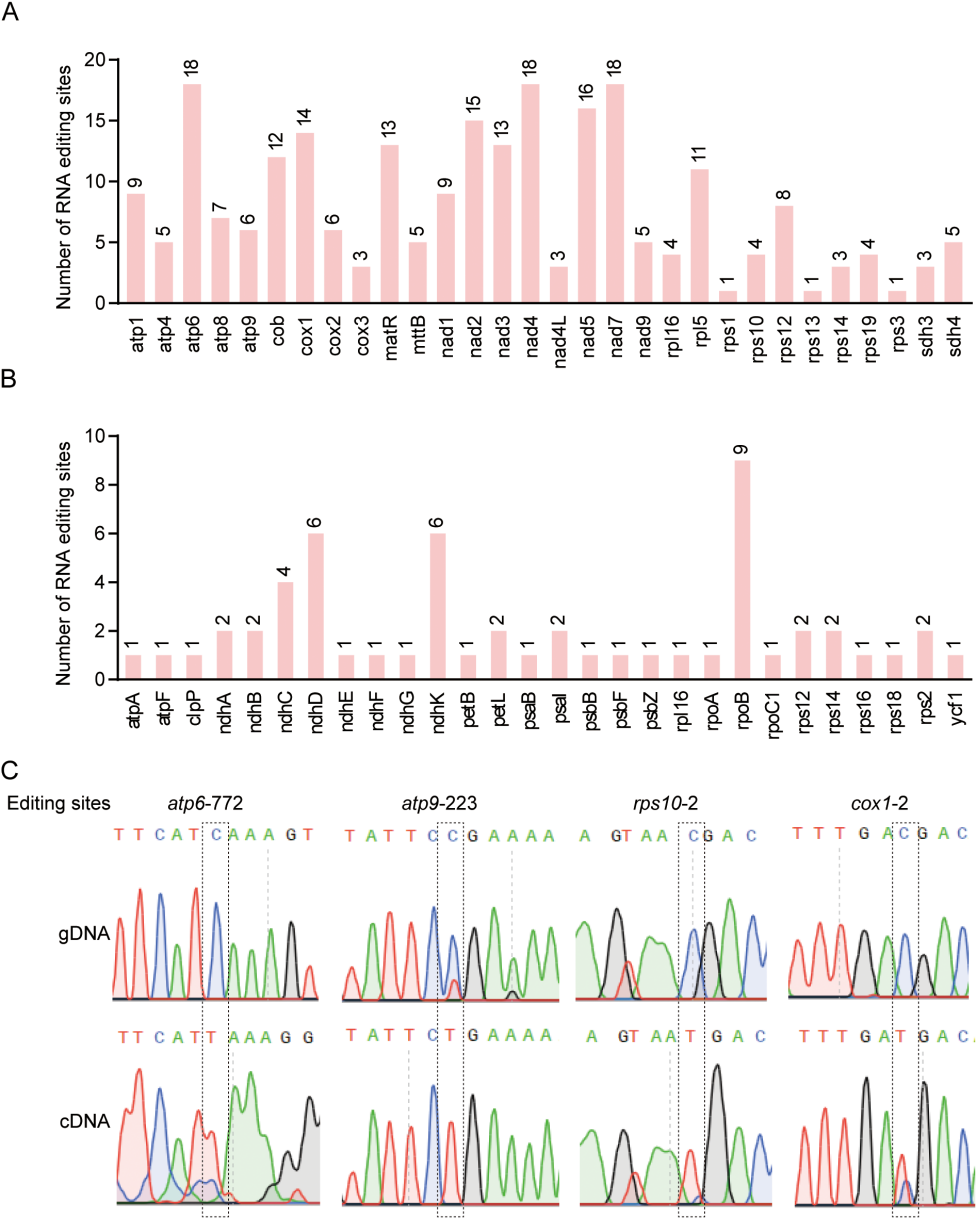
Statistical analysis and validation of RNA editing sites in mitochondria and chloroplasts **(A, B)** The RNA editing sites in PCGs of mitochondrial and chloroplast genome predicted by the BEDTools software. **(C)** Genomic PCR and Sanger sequencing validation of RNA editing sites located in the start and stop codons of target genes (*atp6 atp9 rps10 cox1*).

RNA editing events, particularly cytidine-to-uridine (C-to-U) conversions, are widespread and highly enriched in plant organellar genomes. In *J.* *spinos*a, a total of 349 and 73 RNA editing sites were identified in the mitochondrial and chloroplast genomes, respectively. Among these, 240 sites in the mitochondrion and 56 in the chloroplast were located within protein-coding regions (**Supplementary Tables S9**, **S10**). In the mitochondrial genome, the highest numbers of editing sites were observed in *atp6, nad7, nad4*, and *nad5* (**Figure 4A**), while in the chloroplast genome, most editing events were concentrated in *rpoB, ndhD*, and *ndhK* genes (**Figure 4B**).

In PCGs, RNA editing predominantly occurred at the second codon position, with 149 events (62.08%) identified in the mitochondrion and 39 events (69.64%) in the chloroplast. In contrast, the third codon position exhibited the lowest frequency of editing, with only 20 events (8.33%) in the mitochondria and 5 events (8.93%) in the chloroplast. Among all nonsynonymous editing events, the most frequent amino acid substitution is from serine (Ser) to leucine (Leu), observed 56 times (23.33%) in the mitochondria and 20 times (35.71%) in the chloroplast (**Supplementary Tables S9**, **S10**). This trend indicates a bias toward the conversion of hydrophilic to hydrophobic residues, suggesting that Ser codons are major targets of RNA editing.

In addition, RNA editing plays a key role in restoring functional start and stop codons. Several non-canonical start or stop codons in *J.* *spinosa*, including *atp6* (CAA→TAA), *atp9* (CGA→TGA), *rps10* (ACG→ATG), and *cox1* (ACG→ATG), were corrected via RNA editing. These editing events were validated by PCR using gene-specific primers, confirming the authenticity (**Figure 4C**). Collectively, these results underscore the essential regulatory role of RNA editing in translation initiation and termination in *J.* *spinosa* organelle genomes.

### 3.5 Gene transfer between mitochondrial and chloroplast genomes

**Figure 5:**
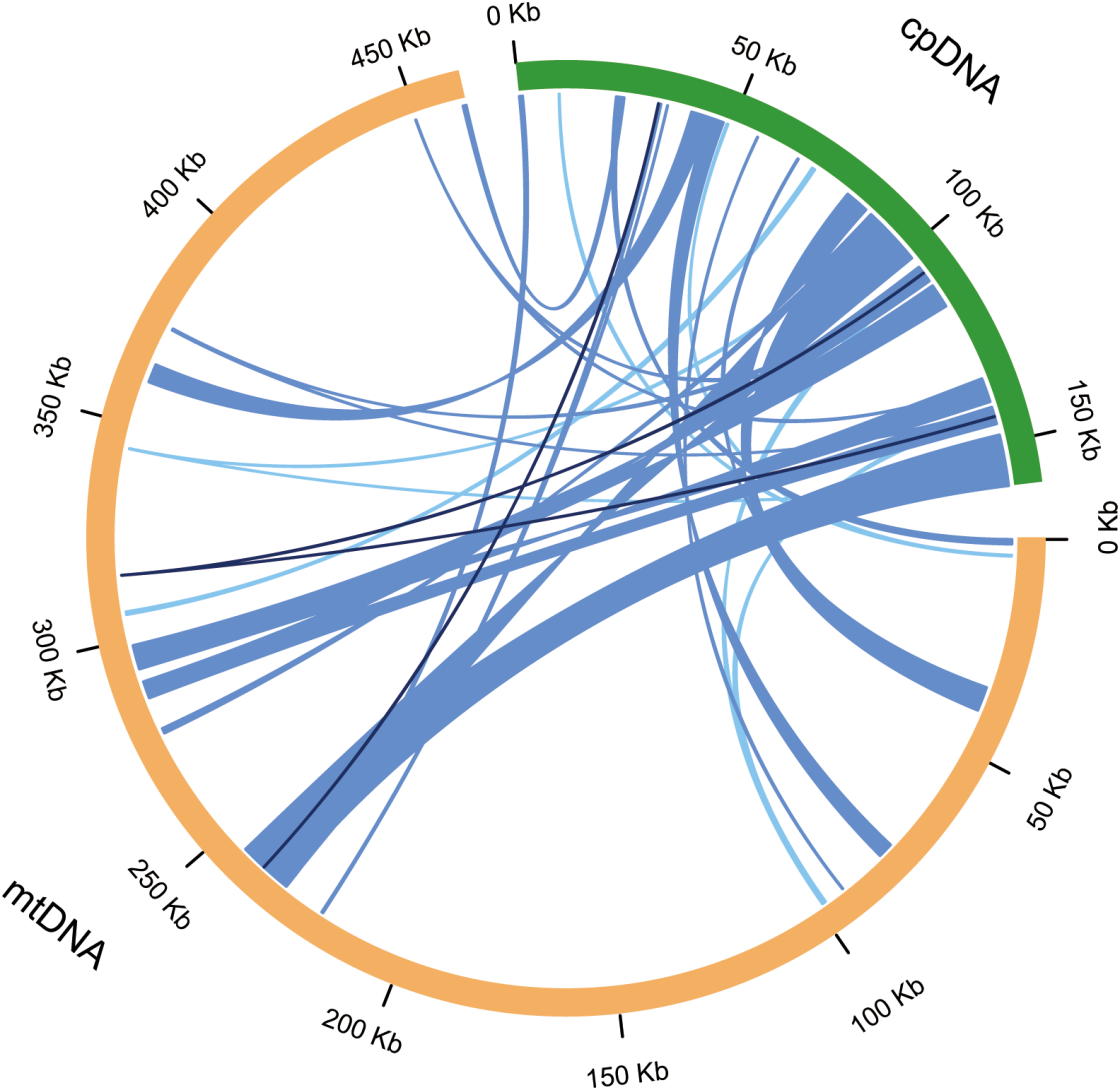
Homologous fragment analysis based on different organelle genomes. The orange and green outer arcs represent the mitochondrial (mtDNA) and chloroplast (cpDNA) genomes, respectively. The inner connecting lines between the arcs represent homologous DNA fragments, with different colors indicating varying thresholds of sequence similarity: dark blue denotes 100% similarity, blue indicates similarity greater than 90%, and light blue represents similarity greater than 70%.

Based on sequence similarity between the two organellar genomes, 34 highly homologous regions were identified, likely representing chloroplast-derived fragments integrated into the mitochondrial genome. These transferred regions ranged from 29 to 12,675 bp in length, and were dispersed across the mitochondrial genome, and collectively accounted for 65,352 bp—comprising 14.16% of the mitochondrial genome and 40.85% of the chloroplast genome (**Figure 5**, **Supplementary Table S11**). The two longest fragments, both exceeding 12 kb, originated from the inverted repeat (IR) regions of the chloroplast genome and were inserted into mitochondrial loci encompassing *trnI-CAU* and *trnL-CAA*.

Among the transferred sequences, 19 were located in intergenic regions, 13 were found in tRNA regions, and 2 were mapped to protein-coding regions. A total of 11 tRNA genes were identified as completely transferred, including *trnD-GUC, trnH-GUG, trnI-CAU, trnI-GAU, trnL-CAA, trnM-CAU, trnN-GUU, trnR-ACG, trnS-GGA, trnV-GAC*, and *trnW-CCA*. No genes were interrupted. These results provide valuable molecular evidence for the frequent exchange of genetic material between organelles and offer new insights into the evolutionary dynamics of plant organellar genomes.

### 3.6 Phylogenetic and selective pressure analysis

**Figure 6:**
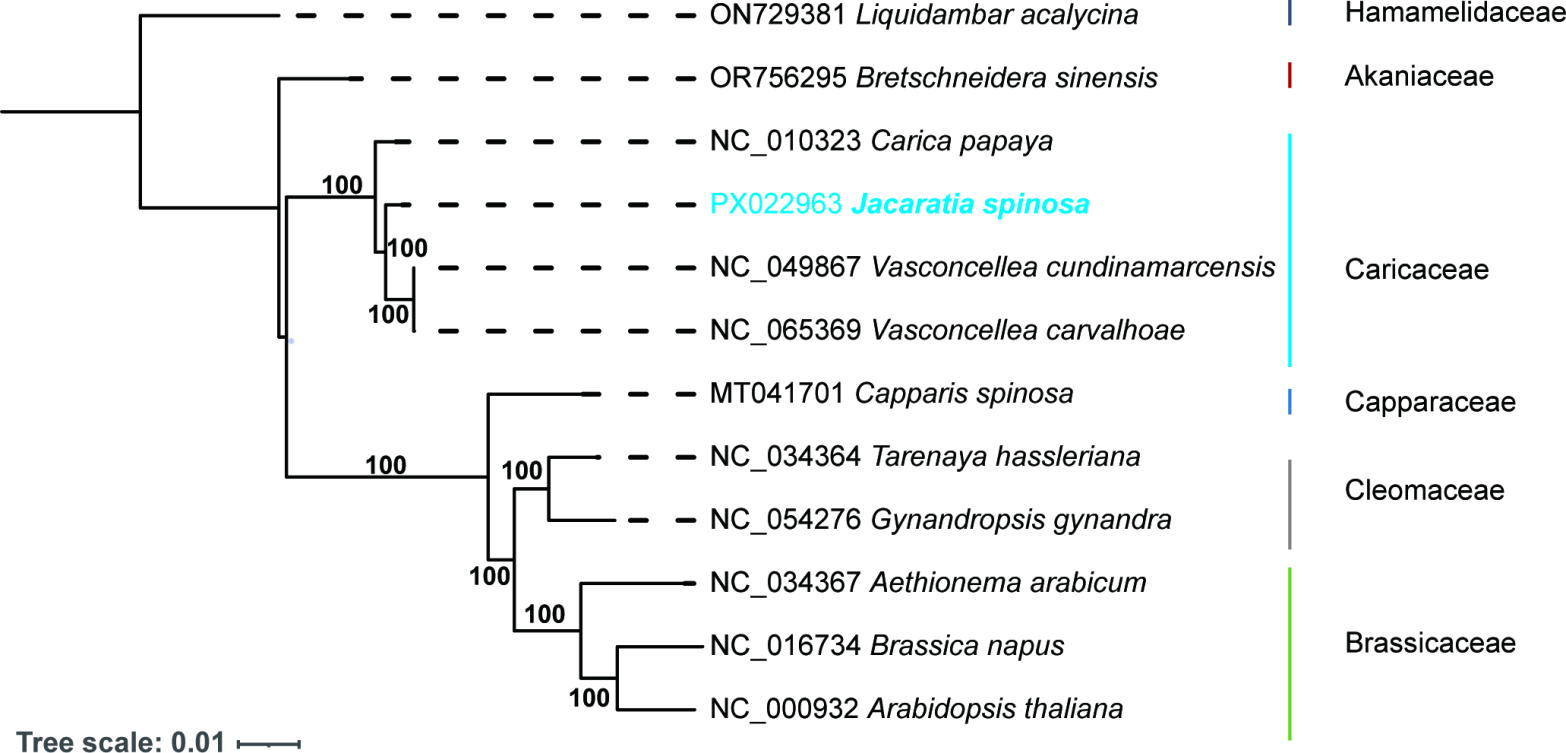
The phylogenetic relationships between *J. spinosa* and 11 *Brassicales* species were inferred using the maximum likelihood (ML) method based on shared single-copy orthologous genes from the chloroplast genomes. The position of *J.* *spinosa* and its evolutionary relationship within the Caricaceae are highlighted in light blue.

Due to the limited number of reported mitochondrial genomes within the family Caricaceae, the phylogenetic position of *J.* *spinosa* was inferred based on chloroplast genomes. In this study, 69 shared PCGs were extracted from the chloroplast genomes of 12 published species within the order Brassicales (**Supplementary Table S12**). A maximum likelihood (ML) phylogenetic tree was constructed, with *Liquidambar acalycina* (Hamamelidaceae), a deciduous tree from the genus Liquidambar, used as the outgroup. The resulting phylogeny revealed that *J.* *spinosa* formed a supported sister clade with *C. papaya*, indicating their close evolutionary relationship at the chloroplast genome level. This clade further grouped with *V. cundinamarcensis* and *V. carvalhoae*, forming a monophyletic branch representing the Caricaceae (**Figure 6**). Notably, *Carica, Vasconcellea*, and *Jacaratia* represent three distinct genera within Caricaceae, suggesting that the present analysis not only supports the monophyly of the family but also provides new insights into intergeneric relationships. Although Caricaceae and Brassicaceae both belong to the Brassicales, they formed separate and distantly related monophyletic clades in the phylogenetic tree, reflecting substantial divergence at the chloroplast genome level.

**Figure 7:**
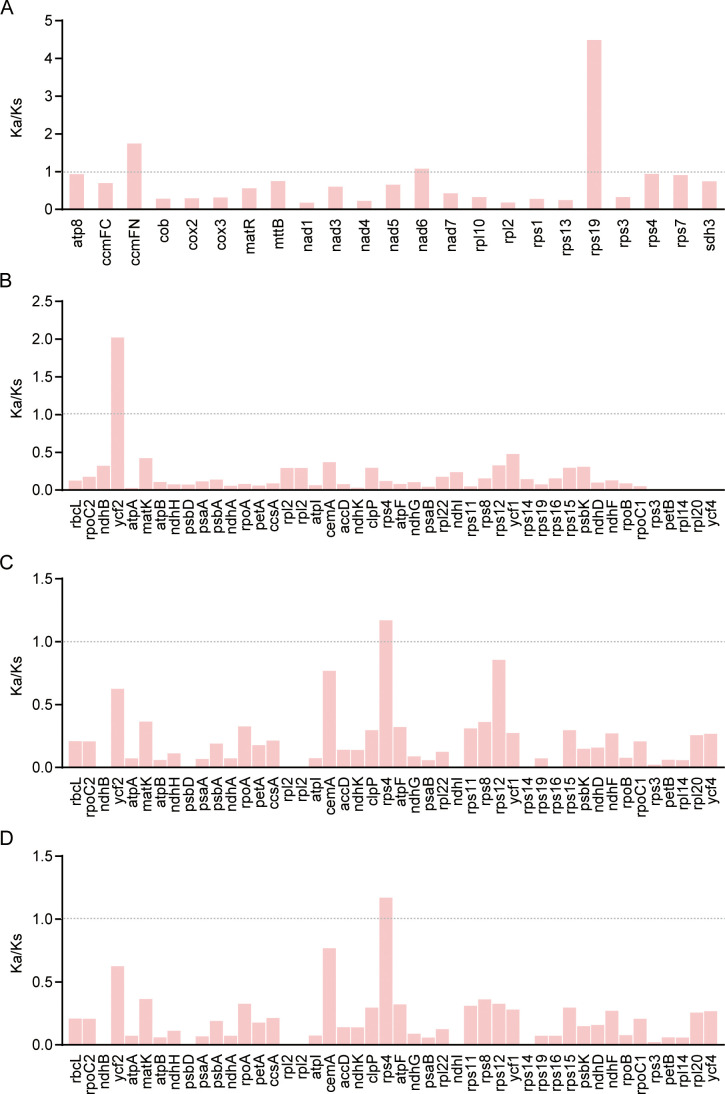
Ka/Ks analysis of mitochondrial and chloroplast genes **(A)** Comparison of the Ka/Ks ratios of mitochondrial PCGs between *J. spinosa* and*C. papaya*. **(B)** Comparison of Ka/Ks ratios of chloroplast PCGs among *J. spinosa*, *C. papaya.***(C)** Comparison of Ka/Ks ratios of chloroplast PCGs among *J. spinosa* and *V. cundinamarcensis*. **(D)** Comparison of Ka/Ks ratios of chloroplast PCGs among *J. spspinosa* and *V. carvalhoae*.

Based on 23 single-copy orthologous mitochondrial genes shared between *J.* *spinosa* and *C. papaya*, we calculated the nonsynonymous substitution rate (Ka), the synonymous substitution rate (Ks), and their ratio (Ka/Ks). Of these, 20 genes exhibited Ka/Ks values less than 1, indicating that most mitochondrial PCGs have undergone strong purifying selection and remain relatively conserved during evolution. However, *ccmFN* and *rps19* exhibited Ka/Ks values greater than 1, suggesting potential positive selection or functional divergence, possibly reflecting lineage-specific adaptive evolution within the Caricaceae (**Figure 7A**).

In contrast, chloroplast PCGs appeared to be more conserved. To further investigate this, we performed Ka/Ks analysis on 39 single-copy orthologous genes shared between *J.* *spinosa* and three other Caricaceae species. The results showed that *ycf2* had a Ka/Ks value greater than 1 when compared with *C. papaya*, while *rps4* exhibited a Ka/Ks value greater than 1 when compared with the two *Vasconcellea* species. All remaining genes showed Ka/Ks values less than 1 (**Figure 7B**). These findings reinforced that most chloroplast PCGs were under purifying selection, although a few genes may be subject to positive selection.

### 3.7 Synteny analysis

**Figure 8:**
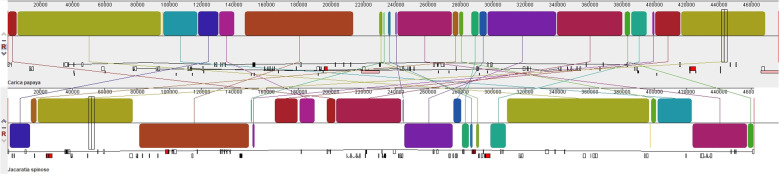
Synteny analysis between *J. spinosa* and *C. papaya* using the Mauve software. The differently colored lines between the two sequences represent syntenic blocks, illustrating genome rearrangements between the two species.

We conducted a synteny analysis of the mitochondrial genomes between *J.* *spinosa* and *C. papaya*, identifying 65 homologous regions ranging from 152 to 24,612 bp in length. These regions collectively accounted for 410,307 bp, representing 88.87% of the *J.* *spinosa* mitochondrial genome. In addition, 47 species-specific regions were detected, ranging from 19 to 12,075 bp and totaling 50,519 bp, which represent 10.94% of the genome. Notably, 14 syntenic blocks exceeded 10,000 bp in length, indicating a high degree of sequence similarity between the two mitochondrial genomes (**Figure 8**). Despite this extensive homology, the overall syntenic structure revealed considerable genomic rearrangements, suggesting that substantial structural reconfiguration has occurred during mitochondrial genome evolution in the Caricaceae.

**Figure 9:**
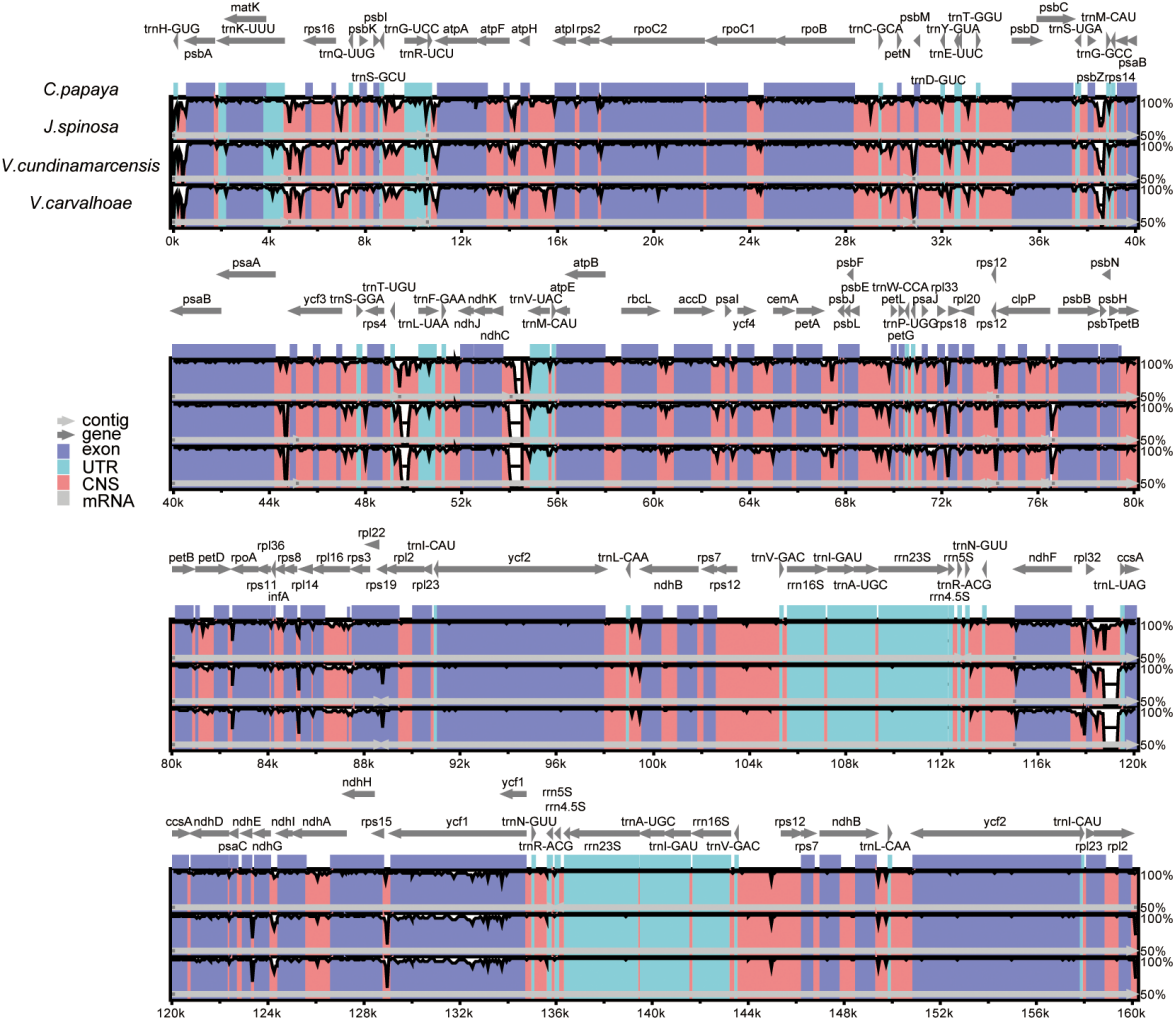
Sequence variation among *J. spinosa*, *C. papaya*, *V. cundinamarcensis*, and *V. carvalhoae* was analyzed using mVISTA with *C. papaya* as the reference. Gray arrows and thick lines indicate gene orientation. Purple bars represent exons, blue bars UTRs, pink bars conserved non-coding sequences (CNSs), and gray bars mRNA. The y-axis shows sequence identity from 50% to 100%, genes with significant variation are labeled in red, and the corresponding highly variable regions are outlined with white dashed boxes.

We also performed a synteny analysis of the chloroplast genomes of *C. papaya*, *J.* *spinosa* and the previously published *V. cundinamarcensis* and *V. carvalhoae*. Comparative analyses using mVISTA revealed that a high degree of sequence conservation among the three genera, consistent with their close phylogenetic relationship. However, notable sequence variations were observed in several conserved non-coding sequences (CNSs) as well as in genic regions such as *ycf1*, *rps19*, and *rpoC2* (**Figure 9**), analysis of structural variation among species based on the extracted *ycf1*, *rps19*, and *rpoC2* gene sequences showed that the variation levels of *ycf1* and *rpoC2* support the close phylogenetic relationship between *C. papaya* and *J.* *spinosa*, which is consistent with the phylogenetic results. (**Supplementary Figure S2**, **Supplementary Table S13**). Furthermore, highly variable CNS regions located at 49 kb, 54 kb, and 119 kb also exhibited variation patterns consistent with the phylogenetic relationships, providing additional support for these findings (**Supplementary Figure S3**). The significance of these variations includes providing molecular evidence for the phylogenetic relationship between *C. papaya* and *J.* *spinosa*. They also reflect differences in selective pressures on conserved and variable regions during species evolution, which aids in understanding intergeneric divergence. In addition, certain genes (*ycf1* and *rpoC2*) may play key roles in chloroplast function, offering references for functional studies. Finally, highly variable CNS regions may serve as potential molecular markers for comparative genomics and breeding research.

## 4 Discussion

Mitochondria and chloroplasts serve as the primary sites for cellular respiration and photosynthesis, respectively, and are indispensable functional cores essential for plant growth and development (Millar et al., 2011; Neuhaus and Emes, 2000). Notably, both types of organelles possess semi-autonomous genomes distinct from the nuclear genome, characterized by unique structural and functional properties. The continuous decline in high-throughput sequencing costs has facilitated the sequencing and publication of organellar genomes for an increasing number of plant species, significantly enriching the available genomic resources (Sawicki et al., 2024).

By integrating third-generation and second-generation sequencing data, we successfully assembled complete and high-quality mitochondrial and chloroplast genomes of *J.* *spinosa*. The results revealed that both organellar genomes constitute single, circular DNA molecules. This conformation is consistent with that observed in its close relative, *C. papaya* (Rice et al., 2008). Specifically, the *J.* *spinosa* mitochondrial genome is 461,675 bp in size, while its chloroplast genome is 160,000 bp (**Tables 1** and **2**). Both genomes are slightly smaller than their counterparts in *C. papaya* (mitochondrion: 476,890 bp; chloroplast: 160,100 bp) (Rice et al., 2008). Notably, the *J.* *spinosa* chloroplast genome is slightly larger than those of two representative species of the genus *Vasconcellea*, namely *V. cundinamarcensis* (158,712 bp) and *V. carvalhoae* (158,723 bp) (Lin et al., 2020; Tineo et al., 2022). Based on the phylogenetic analysis (**Figure 5**), *C. papaya* shares a closer evolutionary relationship with *J.* *spinosa* than with species of *Vasconcellea*, consistent with the family-wide phylogeny *Carvalho* and *Renner* (Carvalho and Renner, 2012). This pattern is to some extent mirrored by the differences in organellar genome sizes.

In most closely related species, the number and composition of chloroplast genome-encoded genes are typically conserved (Dobrogojski et al., 2020), whereas mitochondrial genomes exhibit a higher degree of variation—a pattern also observed in *J.* *spinosa*. In the mitochondrial genome of *J.* *spinosa*, a total of 69 genes were annotated, showing slight divergence from the 66 genes identified in *C. papaya* (**Supplementary Table S14**). Comparative analysis revealed that *J.* *spinosa* lacks one copy of the *ccmFN* gene present in *C. papaya*, but contains four additional tRNA genes, *trnI-GAU, trnR-ACG, trnV-GAC*, and *trnN-GUU*. BLAST-based alignment of the *C. papaya* mitochondrial genome identified two 11,105 bp regions exhibiting complete sequence identity, indicative of a local duplication event (**Supplementary Table S15**). This structural duplication likely accounts for the increased copy number of the *ccmFN* gene in *C. papaya* relative to its close relative *J.* *spinosa*, in which no comparable duplicated region was detected.

Repetitive sequences are abundant in plant organellar genomes, where they play crucial roles in shaping genome structure and driving evolutionary processes (Gualberto and Newton, 2017). In the mitochondrial genomes of *Silene* and *Cucumber*, a large number of repetitive sequences and gene redundancies have been identified, which are important contributors to mitochondrial genome expansion (Alverson et al., 2011; Gualberto and Newton, 2017; Sloan et al., 2012). Among these, SSRs are particularly valuable for the development of molecular markers (Cui et al., 2008; Provan et al., 2001). In the mitochondrial genome of *J.* *spinosa*, a total of 175 SSRs, 25 tandem repeats, and 1,238 dispersed repeats were identified (**Figure 2C**). Mononucleotide repeats are predominantly composed of A/T bases, reflecting the typical base composition bias observed in plant mitochondrial genomes (Smith and Keeling, 2015). Most dispersed repeats are in direct or palindromic orientations, potentially providing sequence substrates for genome recombination. In contrast, the chloroplast genome contains fewer repeats, including 96 SSRs, 52 tandem repeats, and 44 dispersed repeats (**Figures 2C, D**). T mononucleotide repeats are the most common, indicating a distinct base composition characteristic of the chloroplast genome.

The mitochondrial genome of *J.* *spinosa* has undergone active inter-organelle DNA transfer and recombination events. In this study, 34 highly similar chloroplast-derived fragments were identified integrated into its mitochondrial genome (**Figure 5**), accounting for 14.16% of the total length, which is significantly higher than the approximately 1% reported in *Arabidopsis thaliana* (Unseld et al., 1997), and also exceeds the chloroplast fragment coverage ratios of about 11% and 10% observed in cucumber and date palm, respectively (Alverson et al., 2011; Fang et al., 2012). These transferred sequences include seven structurally intact tRNA genes, reflecting both the extent and the preservation of transferred chloroplast sequences (**Supplementary Table S11**). Notably, the *J.* *spinosa* mitochondrial genome contains 4 additional chloroplast-derived tRNA genes (*trnI-GAU, trnR-ACG, trnV-GAC*, and *trnN-GUU*) compared to *C. papaya*. Such inter-organelle tRNA gene transfers are widespread in plants, especially in species where mitochondrial tRNA genes are severely lost, and the integration of foreign tRNAs helps maintain the integrity of the translational system (Warren and Sloan, 2020).

The codon usage of *J.* *spinosa* organellar genes reflects both the common patterns observed in terrestrial plants and certain species-specific features. Analysis revealed a strong preference for codons ending in A/U in both mitochondrial and chloroplast genomes (**Figure 3**), indicating a typical AT bias consistent with the mutation pressure and base substitution trends commonly found in land plants (Morton, 1998; Zhang et al., 2024). High-frequency codons are mostly associated with abundant amino acids such as leucine (Leu), while cysteine (Cys) and tryptophan (Trp) are used least frequently (**Supplementary Tables S7**, **S8**), likely due to their high biosynthetic cost and limited tRNA availability (Hershberg and Petrov, 2008; Plotkin and Kudla, 2011). Several non-canonical start codons (ACG, GTG, ATA) and atypical stop codons (CAA, CGA) were identified in mitochondrial protein-coding genes (PCGs) (**Figure 4C**), suggesting potential complexity in translation initiation and termination, possibly influenced by RNA editing or unique initiation mechanisms (Giegé and Brennicke, 1999; Small et al., 2020), as supported by predicted and validated RNA editing sites. In contrast, codon usage in the chloroplast genome is highly conserved, reflecting stronger purifying selection on photosynthesis-related gene expression (Wicke et al., 2011). Furthermore, the high similarity in codon usage patterns between *J.* *spinosa* and *C. papaya* underscores the evolutionary conservation of their organellar protein-coding systems (Morton, 1998).

The phylogenetic tree that was constructed based on 69 shared protein-coding genes from the chloroplast genome revealed a close relationship between *J.* *spinosa* and *C. papaya*, supporting the monophyly of the Caricaceae family (**Figure 6**). This finding is consistent with morphological characteristics and nuclear gene-based classifications and, for the first time, clarifies the systematic position of *J.* *spinosa* at the molecular level (Carvalho and Renner, 2012; Rockinger et al., 2016). The distinct divergence among the three genera suggests a stable evolutionary framework within the family. In addition, although currently only mitochondrial genome data for *J.* *spinosa* and *C. papaya* are available, these two species exhibit high similarity in gene content and structure (Rice et al., 2008). This result is consistent with the topology of the chloroplast phylogeny and provides preliminary mitochondrial molecular support for the monophyly of Caricaceae. However, due to the lack of mitochondrial data for other genera, it is not yet possible to construct a complete mitochondrial phylogeny focusing on the family. Therefore, the role of mitochondrial genomes in fully resolving the internal evolutionary relationships of Caricaceae still requires validation with additional species data.

Selective pressure analysis showed that most mitochondrial and chloroplast protein-coding genes (PCGs) had Ka/Ks values less than 1, indicating strong purifying selection and reflecting their conserved roles in core metabolic functions (Sloan et al., 2013). Notably, mitochondrial genes *ccmFN* and *rps19*, as well as chloroplast genes *ycf2* and *rps4*, exhibited Ka/Ks values greater than 1 (**Figures 7A, D**), suggesting they may be under positive selection or undergoing functional innovation. These genes are involved in cytochrome synthesis, ribosome assembly, and protein transport, and may be subject to lineage-specific selective pressures, warranting further functional validation (Kikuchi et al., 2018; Meyer et al., 2005; Tiller and Bock, 2014). Synteny analysis demonstrated high structural conservation between *J.* *spinosa* and *C. papaya* in both mitochondrial and chloroplast genomes, with mitochondrial syntenic regions accounting for 88.87% (**Figure 8**). However, structural rearrangements mediated by repeat sequences were observed (Raubeson and Jansen, 2005). mVISTA comparisons further revealed that, despite overall sequence conservation, notable differences existed in certain conserved non-coding sequences (CNSs) and functional genes (*ycf1, rps19, rpoC2*), which may have adaptive significance (**Figure 9**). Among these, *ycf1*, known for its rapid evolutionary rate, holds promise as a molecular marker for phylogenetic and species identification studies.

## 5 Conclusions

Here, we successfully sequenced and assembled the mitochondrial and chloroplast genomes of *J.* *spinosa* for the first time. The mitochondrial genome exhibits a circular structure with a total length of 461,675 bp, while the chloroplast genome adopts a typical quadripartite structure with a length of 160,000 bp. Comprehensive analyses of gene content, repetitive sequences, codon usage bias, and RNA editing sites were conducted. The results revealed that the mitochondrial PCGs of *J.* *spinosa* contain numerous non-canonical start and stop codons, some of which can be restored to canonical codons through RNA editing. Moreover, extensive gene transfer and sequence similarity were observed between the mitochondrial and chloroplast genomes. Based on the complete chloroplast genome, the phylogenetic position of *J.* *spinosa* was determined. Selection pressure analysis further identified genes under positive selection during the evolution of *J.* *spinosa*. Overall, this study enriches the genomic resources of the Caricaceae family and provides valuable insights into the phylogeny and evolutionary dynamics of its member species.

## Data Availability

The datasets generated for this study are available in online repositories. The raw sequencing data have been deposited in the NCBI Sequence Read Archive (SRA) under BioProject accession number PRJNA1299754 and BioSample accession number SAMN50313822, with SRA accession numbers SRR34828180 and SRR34828179. In addition, corresponding annotated genome information has been submitted to GenBase at NGDC/CNCB and is available under accession number CAA121889.1 CAA129751.1 (https://ngdc.cncb.ac.cn/genbase) (Bu et al., 2024). https://dataview.ncbi.nlm.nih.gov/object/PRJNA1299754?reviewer=h0aikutsltumhocntr3v9514th.

## References

[B1] Altschul S. F. Gish W. Miller W. Myers E. W. Lipman D. J. (1990). Basic local alignmentsearch tool. J. Mol. Biol. 215, 403–410. doi: 10.1016/S0022-2836(05)80360-2, PMID: 2231712

[B2] Alverson A. J. Rice D. W. Dickinson S. Barry K. Palmer J. D. (2011). Origins and recombination of the bacterial-sized multichromosomal mitochondrial genome of cucumber. Plant Cell 23, 2499–2513. doi: 10.1105/tpc.111.087189, PMID: 21742987 PMC3226218

[B3] Benson G. (1999). Tandem repeats finder: a program to analyze DNA sequences. Nucleic Acids Res. 27, 573–580. doi: 10.1093/nar/27.2.573, PMID: 9862982 PMC148217

[B4] Bi C. Shen F. Han F. Qu Y. Hou J. Xu K. . (2024). PMAT: an efficient plant mitogenome assembly toolkit using low-coverage HiFi sequencing data. Hortic. Res. 11, uhae023. doi: 10.1093/hr/uhae023, PMID: 38469379 PMC10925850

[B5] Bu C. Zheng X. Zhao X. Xu T. Bai X. Jia Y. . (2024). GenBase: A nucleotide sequence database. Genomics Proteomics Bioinf. 22, qzae047. doi: 10.1093/gpbjnl/qzae047, PMID: 38913867 PMC11434157

[B6] Carvalho F. A. Renner S. S. (2012). A dated phylogeny of the papaya family (Caricaceae) reveals the crop’s closest relatives and the family’s biogeographic history. Mol. Phylogenet. Evol. 65, 46–53. doi: 10.1016/j.ympev.2012.05.019, PMID: 22659516

[B7] Chen C. Chen H. Zhang Y. Thomas H. R. Frank M. H. He Y. . (2020). TBtools: An integrative toolkit developed for interactive analyses of big biological data. Mol. Plant 13, 1194–1202. doi: 10.1016/j.molp.2020.06.009, PMID: 32585190

[B8] Chen S. (2023). Ultrafast one-pass FASTQ data preprocessing, quality control, and deduplication using fastp. Imeta 2, e107. doi: 10.1002/imt2.107, PMID: 38868435 PMC10989850

[B9] Chen Y. Nie F. Xie S. Q. Zheng Y. F. Dai Q. Bray T. . (2021). Efficient assembly of nanopore reads via highly accurate and intact error correction. Nat. Commun. 12, 60. doi: 10.1038/s41467-020-20236-7, PMID: 33397900 PMC7782737

[B10] Cui X. M. Dong Y. X. Hou X. L. Cheng Y. Zhang J. Y. Jin M. F. (2008). Development and characterization of microsatellite markers in *Brassica rapa* ssp. chinensis and transferability among related species. Agr. Sci. China 7, 19–31. doi: 10.1016/S1671-2927(08)60018-8

[B11] Danecek P. Bonfield J. K. Liddle J. Marshall J. Ohan V. Pollard M. O. . (2021). Twelve years of SAMtools and BCFtools. GigaScience 10, giab008. doi: 10.1093/gigascience/giab008, PMID: 33590861 PMC7931819

[B12] Darling A. C. Mau B. Blattner F. R. Perna N. T. (2004). Mauve: multiple alignment of conserved genomic sequence with rearrangements. Genome Res. 14, 1394–1403. doi: 10.1101/gr.2289704, PMID: 15231754 PMC442156

[B13] De Coster W. Rademakers R. (2023). NanoPack2: population-scale evaluation of long-read sequencing data. Bioinformatics 39. doi: 10.1093/bioinformatics/btad311, PMID: 37171891 PMC10196664

[B14] Dobrogojski J. Adamiec M. Luciński R. (2020). The chloroplast genome: a review. Acta Physiol. Plant 42, 98. doi: 10.1007/s11738-020-03089-x

[B15] Doyle J. J. Doyle J. L. (1987). A rapid DNA isolation procedure for small quantities of fresh leaf tissue. Phytochem. Bull. 19, 11–15.

[B16] Dyall S. D. Brown M. T. Johnson P. J. (2004). Ancient Invasions: From endosymbionts to organelles. Science 304, 253–257. doi: 10.1126/science.1094884, PMID: 15073369

[B17] Fang Y. Wu H. Zhang T. Yang M. Yin Y. Pan L. . (2012). A complete sequence and transcriptomic analyses of date palm (*Phoenix dactylifera L.*) mitochondrial genome. PloS One 7, e37164. doi: 10.1371/journal.pone.0037164, PMID: 22655034 PMC3360038

[B18] Frazer K. A. Pachter L. Poliakov A. Rubin E. M. Dubchak I. (2004). VISTA: computational tools for comparative genomics. Nucleic Acids Res. 32, W273–W279. doi: 10.1093/nar/gkh458, PMID: 15215394 PMC441596

[B19] Giegé P. Brennicke A. (1999). RNA editing in *Arabidopsis* mitochondria effects 441 C to U changes in ORFs. Proc. Natl. Acad. Sci. 96, 15324–15329. doi: 10.1073/pnas.96.26.15324, PMID: 10611383 PMC24818

[B20] Gray M. W. (1992). The endosymbiont hypothesis revisited. Int. Rev. Cytol. 141, 233–357. doi: 10.1016/S0074-7696(08)62068-9, PMID: 1452433

[B21] Greiner S. Lehwark P. Bock R. (2019). OrganellarGenomeDRAW (OGDRAW) version 1.3.1: expanded toolkit for the graphical visualization of organellar genomes. Nucleic Acids Res. 47, W59–W64. doi: 10.1093/nar/gkz238, PMID: 30949694 PMC6602502

[B22] Gualberto J. M. Newton K. J. (2017). Plant Mitochondrial Genomes: Dynamics and mechanisms of mutation. Annu. Rev. Plant Biol. 68, 225–252. doi: 10.1146/annurev-arplant-043015-112232, PMID: 28226235

[B23] Hanson M. R. Bentolila S. (2004). Interactions of mitochondrial and nuclear genes that affect male gametophyte development. Plant Cell 16, S154–S169. doi: 10.1105/tpc.015966, PMID: 15131248 PMC2643387

[B24] Hershberg R. Petrov D. A. (2008). Selection on codon bias. Annu. Rev. Genet. 42, 287–299. doi: 10.1146/annurev.genet.42.110807.091442, PMID: 18983258

[B25] Hu J. Fan J. Sun Z. Liu S. (2020). NextPolish: a fast and efficient genome polishing tool for long-read assembly. Bioinformatics 36, 2253–2255. doi: 10.1093/bioinformatics/btz891, PMID: 31778144

[B26] Huang K. Xu W. Hu H. Jiang X. Sun L. Zhao W. . (2025). Super-large record-breaking mitochondrial genome of *Cathaya argyrophylla* in Pinaceae. Front. Plant Sci. 16. doi: 10.3389/fpls.2025.1556332, PMID: 40612618 PMC12222119

[B27] Jansen R. K. Ruhlman T. A. (2012). “ Plastid genomes of seed plants,” in Genomics of chloroplasts and mitochondria. Advances in Photosynthesis and Respiration (Dordrecht: Springer), 103–126. doi: 10.1007/978-94-007-2920-9_5

[B28] Jin J. J. Yu W. B. Yang J. B. Song Y. dePamphilis C. W. Yi T. S. . (2020). GetOrganelle: a fast and versatile toolkit for accurate *de novo* assembly of organelle genomes. Genome Biol. 21, 241. doi: 10.1186/s13059-020-02154-5, PMID: 32912315 PMC7488116

[B29] Katoh K. Standley D. M. (2013). MAFFT multiple sequence alignment software version 7: Improvements in performance and usability. Mol. Biol. Evol. 30, 772–780. doi: 10.1093/molbev/mst010, PMID: 23329690 PMC3603318

[B30] Kikuchi S. Asakura Y. Imai M. Nakahira Y. Kotani Y. Hashiguchi Y. . (2018). A Ycf2-FtsHi heteromeric AAA-ATPase complex is required for chloroplast protein import. Plant Cell 30, 2677–2703. doi: 10.1105/tpc.18.00357, PMID: 30309901 PMC6305978

[B31] Kim D. Paggi J. M. Park C. Bennett C. Salzberg S. L. (2019). Graph-based genome alignment and genotyping with HISAT2 and HISAT-genotype. Nat. Biotechnol. 37, 907–915. doi: 10.1038/s41587-019-0201-4, PMID: 31375807 PMC7605509

[B32] Kumar S. Stecher G. Tamura K. (2016). MEGA7: Molecular evolutionary genetics analysis version 7.0 for bigger datasets. Mol. Biol. Evol. 33, 1870–1874. doi: 10.1093/molbev/msw054, PMID: 27004904 PMC8210823

[B33] Kurtz S. Choudhuri J. V. Ohlebusch E. Schleiermacher C. Stoye J. Giegerich R. (2001). REPuter: the manifold applications of repeat analysis on a genomic scale. Nucleic Acids Res. 29, 4633–4642. doi: 10.1093/nar/29.22.4633, PMID: 11713313 PMC92531

[B34] Lee E. Helt G. A. Reese J. T. Munoz-Torres M. C. Childers C. P. Buels R. M. . (2013). Web Apollo: a web-based genomic annotation editing platform. Genome Biol. 14, R93. doi: 10.1186/gb-2013-14-8-r93, PMID: 24000942 PMC4053811

[B35] Letunic I. Bork P. (2021). Interactive Tree Of Life (iTOL) v5: an online tool for phylogenetic tree display and annotation. Nucleic Acids Res. 49, W293–W296. doi: 10.1093/nar/gkab301, PMID: 33885785 PMC8265157

[B36] Li H. Handsaker B. Wysoker A. Fennell T. Ruan J. Homer N. . (2009). The sequence alignment/map format and SAMtools. Bioinformatics 25, 2078–2079. doi: 10.1093/bioinformatics/btp352, PMID: 19505943 PMC2723002

[B37] Lin Z. Zhou P. Ma X. Deng Y. Liao Z. Li R. (2020). Comparative analysis of chloroplast genomes in Vasconcellea pubescens A.DC. and Carica papaya L. Sci. Rep. 10, 15799. doi: 10.1038/s41598-020-72769-y, PMID: 32978465 PMC7519098

[B38] Meyer E. H. Giegé P. Gelhaye E. Rayapuram N. Ahuja U. Thöny-Meyer L. . (2005). AtCCMH, an essential component of the c-type cytochrome maturation pathway in *Arabidopsis* mitochondria, interacts with apocytochrome c. Proc. Natl. Acad. Sci. U.S.A. 102, 16113–16118. doi: 10.1073/pnas.0503473102, PMID: 16236729 PMC1276046

[B39] Millar A. H. Whelan J. Soole K. L. Day D. A. (2011). Organization and regulation of mitochondrial respiration in plants. Annu. Rev. Plant Biol. 62, 79–104. doi: 10.1146/annurev-arplant-042110-103857, PMID: 21332361

[B40] Morton B. R. (1998). Selection on the codon bias of chloroplast and cyanelle genes in different plant and algal lineages. J. Mol. Evol. 46, 449–459. doi: 10.1007/PL00006325, PMID: 9541540

[B41] Murray E. E. Lotzer J. Eberle M. (1989). Codon usage in plant genes. Nucleic Acids Res. 17, 477–498. doi: 10.1093/nar/17.2.477, PMID: 2644621 PMC331598

[B42] Neuhaus H. E. Emes M. J. (2000). Nonphotosynthetic metabolism in plastids. Annu. Rev. Plant Physiol. Plant Mol. Biol. 51, 111–140. doi: 10.1146/annurev.arplant.51.1.111, PMID: 15012188

[B43] Nguyen L. T. Schmidt H. A. von Haeseler A. Minh B. Q. (2014). IQ-TREE: A fast and effective stochastic algorithm for estimating maximum-likelihood phylogenies. Mol. Biol. Evol. 32, 268–274. doi: 10.1093/molbev/msu300, PMID: 25371430 PMC4271533

[B44] Palmer J. D. (1985). Comparative organization of chloroplast genomes. Annu. Rev. Genet. 19, 325–354. doi: 10.1146/annurev.ge.19.120185.001545, PMID: 3936406

[B45] Plotkin J. B. Kudla G. (2011). Synonymous but not the same: the causes and consequences of codon bias. Nat. Rev. Genet. 12, 32–42. doi: 10.1038/nrg2899, PMID: 21102527 PMC3074964

[B46] Provan J. Powell W. Hollingsworth P. M. (2001). Chloroplast microsatellites: new tools for studies in plant ecology and evolution. Trends Ecol. Evol. 16, 142–147. doi: 10.1016/S0169-5347(00)02097-8, PMID: 11179578

[B47] Raubeson L. A. Jansen R. K. (2005). “ Chloroplast genomes of plants,” in Plant diversity and evolution: genotypic and phenotypic variation in higher plants (Wallingford, Oxfordshire, UK: CABI), 45–68.

[B48] Rice D. W. Saw J. J. Yu Q. Q. Feng Y. Y. Wang W. L. Wang L. L. . (2008). The chloroplast and mitochondrial genomes of papaya. Genome Res. In press. Available online at: https://www.ncbi.nlm.nih.gov/nucleotide/NC_010323.1 (Accessed October 15, 2025).

[B49] Robinson J. T. Thorvaldsdóttir H. Winckler W. Guttman M. Lander E. S. Getz G. . (2011). Integrative genomics viewer. Nat. Biotechnol. 29, 24–26. doi: 10.1038/nbt.1754, PMID: 21221095 PMC3346182

[B50] Rockinger A. Sousa A. Carvalho F. A. Renner S. S. (2016). Chromosome number reduction in the sister clade of *Carica papaya* with concomitant genome size doubling. Am. J. Bot. 103, 1082–1088. doi: 10.3732/ajb.1600134, PMID: 27234227

[B51] Salas-Solano D. Villalobos-Chaves D. (2021). Frugivory and seed predation of *Jacaratia* *spinosa* (Caricaceae) by Sumichrast’s Vesper Rat, *Nyctomys sumichrasti* (Rodentia: Cricetidae). Mammalogy Notes 7, 225. doi: 10.47603/mano.v7n1.225

[B52] Sawicki J. Krawczyk K. Paukszto Ł. Maździarz M. Kurzyński M. Szablińska-Piernik J. . (2024). Nanopore sequencing technology as an emerging tool for diversity studies of plant organellar genomes. Diversity 16, 173. doi: 10.3390/d16030173

[B53] Scheldeman X. Willemen L. Coppens-d’Eeckenbrugge G. Romeijn-Peeters E. Restrepo M. T. Romero Motoche J. . (2007). Distribution, diversity and environmental adaptation of highland papayas (*Vasconcellea* spp.) in tropical and subtropical America. Biodivers. Conserv. 16, 1867–1884. doi: 10.1007/s10531-006-9086-x

[B54] Sloan D. B. Alverson A. J. Chuckalovcak J. P. Wu M. McCauley D. E. Palmer J. D. . (2012). Rapid evolution of enormous, multichromosomal genomes in flowering plant mitochondria with exceptionally high mutation rates. PloS Biol. 10, e1001241. doi: 10.1371/journal.pbio.1001241, PMID: 22272183 PMC3260318

[B55] Sloan D. B. Triant D. A. Wu M. Taylor D. R. (2013). Cytonuclear interactions and relaxed selection accelerate sequence evolution in organelle ribosomes. Mol. Biol. Evol. 31, 673–682. doi: 10.1093/molbev/mst259, PMID: 24336923

[B56] Small I. D. Schallenberg-Rüdinger M. Takenaka M. Mireau H. Ostersetzer-Biran O. (2020). Plant organellar RNA editing: what 30 years of research has revealed. Plant J. 101, 1040–1056. doi: 10.1111/tpj.14578, PMID: 31630458

[B57] Smith D. R. Keeling P. J. (2015). Mitochondrial and plastid genome architecture: Reoccurring themes, but significant differences at the extremes. Proc. Natl. Acad. Sci. U. S. A. 112, 10177–10184. doi: 10.1073/pnas.1422049112, PMID: 25814499 PMC4547224

[B58] Thiel T. Michalek W. Varshney R. Graner A. (2003). Exploiting EST databases for the development and characterization of gene-derived SSR-markers in barley (*Hordeum vulgare L.*). Theor. Appl. Genet. 106, 411–422. doi: 10.1007/s00122-002-1031-0, PMID: 12589540

[B59] Tigist M. Getnet B. Beza K. Endalamaw M. Lulit M. Tamirat D. . (2016). Extraction and purification of papain enzyme from papaya leaf and the phytochemical components of the leaf. Biotechnol. Int. 9, 176–184. doi: 10.20372/NADRE:1547201550.84

[B60] Tiller N. Bock R. (2014). The translational apparatus of plastids and Its role in plant development. Mol. Plant 7, 1105–1120. doi: 10.1093/mp/ssu022, PMID: 24589494 PMC4086613

[B61] Tillich M. Lehwark P. Pellizzer T. Ulbricht-Jones E. S. Fischer A. Bock R. . (2017). GeSeq – versatile and accurate annotation of organelle genomes. Nucleic Acids Res. 45, W6–W11. doi: 10.1093/nar/gkx391, PMID: 28486635 PMC5570176

[B62] Timmis J. N. Ayliffe M. A. Huang C. Y. Martin W. (2004). Endosymbiotic gene transfer: organelle genomes forge eukaryotic chromosomes. Nat. Rev. Genet. 5, 123–135. doi: 10.1038/nrg1271, PMID: 14735123

[B63] Tineo D. Bustamante D. E. Calderon M. S. (2022). Analysis of the complete plastidial genome of the newly highland papaya *Vasconcellea carvalhoae* (Caricaceae) from Peru. Mitochondrial DNA B Resour. 7, 1882–1886. doi: 10.1080/23802359.2022.2135407, PMID: 36325285 PMC9621235

[B64] Unseld M. Marienfeld J. R. Brandt P. Brennicke A. (1997). The mitochondrial genome of *Arabidopsis thaliana* contains 57 genes in 366,924 nucleotides. Nat. Genet. 15, 57–61. doi: 10.1038/ng0197-57, PMID: 8988169

[B65] Vydianathan R. Khurana J. Tyagi A. Khurana P. (2007). An update on chloroplast genome. Plant Syst. Evol. 271, 101–122. doi: 10.1007/s00606-007-0608-0

[B66] Wang J. Kan S. Liao X. Zhou J. Tembrock L. R. Daniell H. . (2024). Plant organellar genomes: much done, much more to do. Trends Plant Sci. 29, 754–769. doi: 10.1016/j.tplants.2023.12.014, PMID: 38220520

[B67] Wang D. Zhang Y. Zhang Z. Zhu J. Yu J. (2010). KaKs_Calculator 2.0: A toolkit incorporating gamma-series methods and sliding window strategies. Genomics Proteomics Bioinform. 8, 77–80. doi: 10.1016/S1672-0229(10)60008-3, PMID: 20451164 PMC5054116

[B68] Warren J. M. Sloan D. B. (2020). Interchangeable parts: The evolutionarily dynamic tRNA population in plant mitochondria. Mitochondrion 52, 144–156. doi: 10.1016/j.mito.2020.03.007, PMID: 32184120

[B69] Wick R. R. Judd L. M. Holt K. E. (2023). Assembling the perfect bacterial genome using Oxford Nanopore and Illumina sequencing. PloS Comput. Biol. 19, e1010905. doi: 10.1371/journal.pcbi.1010905, PMID: 36862631 PMC9980784

[B70] Wick R. R. Schultz M. B. Zobel J. Holt K. E. (2015). Bandage: interactive visualization of *de novo* genome assemblies. Bioinformatics 31, 3350–3352. doi: 10.1093/bioinformatics/btv383, PMID: 26099265 PMC4595904

[B71] Wicke S. Schneeweiss G. M. dePamphilis C. W. Müller K. F. Quandt D. (2011). The evolution of the plastid chromosome in land plants: gene content, gene order, gene function. Plant Mol. Biol. 76, 273–297. doi: 10.1007/s11103-011-9762-4, PMID: 21424877 PMC3104136

[B72] Zhang D. Gao F. Jakovlić I. Zou H. Zhang J. Li W. X. . (2020). PhyloSuite: An integrated and scalable desktop platform for streamlined molecular sequence data management and evolutionary phylogenetics studies. Mol. Ecol. Resour. 20, 348–355. doi: 10.1111/1755-0998.13096, PMID: 31599058

[B73] Zhang K. Qu G. Zhang Y. Liu J. (2024). Assembly and comparative analysis of the first complete mitochondrial genome of *Astragalus membranaceus* (Fisch.) Bunge: an invaluable traditional Chinese medicine. BMC Plant Biol. 24, 1055. doi: 10.1186/s12870-024-05780-4, PMID: 39511474 PMC11546474

