# Supplementary material for: Comparative analyses of the organelle genomes in Jacaratia spinosa (Caricaceae)
Source: Front Plant Sci. 2026 Jan 12;16:1710417. doi: 10.3389/fpls.2025.1710417 (PMC12836061; doi:10.3389/fpls.2025.1710417)
Supplement: Supplementary file 1 [file DataSheet1.docx]

Supplementary Material


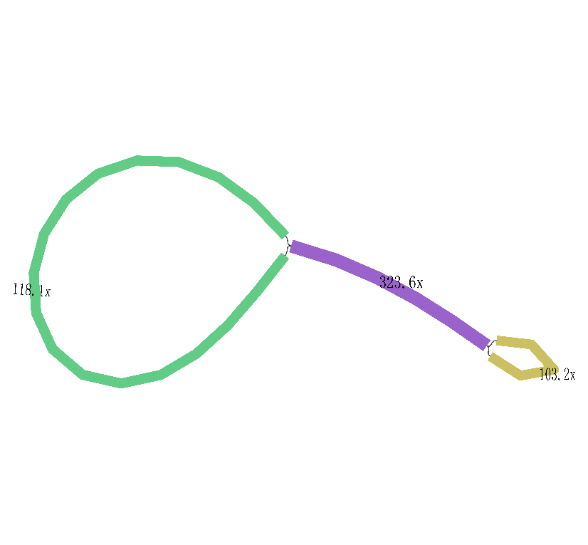

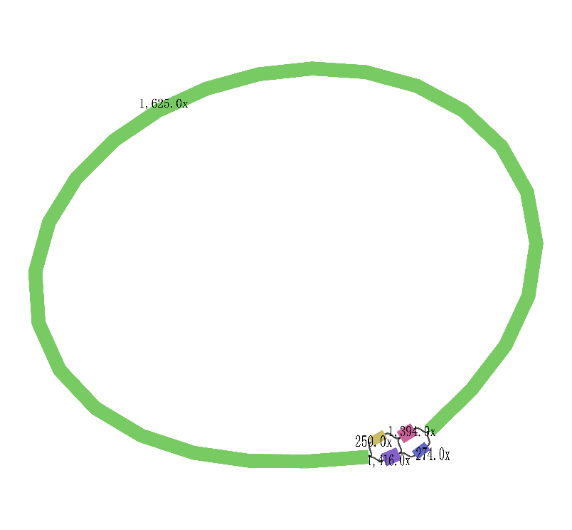


A

B

C


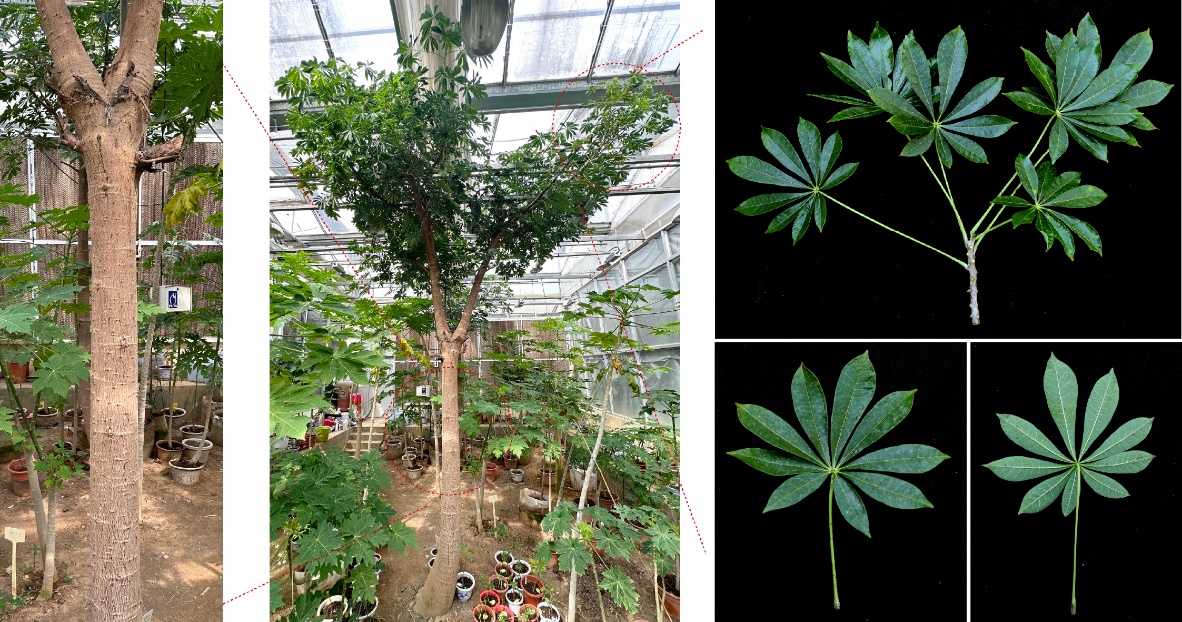


**Supplementary Figure 1**. **Plant morphology and organelle genome structures of *J. spinosa*.** (A) Growth phenotype of the plant. (B) Mitogenome structures. (C) Chloroplast Genome structures.

**Supplementary Figure 2. Multiple sequence alignment of *ycf1*, *rpoC2*, and *rps19* from *C. papaya*, *J. spinosa*, *V. cundinamarcensis*, and *V. carvalhoae*.
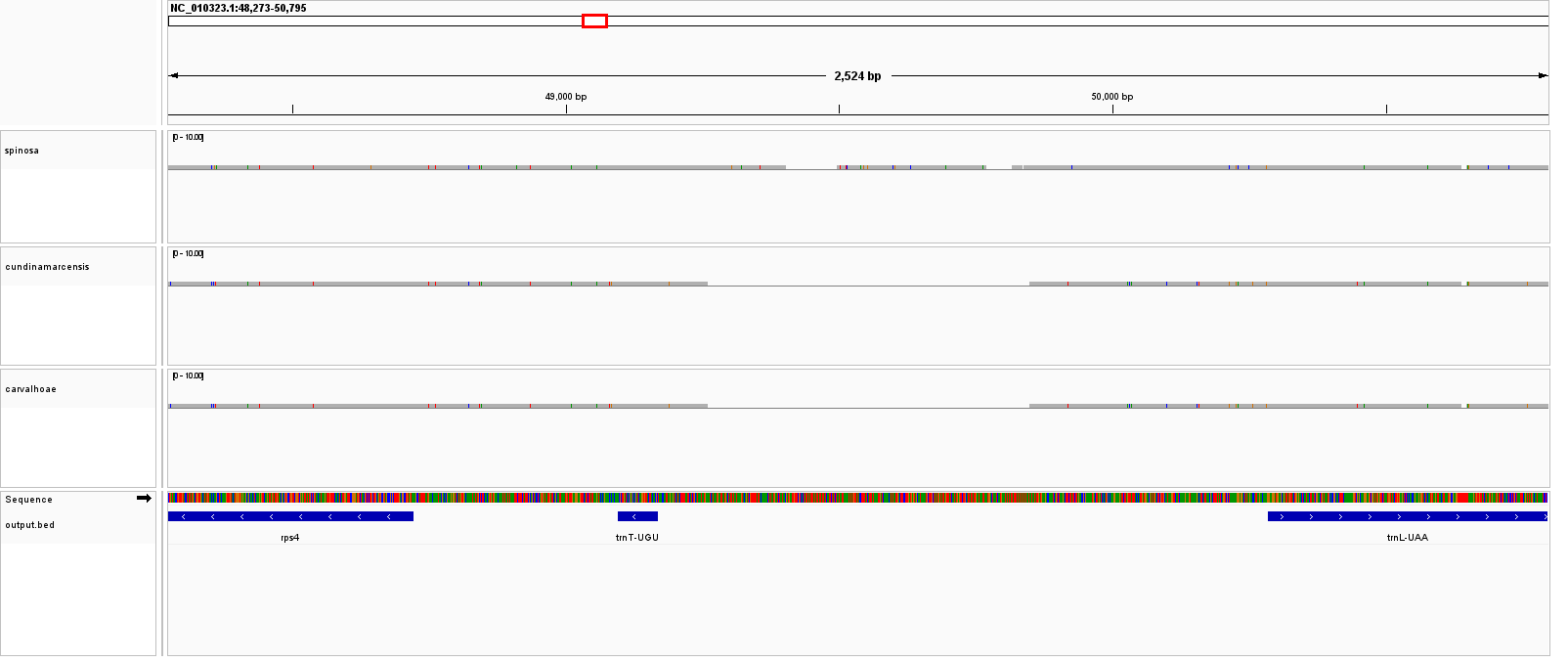

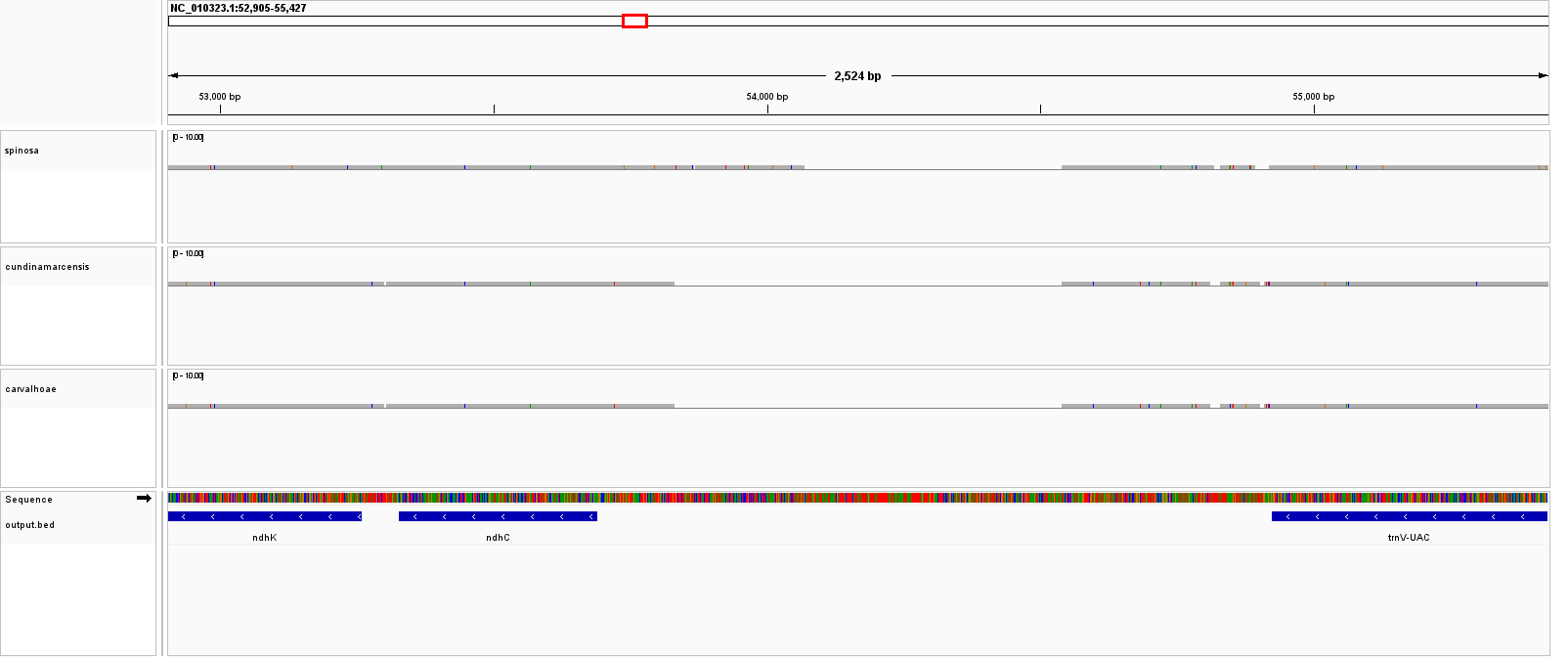
**

**
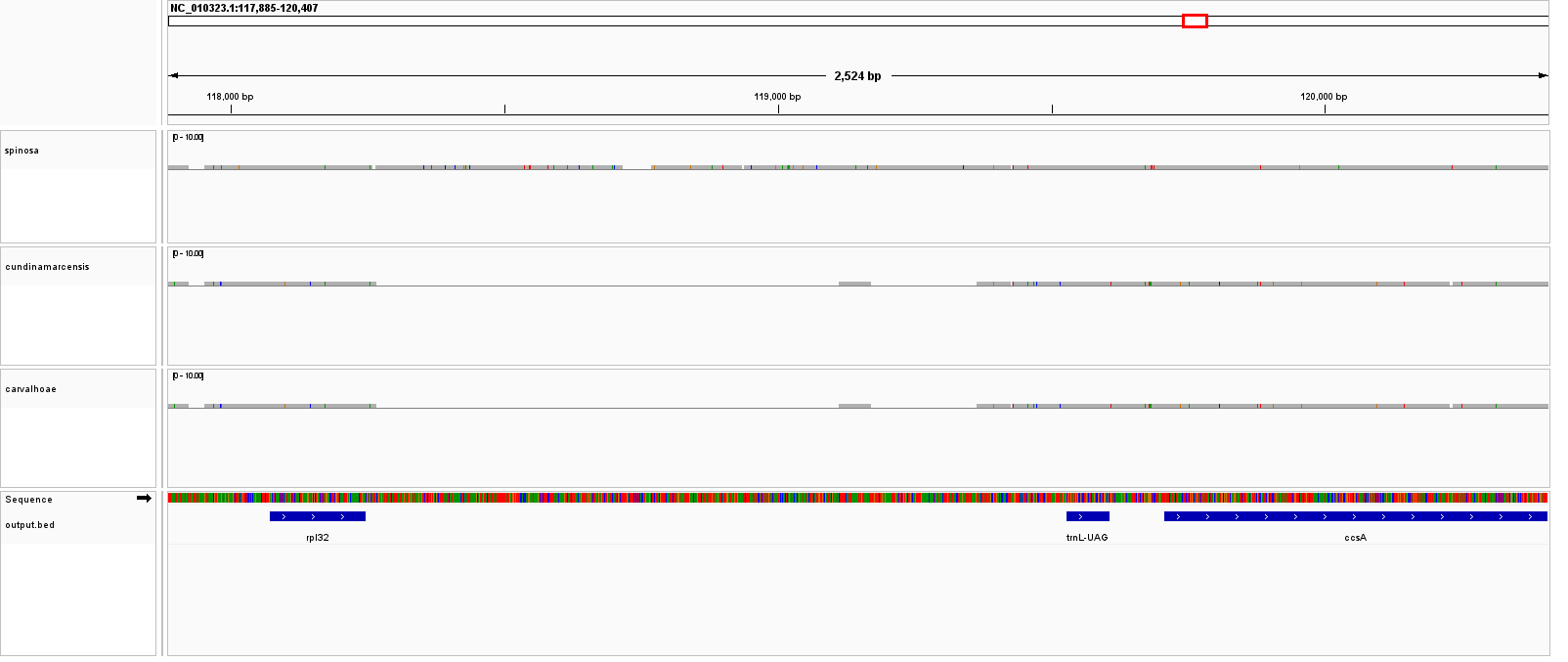
**

**Supplementary Figure 3. IGV visualization of structural variations within conserved noncoding sequences (CNS) in the chloroplast genomes of *J. spinosa*, *V. cundinamarcensis*, *V. carvalhoae*, and *C. papaya*.**

| **Table S1: Total number of perfect Simple Sequence Repeats (SSRs) identified within the mitochondrion genome of *J. spinosa.*** | | | | | | | | | | | | | | |
| --- | --- | --- | --- | --- | --- | --- | --- | --- | --- | --- | --- | --- | --- | --- |
| **Repeats** | **3** | **4** | **5** | **6** | **7** | **8** | **9** | **10** | **11** | **12** | **13** | **14** | **15** | **total** |
| A/T | - | - | - | - | - | - | - | 32 | 15 | 9 | 2 | 4 | 1 | 63 |
| C/G | - | - | - | - | - | - | - |  | 4 |  |  |  |  | 4 |
| AC/GT | - | - |  | 1 |  |  |  |  |  |  |  |  |  | 1 |
| AG/CT | - | - | 11 | 2 |  |  |  |  |  |  |  |  |  | 13 |
| AT/AT | - | - | 5 | 9 | 3 | 1 |  | 1 |  |  | 1 | 1 |  | 21 |
| AAC/GTT | - | 2 |  |  |  |  |  |  |  |  |  |  |  | 2 |
| AAG/CTT | - | 7 |  |  |  |  |  |  |  |  |  |  |  | 7 |
| AAT/ATT | - | 1 |  |  |  |  |  |  |  |  |  |  |  | 1 |
| ACT/AGT | - | 1 |  |  |  |  |  |  |  |  |  |  |  | 1 |
| AGG/CCT | - | 1 |  |  |  |  |  |  |  |  |  |  |  | 1 |
| AAAG/CTTT | 20 |  |  |  |  |  |  |  |  |  |  |  |  | 20 |
| AAAT/ATTT | 1 |  |  |  |  |  |  |  |  |  |  |  |  | 1 |
| AACC/GGTT | 2 |  |  |  |  |  |  |  |  |  |  |  |  | 2 |
| AAGC/CTTG | 4 |  |  |  |  |  |  |  |  |  |  |  |  | 4 |
| AAGG/CCTT | 2 | 1 |  |  |  |  |  |  |  |  |  |  |  | 3 |
| AAGT/ACTT | 2 |  |  |  |  |  |  |  |  |  |  |  |  | 2 |
| AATG/ATTC | 7 |  |  |  |  |  |  |  |  |  |  |  |  | 7 |
| AATT/AATT | 1 |  |  |  |  |  |  |  |  |  |  |  |  | 1 |
| ACAT/ATGT | 1 |  |  |  |  |  |  |  |  |  |  |  |  | 1 |
| ACCG/CGGT | 2 |  |  |  |  |  |  |  |  |  |  |  |  | 2 |
| ACTC/AGTG | 2 |  |  |  |  |  |  |  |  |  |  |  |  | 2 |
| ACTG/AGTC | 3 |  |  |  |  |  |  |  |  |  |  |  |  | 3 |
| AGAT/ATCT | 3 |  |  |  |  |  |  |  |  |  |  |  |  | 3 |
| AGCT/AGCT | 1 |  |  |  |  |  |  |  |  |  |  |  |  | 1 |
| ATCC/ATGG | 1 |  |  |  |  |  |  |  |  |  |  |  |  | 1 |
| CCCG/CGGG | 2 |  |  |  |  |  |  |  |  |  |  |  |  | 2 |
| CCGG/CCGG | 1 |  |  |  |  |  |  |  |  |  |  |  |  | 1 |
| AATGT/ACATT | 1 |  |  |  |  |  |  |  |  |  |  |  |  | 1 |
| ACTAG/AGTCT | 2 |  |  |  |  |  |  |  |  |  |  |  |  | 2 |
| AAAGTG/ACTTTC | 1 |  |  |  |  |  |  |  |  |  |  |  |  | 1 |
| ACAGGG/CCCTGT | 1 |  |  |  |  |  |  |  |  |  |  |  |  | 1 |
|  |  |  |  |  |  |  |  |  |  |  |  |  |  | 175 |

| **Table S2 Pattern of Tandem Repeat distribution in the mitochondrial genome of *J. spinosa.*** | | | | | | | |
| --- | --- | --- | --- | --- | --- | --- | --- |
| **No.** | **Size** | **Copy Number** | **Percent Matches** | **Start** | **End** | | **Repeat Sequence** |
| 1 | 29 | 2.4 | 86 | 18302 | | 18370 | AGAGGAGCGAAGCAGCTCGAACGAAAGTG |
| 2 | 19 | 3 | 78 | 35819 | | 35870 | ATTCATATATATATATATA |
| 3 | 36 | 2.5 | 94 | 43073 | | 43161 | GCAATAACTGTTCCCAGTACTTTGATTCTTCCTAAA |
| 4 | 17 | 2 | 100 | 78968 | | 79001 | TATAGAACTACTCTATT |
| 5 | 28 | 7.5 | 100 | 100442 | | 100652 | GCTTCGCTCCTCTCCTTTCAGTCGAGTG |
| 6 | 19 | 1.9 | 89 | 137777 | | 137813 | TATCTTCTTTTTTTTTTGA |
| 7 | 28 | 2.5 | 86 | 179784 | | 179853 | GGAGAGGAGCGAAGCAGCTCGAACGAAA |
| 8 | 24 | 2.2 | 90 | 180388 | | 180439 | CTCCTTTCAGTCGAGTGGCTATCG |
| 9 | 28 | 3.6 | 89 | 210402 | | 210504 | TCGAGCTGCTTCGCTCCTCTCCTTTCAG |
| 10 | 57 | 2.2 | 98 | 210381 | | 210504 | CTTCGCTCCTCTCACATTCGTTCGAGCTGCTTCGCTCCTCTCCTTTCAGTCGAGCTG |
| 11 | 28 | 4.4 | 87 | 236366 | | 236434 | CTTCGCTCCTCTCCATTCAGTCGAGCTG |
| 12 | 21 | 3.1 | 81 | 238801 | | 238890 | TTTGTCTAAGTAACTTCTTTT |
| 13 | 18 | 5.2 | 90 | 238813 | | 238889 | TATTGATGATAGTGACGA |
| 14 | 9 | 8.6 | 69 | 241459 | | 241484 | CGATAGTGA |
| 15 | 13 | 2 | 100 | 258736 | | 258798 | TGTGATTCCTGTT |
| 16 | 28 | 2.2 | 100 | 295381 | | 295446 | TCCTCTCCTTTCAGTCGAGCTGCTTCGC |
| 17 | 32 | 2.1 | 97 | 340777 | | 340837 | TGGTTTTTTCATGTTGTCAAAGAGTTGAACAA |
| 18 | 22 | 2.8 | 94 | 364044 | | 364072 | TTCCGAACATCTGGTCATCAGA |
| 19 | 15 | 1.9 | 100 | 382723 | | 382794 | GCTGTTGCACCAGGG |
| 20 | 29 | 2.5 | 88 | 411526 | | 411560 | CTGCTTCGCTCCTCTCACATTCGTTCGAG |
| 21 | 15 | 2.3 | 85 | 457637 | | 457735 | AAGGCTAAGAAGCCA |
| 22 | 29 | 3.4 | 98 | 460064 | | 460144 | AGAGGAGCGAAAGCCACTCGACTGAAAGG |
| 23 | 2 | 14 | 100 | 460889 | | 460923 | TA |
| 24 | 38 | 2.1 | 97 | 459391 | | 459418 | TATTTACTCATGATCTGGCCTGGTCGACCCAATCATGA |
| 25 | 2 | 17.5 | 93 | 210381 | | 210504 | AT |

| **Table S3 Scattered Repeats in the mitochondrial genome of *J. spinosa.*** | | | | | | | |
| --- | --- | --- | --- | --- | --- | --- | --- |
| **Start site of the first part** | **End site of the first part** | **Type** | **Alignment length** | **The starting site of the second part** | **The editing site of the second part** | | **E-value** |
| 289124 | 289453 | P | 330 | 373572 | | 373901 | 6.12E-183 |
| 289152 | 289453 | P | 302 | 373572 | | 373873 | 8.18E-169 |
| 49378 | 49674 | P | 297 | 165991 | | 166287 | 9.25E-169 |
| 23590 | 23799 | F | 210 | 143568 | | 143777 | 4.37E-111 |
| 100441 | 100623 | F | 183 | 100469 | | 100651 | 3.99E-100 |
| 103501 | 103680 | P | 180 | 303899 | | 304078 | 3.70E-93 |
| 338659 | 338814 | P | 156 | 432282 | | 432437 | 7.18E-84 |
| 100441 | 100595 | F | 155 | 100497 | | 100651 | 2.87E-83 |
| 291877 | 292026 | F | 150 | 419897 | | 420046 | 2.94E-80 |
| 23657 | 23814 | F | 158 | 143635 | | 143792 | 7.82E-78 |
| 192669 | 192817 | P | 149 | 227487 | | 227635 | 1.17E-74 |
| 275690 | 275817 | P | 128 | 280638 | | 280765 | 5.18E-67 |
| 100137 | 100263 | F | 127 | 338926 | | 339052 | 2.07E-66 |
| 100441 | 100567 | F | 127 | 100525 | | 100651 | 2.07E-66 |
| 27361 | 27483 | F | 123 | 357869 | | 357991 | 5.30E-64 |
| 192695 | 192817 | P | 123 | 227487 | | 227609 | 1.96E-61 |
| 97425 | 97547 | F | 123 | 180760 | | 180882 | 4.33E-57 |
| 197212 | 197326 | P | 115 | 291827 | | 291941 | 1.20E-56 |
| 409848 | 409962 | F | 115 | 425778 | | 425892 | 1.20E-56 |
| 10584 | 10690 | F | 107 | 304450 | | 304556 | 2.28E-54 |
| 97434 | 97547 | F | 114 | 180769 | | 180882 | 8.06E-54 |
| 97449 | 97559 | F | 111 | 180784 | | 180894 | 5.33E-50 |
| 100441 | 100539 | F | 99 | 100553 | | 100651 | 1.49E-49 |
| 339553 | 339655 | F | 103 | 456026 | | 456128 | 1.80E-49 |
| 192533 | 192632 | P | 100 | 227679 | | 227778 | 1.63E-43 |
| 143841 | 143924 | P | 84 | 343423 | | 343506 | 1.60E-40 |
| 304371 | 304457 | P | 87 | 419984 | | 420070 | 6.53E-40 |
| 193058 | 193147 | F | 90 | 255226 | | 255315 | 1.24E-37 |
| 294084 | 294163 | F | 80 | 449515 | | 449594 | 9.84E-36 |
| 133479 | 133552 | F | 74 | 452174 | | 452247 | 1.68E-34 |
| 3136 | 3212 | P | 77 | 334784 | | 334860 | 6.06E-34 |
| 58722 | 58792 | F | 71 | 197549 | | 197619 | 1.08E-32 |
| 100441 | 100511 | F | 71 | 100581 | | 100651 | 1.08E-32 |
| 97482 | 97559 | F | 78 | 180817 | | 180894 | 1.77E-32 |
| 179781 | 179854 | P | 74 | 339788 | | 339861 | 3.73E-32 |
| 57309 | 57378 | F | 70 | 246504 | | 246573 | 4.30E-32 |
| 290568 | 290637 | F | 70 | 339786 | | 339855 | 4.30E-32 |
| 18301 | 18368 | F | 68 | 179785 | | 179852 | 6.88E-31 |
| 100418 | 100483 | P | 66 | 179783 | | 179848 | 1.10E-29 |
| 210438 | 210503 | F | 66 | 382727 | | 382792 | 1.10E-29 |
| 457636 | 457701 | F | 66 | 457665 | | 457730 | 1.10E-29 |
| 100419 | 100483 | F | 65 | 339795 | | 339859 | 4.40E-29 |
| 138455 | 138519 | P | 65 | 312285 | | 312349 | 4.40E-29 |
| 197212 | 197276 | P | 65 | 419897 | | 419961 | 4.40E-29 |
| 18301 | 18368 | P | 68 | 339790 | | 339857 | 1.40E-28 |
| 179787 | 179854 | P | 68 | 290570 | | 290637 | 1.40E-28 |
| 18301 | 18364 | P | 64 | 100418 | | 100481 | 1.76E-28 |
| 210380 | 210446 | F | 67 | 210437 | | 210503 | 5.53E-28 |
| 210381 | 210447 | F | 67 | 382727 | | 382793 | 5.53E-28 |
| 18303 | 18368 | P | 66 | 290572 | | 290637 | 2.18E-27 |
| 210381 | 210449 | F | 69 | 339790 | | 339858 | 3.63E-27 |
| 137458 | 137529 | F | 72 | 195087 | | 195158 | 4.33E-27 |
| 100419 | 100479 | F | 61 | 290577 | | 290637 | 1.13E-26 |
| 179786 | 179853 | P | 68 | 382726 | | 382793 | 1.41E-26 |
| 291964 | 292031 | P | 68 | 304390 | | 304457 | 1.41E-26 |
| 18302 | 18368 | P | 67 | 382727 | | 382793 | 5.48E-26 |
| 18303 | 18369 | P | 67 | 210437 | | 210503 | 5.48E-26 |
| 18301 | 18370 | P | 70 | 210379 | | 210448 | 6.36E-26 |
| 98346 | 98415 | F | 70 | 288173 | | 288242 | 6.36E-26 |
| 24086 | 24144 | F | 59 | 143793 | | 143851 | 1.80E-25 |
| 179787 | 179852 | P | 66 | 210438 | | 210503 | 2.13E-25 |
| 210381 | 210446 | F | 66 | 290572 | | 290637 | 2.13E-25 |
| 179784 | 179852 | P | 69 | 210381 | | 210449 | 2.43E-25 |
| 210438 | 210506 | F | 69 | 290572 | | 290640 | 2.43E-25 |
| 136190 | 136247 | P | 58 | 199174 | | 199231 | 7.22E-25 |
| 95469 | 95533 | P | 65 | 351817 | | 351881 | 8.24E-25 |
| 30097 | 30164 | P | 68 | 127865 | | 127932 | 9.31E-25 |
| 97413 | 97480 | F | 68 | 180748 | | 180815 | 9.31E-25 |
| 98356 | 98423 | F | 68 | 288183 | | 288250 | 9.31E-25 |
| 339789 | 339856 | F | 68 | 382726 | | 382793 | 9.31E-25 |
| 58619 | 58679 | F | 61 | 197444 | | 197504 | 2.06E-24 |
| 49300 | 49356 | P | 57 | 339488 | | 339544 | 2.89E-24 |
| 100609 | 100665 | P | 57 | 179769 | | 179825 | 2.89E-24 |
| 100419 | 100482 | F | 64 | 210386 | | 210449 | 3.20E-24 |
| 181243 | 181309 | F | 67 | 332465 | | 332531 | 3.56E-24 |
| 290571 | 290637 | F | 67 | 382726 | | 382792 | 3.56E-24 |
| 291972 | 292031 | P | 60 | 304390 | | 304449 | 8.12E-24 |
| 2338 | 2393 | P | 56 | 331339 | | 331394 | 1.15E-23 |
| 219121 | 219176 | P | 56 | 424211 | | 424266 | 1.15E-23 |
| 100418 | 100480 | F | 63 | 382731 | | 382793 | 1.24E-23 |
| 210438 | 210503 | F | 66 | 339790 | | 339855 | 1.36E-23 |
| 100418 | 100479 | F | 62 | 210442 | | 210503 | 4.80E-23 |
| 380020 | 380081 | P | 62 | 431698 | | 431759 | 4.80E-23 |
| 97776 | 97840 | F | 65 | 287606 | | 287670 | 5.19E-23 |
| 18316 | 18369 | P | 54 | 210437 | | 210490 | 1.85E-22 |
| 179800 | 179853 | P | 54 | 382726 | | 382779 | 1.85E-22 |
| 218167 | 218220 | P | 54 | 331333 | | 331386 | 1.85E-22 |
| 53204 | 53264 | P | 61 | 431170 | | 431230 | 1.86E-22 |
| 218157 | 218220 | P | 64 | 331333 | | 331396 | 1.98E-22 |
| 181927 | 181983 | F | 57 | 334623 | | 334679 | 4.94E-22 |
| 185810 | 185866 | F | 57 | 335183 | | 335239 | 4.94E-22 |
| 30121 | 30180 | P | 60 | 127849 | | 127908 | 7.18E-22 |
| 18316 | 18368 | P | 53 | 382727 | | 382779 | 7.39E-22 |
| 179800 | 179852 | P | 53 | 210438 | | 210490 | 7.39E-22 |
| 18316 | 18370 | P | 55 | 210379 | | 210433 | 7.62E-21 |
| 209143 | 209197 | F | 55 | 431169 | | 431223 | 7.62E-21 |
| 261290 | 261340 | F | 51 | 353401 | | 353451 | 1.18E-20 |
| 206622 | 206675 | P | 54 | 338442 | | 338495 | 2.99E-20 |
| 137473 | 137529 | F | 57 | 195102 | | 195158 | 4.15E-20 |
| 53215 | 53264 | P | 50 | 209144 | | 209193 | 4.73E-20 |
| 98750 | 98799 | F | 50 | 118562 | | 118611 | 4.73E-20 |
| 179800 | 179852 | P | 53 | 210381 | | 210433 | 1.17E-19 |
| 190153 | 190205 | F | 53 | 290561 | | 290613 | 1.17E-19 |
| 185746 | 185804 | F | 59 | 335119 | | 335177 | 1.58E-19 |
| 184074 | 184122 | P | 49 | 417707 | | 417755 | 1.89E-19 |
| 210401 | 210449 | F | 49 | 258749 | | 258797 | 1.89E-19 |
| 118564 | 118615 | F | 52 | 184617 | | 184668 | 4.61E-19 |
| 2346 | 2393 | F | 48 | 218167 | | 218214 | 7.57E-19 |
| 47752 | 47799 | F | 48 | 335284 | | 335331 | 7.57E-19 |
| 10582 | 10632 | P | 51 | 291904 | | 291954 | 1.81E-18 |
| 10582 | 10632 | P | 51 | 419924 | | 419974 | 1.81E-18 |
| 190155 | 190205 | F | 51 | 339781 | | 339831 | 1.81E-18 |
| 192304 | 192354 | P | 51 | 227882 | | 227932 | 1.81E-18 |
| 351757 | 351813 | F | 57 | 397457 | | 397513 | 2.28E-18 |
| 258749 | 258795 | F | 47 | 382747 | | 382793 | 3.03E-18 |
| 53215 | 53264 | P | 50 | 431170 | | 431219 | 7.09E-18 |
| 98752 | 98804 | F | 53 | 184617 | | 184669 | 9.16E-18 |
| 179815 | 179860 | P | 46 | 401855 | | 401900 | 1.21E-17 |
| 210382 | 210427 | P | 46 | 416181 | | 416226 | 1.21E-17 |
| 210458 | 210503 | F | 46 | 258749 | | 258794 | 1.21E-17 |
| 290573 | 290618 | P | 46 | 416181 | | 416226 | 1.21E-17 |
| 339791 | 339836 | P | 46 | 416181 | | 416226 | 1.21E-17 |
| 100437 | 100485 | F | 49 | 432980 | | 433028 | 2.78E-17 |
| 291904 | 291952 | P | 49 | 304450 | | 304498 | 2.78E-17 |
| 304450 | 304498 | P | 49 | 419924 | | 419972 | 2.78E-17 |
| 192605 | 192656 | P | 52 | 227655 | | 227706 | 3.53E-17 |
| 4003 | 4047 | P | 45 | 324905 | | 324949 | 4.84E-17 |
| 100609 | 100653 | F | 45 | 339817 | | 339861 | 4.84E-17 |
| 18320 | 18373 | F | 54 | 226135 | | 226188 | 1.24E-16 |
| 117418 | 117471 | P | 54 | 223216 | | 223269 | 1.24E-16 |
| 117524 | 117567 | P | 44 | 117524 | | 117567 | 1.94E-16 |
| 103634 | 103680 | P | 47 | 303899 | | 303945 | 4.27E-16 |
| 179783 | 179829 | P | 47 | 432980 | | 433026 | 4.27E-16 |
| 179808 | 179854 | F | 47 | 226139 | | 226185 | 4.27E-16 |
| 339813 | 339859 | F | 47 | 432980 | | 433026 | 4.27E-16 |
| 382725 | 382771 | P | 47 | 397145 | | 397191 | 4.27E-16 |
| 43072 | 43124 | F | 53 | 43108 | | 43160 | 4.67E-16 |
| 58526 | 58578 | P | 53 | 357414 | | 357466 | 4.67E-16 |
| 98329 | 98381 | F | 53 | 288156 | | 288208 | 4.67E-16 |
| 192897 | 192949 | P | 53 | 227345 | | 227397 | 4.67E-16 |
| 409359 | 409408 | P | 50 | 457696 | | 457745 | 5.21E-16 |
| 28118 | 28160 | F | 43 | 298496 | | 298538 | 7.75E-16 |
| 48737 | 48779 | P | 43 | 432988 | | 433030 | 7.75E-16 |
| 100441 | 100483 | F | 43 | 100609 | | 100651 | 7.75E-16 |
| 100469 | 100511 | P | 43 | 179783 | | 179825 | 7.75E-16 |
| 100469 | 100511 | F | 43 | 339817 | | 339859 | 7.75E-16 |
| 100497 | 100539 | P | 43 | 179783 | | 179825 | 7.75E-16 |
| 100497 | 100539 | F | 43 | 339817 | | 339859 | 7.75E-16 |
| 100525 | 100567 | P | 43 | 179783 | | 179825 | 7.75E-16 |
| 100525 | 100567 | F | 43 | 339817 | | 339859 | 7.75E-16 |
| 100553 | 100595 | P | 43 | 179783 | | 179825 | 7.75E-16 |
| 100553 | 100595 | F | 43 | 339817 | | 339859 | 7.75E-16 |
| 100581 | 100623 | P | 43 | 179783 | | 179825 | 7.75E-16 |
| 100581 | 100623 | F | 43 | 339817 | | 339859 | 7.75E-16 |
| 106721 | 106763 | P | 43 | 124945 | | 124987 | 7.75E-16 |
| 118466 | 118508 | F | 43 | 211764 | | 211806 | 7.75E-16 |
| 181941 | 181983 | F | 43 | 334637 | | 334679 | 7.75E-16 |
| 339785 | 339827 | F | 43 | 443575 | | 443617 | 7.75E-16 |
| 179808 | 179853 | F | 46 | 397145 | | 397190 | 1.67E-15 |
| 226139 | 226184 | P | 46 | 382726 | | 382771 | 1.67E-15 |
| 276714 | 276759 | P | 46 | 438461 | | 438506 | 1.67E-15 |
| 192908 | 192959 | P | 52 | 227335 | | 227386 | 1.76E-15 |
| 226135 | 226186 | P | 52 | 290569 | | 290620 | 1.76E-15 |
| 226135 | 226186 | P | 52 | 339787 | | 339838 | 1.76E-15 |
| 238812 | 238863 | F | 52 | 238830 | | 238881 | 1.76E-15 |
| 100434 | 100482 | F | 49 | 258749 | | 258797 | 2.00E-15 |
| 179784 | 179832 | P | 49 | 258749 | | 258797 | 2.00E-15 |
| 192584 | 192632 | P | 49 | 227679 | | 227727 | 2.00E-15 |
| 210438 | 210486 | P | 49 | 226135 | | 226183 | 2.00E-15 |
| 258749 | 258797 | F | 49 | 339810 | | 339858 | 2.00E-15 |
| 1073 | 1114 | P | 42 | 401862 | | 401903 | 3.10E-15 |
| 1076 | 1117 | F | 42 | 397152 | | 397193 | 3.10E-15 |
| 18322 | 18363 | F | 42 | 416181 | | 416222 | 3.10E-15 |
| 100419 | 100460 | P | 42 | 416181 | | 416222 | 3.10E-15 |
| 153007 | 153048 | P | 42 | 382724 | | 382765 | 3.10E-15 |
| 169772 | 169813 | P | 42 | 396482 | | 396523 | 3.10E-15 |
| 179806 | 179847 | F | 42 | 416181 | | 416222 | 3.10E-15 |
| 190160 | 190201 | F | 42 | 443576 | | 443617 | 3.10E-15 |
| 190164 | 190205 | F | 42 | 210381 | | 210422 | 3.10E-15 |
| 210443 | 210484 | P | 42 | 416181 | | 416222 | 3.10E-15 |
| 290568 | 290609 | F | 42 | 443576 | | 443617 | 3.10E-15 |
| 382732 | 382773 | P | 42 | 416181 | | 416222 | 3.10E-15 |
| 18301 | 18345 | P | 45 | 432980 | | 433024 | 6.54E-15 |
| 18324 | 18368 | F | 45 | 397145 | | 397189 | 6.54E-15 |
| 100469 | 100513 | F | 45 | 432984 | | 433028 | 6.54E-15 |
| 100497 | 100541 | F | 45 | 432984 | | 433028 | 6.54E-15 |
| 100525 | 100569 | F | 45 | 432984 | | 433028 | 6.54E-15 |
| 100553 | 100597 | F | 45 | 432984 | | 433028 | 6.54E-15 |
| 100581 | 100625 | F | 45 | 432984 | | 433028 | 6.54E-15 |
| 210438 | 210482 | P | 45 | 397145 | | 397189 | 6.54E-15 |
| 18301 | 18348 | P | 48 | 258749 | | 258796 | 7.68E-15 |
| 18301 | 18341 | P | 41 | 100469 | | 100509 | 1.24E-14 |
| 18301 | 18341 | P | 41 | 100497 | | 100537 | 1.24E-14 |
| 18301 | 18341 | P | 41 | 100525 | | 100565 | 1.24E-14 |
| 18301 | 18341 | P | 41 | 100553 | | 100593 | 1.24E-14 |
| 18301 | 18341 | P | 41 | 100581 | | 100621 | 1.24E-14 |
| 18301 | 18341 | P | 41 | 100609 | | 100649 | 1.24E-14 |
| 87350 | 87390 | F | 41 | 396204 | | 396244 | 1.24E-14 |
| 100417 | 100457 | F | 41 | 266483 | | 266523 | 1.24E-14 |
| 153007 | 153047 | F | 41 | 397151 | | 397191 | 1.24E-14 |
| 190165 | 190205 | P | 41 | 416186 | | 416226 | 1.24E-14 |
| 206635 | 206675 | P | 41 | 338442 | | 338482 | 1.24E-14 |
| 245254 | 245294 | P | 41 | 457637 | | 457677 | 1.24E-14 |
| 245254 | 245294 | P | 41 | 457666 | | 457706 | 1.24E-14 |
| 281289 | 281329 | P | 41 | 394352 | | 394392 | 1.24E-14 |
| 31163 | 31212 | P | 50 | 126821 | | 126870 | 2.50E-14 |
| 192836 | 192885 | P | 50 | 227414 | | 227463 | 2.50E-14 |
| 85873 | 85916 | F | 44 | 203107 | | 203150 | 2.56E-14 |
| 179811 | 179854 | P | 44 | 190162 | | 190205 | 2.56E-14 |
| 100448 | 100494 | F | 47 | 258735 | | 258781 | 2.94E-14 |
| 100476 | 100522 | F | 47 | 258735 | | 258781 | 2.94E-14 |
| 100504 | 100550 | F | 47 | 258735 | | 258781 | 2.94E-14 |
| 100532 | 100578 | F | 47 | 258735 | | 258781 | 2.94E-14 |
| 100560 | 100606 | F | 47 | 258735 | | 258781 | 2.94E-14 |
| 100588 | 100634 | F | 47 | 258735 | | 258781 | 2.94E-14 |
| 1076 | 1115 | F | 40 | 153008 | | 153047 | 4.96E-14 |
| 1076 | 1115 | P | 40 | 382725 | | 382764 | 4.96E-14 |
| 18325 | 18364 | P | 40 | 266484 | | 266523 | 4.96E-14 |
| 87351 | 87390 | F | 40 | 397065 | | 397104 | 4.96E-14 |
| 153007 | 153046 | F | 40 | 179814 | | 179853 | 4.96E-14 |
| 153007 | 153046 | F | 40 | 226145 | | 226184 | 4.96E-14 |
| 179809 | 179848 | P | 40 | 266484 | | 266523 | 4.96E-14 |
| 203107 | 203146 | P | 40 | 245255 | | 245294 | 4.96E-14 |
| 203107 | 203146 | F | 40 | 457637 | | 457676 | 4.96E-14 |
| 203107 | 203146 | F | 40 | 457666 | | 457705 | 4.96E-14 |
| 210442 | 210481 | F | 40 | 266484 | | 266523 | 4.96E-14 |
| 226145 | 226184 | F | 40 | 397151 | | 397190 | 4.96E-14 |
| 226146 | 226185 | P | 40 | 401861 | | 401900 | 4.96E-14 |
| 266484 | 266523 | F | 40 | 382731 | | 382770 | 4.96E-14 |
| 396205 | 396244 | F | 40 | 397065 | | 397104 | 4.96E-14 |
| 24941 | 24989 | F | 49 | 296545 | | 296593 | 9.41E-14 |
| 210381 | 210429 | P | 49 | 226135 | | 226183 | 9.41E-14 |
| 100609 | 100651 | F | 43 | 432984 | | 433026 | 9.99E-14 |
| 190161 | 190203 | P | 43 | 226144 | | 226186 | 9.99E-14 |
| 190163 | 190205 | F | 43 | 382726 | | 382768 | 9.99E-14 |
| 290595 | 290637 | F | 43 | 432980 | | 433022 | 9.99E-14 |
| 18319 | 18364 | F | 46 | 219128 | | 219173 | 1.13E-13 |
| 18319 | 18364 | P | 46 | 424214 | | 424259 | 1.13E-13 |
| 49013 | 49058 | P | 46 | 169480 | | 169525 | 1.13E-13 |
| 85873 | 85918 | P | 46 | 245249 | | 245294 | 1.13E-13 |
| 100418 | 100463 | P | 46 | 219128 | | 219173 | 1.13E-13 |
| 100418 | 100463 | F | 46 | 424214 | | 424259 | 1.13E-13 |
| 179803 | 179848 | F | 46 | 219128 | | 219173 | 1.13E-13 |
| 179803 | 179848 | P | 46 | 424214 | | 424259 | 1.13E-13 |
| 210442 | 210487 | P | 46 | 219128 | | 219173 | 1.13E-13 |
| 210442 | 210487 | F | 46 | 424214 | | 424259 | 1.13E-13 |
| 219128 | 219173 | P | 46 | 382731 | | 382776 | 1.13E-13 |
| 258749 | 258794 | F | 46 | 290592 | | 290637 | 1.13E-13 |
| 290571 | 290616 | P | 46 | 397145 | | 397190 | 1.13E-13 |
| 339789 | 339834 | P | 46 | 397145 | | 397190 | 1.13E-13 |
| 382731 | 382776 | F | 46 | 424214 | | 424259 | 1.13E-13 |
| 1076 | 1114 | F | 39 | 179815 | | 179853 | 1.98E-13 |
| 1076 | 1114 | F | 39 | 226146 | | 226184 | 1.98E-13 |
| 18330 | 18368 | F | 39 | 153007 | | 153045 | 1.98E-13 |
| 99297 | 99335 | F | 39 | 403579 | | 403617 | 1.98E-13 |
| 100469 | 100507 | F | 39 | 290599 | | 290637 | 1.98E-13 |
| 100497 | 100535 | F | 39 | 290599 | | 290637 | 1.98E-13 |
| 100525 | 100563 | F | 39 | 290599 | | 290637 | 1.98E-13 |
| 100553 | 100591 | F | 39 | 290599 | | 290637 | 1.98E-13 |
| 100581 | 100619 | F | 39 | 290599 | | 290637 | 1.98E-13 |
| 100609 | 100647 | F | 39 | 290599 | | 290637 | 1.98E-13 |
| 139489 | 139527 | P | 39 | 382756 | | 382794 | 1.98E-13 |
| 153007 | 153045 | P | 39 | 210438 | | 210476 | 1.98E-13 |
| 153008 | 153046 | P | 39 | 401862 | | 401900 | 1.98E-13 |
| 210386 | 210424 | F | 39 | 266485 | | 266523 | 1.98E-13 |
| 266485 | 266523 | F | 39 | 290577 | | 290615 | 1.98E-13 |
| 266485 | 266523 | F | 39 | 339795 | | 339833 | 1.98E-13 |
| 266485 | 266523 | P | 39 | 416184 | | 416222 | 1.98E-13 |
| 342660 | 342698 | P | 39 | 435479 | | 435517 | 1.98E-13 |
| 382726 | 382764 | F | 39 | 401862 | | 401900 | 1.98E-13 |
| 397152 | 397190 | P | 39 | 401862 | | 401900 | 1.98E-13 |
| 238824 | 238871 | F | 48 | 238842 | | 238889 | 3.53E-13 |
| 18327 | 18368 | P | 42 | 190164 | | 190205 | 3.90E-13 |
| 58687 | 58728 | F | 42 | 197513 | | 197554 | 3.90E-13 |
| 85871 | 85912 | F | 42 | 457664 | | 457705 | 3.90E-13 |
| 190164 | 190205 | F | 42 | 210438 | | 210479 | 3.90E-13 |
| 248172 | 248213 | F | 42 | 251027 | | 251068 | 3.90E-13 |
| 25157 | 25201 | F | 45 | 296763 | | 296807 | 4.31E-13 |
| 100418 | 100462 | P | 45 | 226135 | | 226179 | 4.31E-13 |
| 210381 | 210425 | P | 45 | 397145 | | 397189 | 4.31E-13 |
| 210386 | 210430 | P | 45 | 219128 | | 219172 | 4.31E-13 |
| 210386 | 210430 | F | 45 | 424215 | | 424259 | 4.31E-13 |
| 219128 | 219172 | P | 45 | 290577 | | 290621 | 4.31E-13 |
| 219128 | 219172 | P | 45 | 339795 | | 339839 | 4.31E-13 |
| 290577 | 290621 | F | 45 | 424215 | | 424259 | 4.31E-13 |
| 339795 | 339839 | F | 45 | 424215 | | 424259 | 4.31E-13 |
| 1076 | 1113 | F | 38 | 18331 | | 18368 | 7.93E-13 |
| 1076 | 1113 | P | 38 | 210438 | | 210475 | 7.93E-13 |
| 10595 | 10632 | F | 38 | 197212 | | 197249 | 7.93E-13 |
| 14129 | 14166 | P | 38 | 457636 | | 457673 | 7.93E-13 |
| 14129 | 14166 | P | 38 | 457665 | | 457702 | 7.93E-13 |
| 18331 | 18368 | P | 38 | 401863 | | 401900 | 7.93E-13 |
| 48739 | 48776 | P | 38 | 100448 | | 100485 | 7.93E-13 |
| 48739 | 48776 | P | 38 | 100476 | | 100513 | 7.93E-13 |
| 48739 | 48776 | P | 38 | 100504 | | 100541 | 7.93E-13 |
| 48739 | 48776 | P | 38 | 100532 | | 100569 | 7.93E-13 |
| 48739 | 48776 | P | 38 | 100560 | | 100597 | 7.93E-13 |
| 48739 | 48776 | P | 38 | 100588 | | 100625 | 7.93E-13 |
| 100448 | 100485 | F | 38 | 432991 | | 433028 | 7.93E-13 |
| 139490 | 139527 | P | 38 | 210410 | | 210447 | 7.93E-13 |
| 139490 | 139527 | P | 38 | 258758 | | 258795 | 7.93E-13 |
| 192780 | 192817 | P | 38 | 227487 | | 227524 | 7.93E-13 |
| 197212 | 197249 | F | 38 | 304461 | | 304498 | 7.93E-13 |
| 210381 | 210418 | F | 38 | 443580 | | 443617 | 7.93E-13 |
| 210438 | 210475 | F | 38 | 401863 | | 401900 | 7.93E-13 |
| 245944 | 245981 | P | 38 | 384360 | | 384397 | 7.93E-13 |
| 253358 | 253395 | P | 38 | 324904 | | 324941 | 7.93E-13 |
| 338331 | 338368 | P | 38 | 435502 | | 435539 | 7.93E-13 |
| 342698 | 342735 | P | 38 | 435428 | | 435465 | 7.93E-13 |
| 43025 | 43071 | F | 47 | 43079 | | 43125 | 1.33E-12 |
| 89938 | 89984 | F | 47 | 209308 | | 209354 | 1.33E-12 |
| 99349 | 99395 | F | 47 | 149708 | | 149754 | 1.33E-12 |
| 144389 | 144435 | P | 47 | 180179 | | 180225 | 1.33E-12 |
| 57507 | 57547 | P | 41 | 117369 | | 117409 | 1.52E-12 |
| 85872 | 85912 | F | 41 | 457636 | | 457676 | 1.52E-12 |
| 100418 | 100458 | P | 41 | 397145 | | 397185 | 1.52E-12 |
| 184606 | 184646 | P | 41 | 209142 | | 209182 | 1.52E-12 |
| 226146 | 226186 | P | 41 | 443577 | | 443617 | 1.52E-12 |
| 263949 | 263989 | F | 41 | 306943 | | 306983 | 1.52E-12 |
| 43081 | 43124 | F | 44 | 43117 | | 43160 | 1.65E-12 |
| 226139 | 226182 | F | 44 | 416183 | | 416226 | 1.65E-12 |
| 397145 | 397188 | F | 44 | 416183 | | 416226 | 1.65E-12 |
| 4011 | 4047 | F | 37 | 253358 | | 253394 | 3.17E-12 |
| 14129 | 14165 | P | 37 | 203107 | | 203143 | 3.17E-12 |
| 14129 | 14165 | F | 37 | 245258 | | 245294 | 3.17E-12 |
| 14130 | 14166 | P | 37 | 457694 | | 457730 | 3.17E-12 |
| 28118 | 28154 | F | 37 | 258734 | | 258770 | 3.17E-12 |
| 60192 | 60228 | P | 37 | 118557 | | 118593 | 3.17E-12 |
| 100419 | 100455 | F | 37 | 190169 | | 190205 | 3.17E-12 |
| 139491 | 139527 | P | 37 | 210467 | | 210503 | 3.17E-12 |
| 190169 | 190205 | F | 37 | 266485 | | 266521 | 3.17E-12 |
| 258734 | 258770 | F | 37 | 298496 | | 298532 | 3.17E-12 |
| 264526 | 264562 | F | 37 | 450363 | | 450399 | 3.17E-12 |
| 416190 | 416226 | P | 37 | 443581 | | 443617 | 3.17E-12 |
| 457636 | 457672 | F | 37 | 457694 | | 457730 | 3.17E-12 |
| 460068 | 460104 | F | 37 | 460107 | | 460143 | 3.17E-12 |
| 23236 | 23281 | F | 46 | 444116 | | 444161 | 4.96E-12 |
| 210404 | 210449 | F | 46 | 432980 | | 433025 | 4.96E-12 |
| 258752 | 258797 | F | 46 | 432980 | | 433025 | 4.96E-12 |
| 25133 | 25172 | P | 40 | 72083 | | 72122 | 5.95E-12 |
| 153007 | 153046 | P | 40 | 190163 | | 190202 | 5.95E-12 |
| 153007 | 153046 | P | 40 | 290571 | | 290610 | 5.95E-12 |
| 153007 | 153046 | P | 40 | 339789 | | 339828 | 5.95E-12 |
| 179815 | 179854 | P | 40 | 443578 | | 443617 | 5.95E-12 |
| 190162 | 190201 | F | 40 | 401861 | | 401900 | 5.95E-12 |
| 190163 | 190202 | P | 40 | 397151 | | 397190 | 5.95E-12 |
| 266484 | 266523 | P | 40 | 397146 | | 397185 | 5.95E-12 |
| 290570 | 290609 | F | 40 | 401861 | | 401900 | 5.95E-12 |
| 339788 | 339827 | F | 40 | 401861 | | 401900 | 5.95E-12 |
| 401861 | 401900 | F | 40 | 443578 | | 443617 | 5.95E-12 |
| 117429 | 117471 | P | 43 | 223216 | | 223258 | 6.30E-12 |
| 274098 | 274140 | P | 43 | 339513 | | 339555 | 6.30E-12 |
| 4014 | 4049 | P | 36 | 298495 | | 298530 | 1.27E-11 |
| 18302 | 18337 | F | 36 | 308631 | | 308666 | 1.27E-11 |
| 48741 | 48776 | P | 36 | 100616 | | 100651 | 1.27E-11 |
| 48741 | 48776 | F | 36 | 179783 | | 179818 | 1.27E-11 |
| 48741 | 48776 | P | 36 | 339824 | | 339859 | 1.27E-11 |
| 85871 | 85906 | F | 36 | 457693 | | 457728 | 1.27E-11 |
| 100445 | 100480 | P | 36 | 308631 | | 308666 | 1.27E-11 |
| 100473 | 100508 | P | 36 | 308631 | | 308666 | 1.27E-11 |
| 100501 | 100536 | P | 36 | 308631 | | 308666 | 1.27E-11 |
| 100529 | 100564 | P | 36 | 308631 | | 308666 | 1.27E-11 |
| 100557 | 100592 | P | 36 | 308631 | | 308666 | 1.27E-11 |
| 100585 | 100620 | P | 36 | 308631 | | 308666 | 1.27E-11 |
| 100613 | 100648 | P | 36 | 308631 | | 308666 | 1.27E-11 |
| 179786 | 179821 | F | 36 | 308631 | | 308666 | 1.27E-11 |
| 203107 | 203142 | F | 36 | 457695 | | 457730 | 1.27E-11 |
| 205709 | 205744 | F | 36 | 207518 | | 207553 | 1.27E-11 |
| 226144 | 226179 | P | 36 | 266484 | | 266519 | 1.27E-11 |
| 245259 | 245294 | P | 36 | 457695 | | 457730 | 1.27E-11 |
| 264253 | 264288 | P | 36 | 377182 | | 377217 | 1.27E-11 |
| 308631 | 308666 | P | 36 | 339821 | | 339856 | 1.27E-11 |
| 339824 | 339859 | F | 36 | 432991 | | 433026 | 1.27E-11 |
| 24293 | 24337 | F | 45 | 295881 | | 295925 | 1.86E-11 |
| 127684 | 127728 | P | 45 | 146833 | | 146877 | 1.86E-11 |
| 274043 | 274087 | P | 45 | 330998 | | 331042 | 1.86E-11 |
| 275142 | 275186 | F | 45 | 291337 | | 291381 | 1.86E-11 |
| 1076 | 1114 | P | 39 | 190163 | | 190201 | 2.32E-11 |
| 1076 | 1114 | P | 39 | 290571 | | 290609 | 2.32E-11 |
| 1076 | 1114 | P | 39 | 339789 | | 339827 | 2.32E-11 |
| 1076 | 1114 | P | 39 | 443579 | | 443617 | 2.32E-11 |
| 53226 | 53264 | F | 39 | 184606 | | 184644 | 2.32E-11 |
| 102258 | 102296 | P | 39 | 396248 | | 396286 | 2.32E-11 |
| 153007 | 153045 | P | 39 | 210381 | | 210419 | 2.32E-11 |
| 153008 | 153046 | P | 39 | 443579 | | 443617 | 2.32E-11 |
| 339707 | 339745 | P | 39 | 400908 | | 400946 | 2.32E-11 |
| 382726 | 382764 | F | 39 | 443579 | | 443617 | 2.32E-11 |
| 397152 | 397190 | P | 39 | 443579 | | 443617 | 2.32E-11 |
| 100469 | 100510 | F | 42 | 210408 | | 210449 | 2.40E-11 |
| 100469 | 100510 | F | 42 | 258756 | | 258797 | 2.40E-11 |
| 100497 | 100538 | F | 42 | 210408 | | 210449 | 2.40E-11 |
| 100497 | 100538 | F | 42 | 258756 | | 258797 | 2.40E-11 |
| 100525 | 100566 | F | 42 | 210408 | | 210449 | 2.40E-11 |
| 100525 | 100566 | F | 42 | 258756 | | 258797 | 2.40E-11 |
| 100553 | 100594 | F | 42 | 210408 | | 210449 | 2.40E-11 |
| 100553 | 100594 | F | 42 | 258756 | | 258797 | 2.40E-11 |
| 100581 | 100622 | F | 42 | 210408 | | 210449 | 2.40E-11 |
| 100581 | 100622 | F | 42 | 258756 | | 258797 | 2.40E-11 |
| 100609 | 100650 | F | 42 | 210408 | | 210449 | 2.40E-11 |
| 100609 | 100650 | F | 42 | 258756 | | 258797 | 2.40E-11 |
| 166330 | 166371 | P | 42 | 435478 | | 435519 | 2.40E-11 |
| 219131 | 219172 | F | 42 | 416181 | | 416222 | 2.40E-11 |
| 416181 | 416222 | P | 42 | 424215 | | 424256 | 2.40E-11 |
| 4014 | 4048 | P | 35 | 28118 | | 28152 | 5.08E-11 |
| 4014 | 4048 | P | 35 | 258734 | | 258768 | 5.08E-11 |
| 14122 | 14156 | P | 35 | 308639 | | 308673 | 5.08E-11 |
| 14132 | 14166 | P | 35 | 85872 | | 85906 | 5.08E-11 |
| 28119 | 28153 | F | 35 | 210415 | | 210449 | 5.08E-11 |
| 28119 | 28153 | F | 35 | 258763 | | 258797 | 5.08E-11 |
| 30300 | 30334 | F | 35 | 146836 | | 146870 | 5.08E-11 |
| 53230 | 53264 | P | 35 | 431170 | | 431204 | 5.08E-11 |
| 100418 | 100452 | P | 35 | 153007 | | 153041 | 5.08E-11 |
| 153007 | 153041 | P | 35 | 266484 | | 266518 | 5.08E-11 |
| 190169 | 190203 | P | 35 | 226144 | | 226178 | 5.08E-11 |
| 210414 | 210448 | P | 35 | 253361 | | 253395 | 5.08E-11 |
| 210414 | 210448 | F | 35 | 324904 | | 324938 | 5.08E-11 |
| 210415 | 210449 | F | 35 | 258735 | | 258769 | 5.08E-11 |
| 210415 | 210449 | F | 35 | 298497 | | 298531 | 5.08E-11 |
| 253361 | 253395 | P | 35 | 258762 | | 258796 | 5.08E-11 |
| 258735 | 258769 | F | 35 | 258763 | | 258797 | 5.08E-11 |
| 258762 | 258796 | F | 35 | 324904 | | 324938 | 5.08E-11 |
| 258763 | 258797 | F | 35 | 298497 | | 298531 | 5.08E-11 |
| 290603 | 290637 | P | 35 | 308632 | | 308666 | 5.08E-11 |
| 54522 | 54565 | P | 44 | 266476 | | 266519 | 6.93E-11 |
| 382750 | 382793 | F | 44 | 432980 | | 433023 | 6.93E-11 |
| 1076 | 1113 | P | 38 | 210381 | | 210418 | 9.04E-11 |
| 18331 | 18368 | P | 38 | 443580 | | 443617 | 9.04E-11 |
| 49618 | 49655 | F | 38 | 357852 | | 357889 | 9.04E-11 |
| 166010 | 166047 | P | 38 | 357852 | | 357889 | 9.04E-11 |
| 192502 | 192539 | P | 38 | 227771 | | 227808 | 9.04E-11 |
| 210381 | 210418 | F | 38 | 401863 | | 401900 | 9.04E-11 |
| 210438 | 210475 | F | 38 | 443580 | | 443617 | 9.04E-11 |
| 54517 | 54557 | F | 41 | 226139 | | 226179 | 9.15E-11 |
| 99359 | 99399 | F | 41 | 149718 | | 149758 | 9.15E-11 |
| 1076 | 1109 | P | 34 | 100418 | | 100451 | 2.03E-10 |
| 1076 | 1109 | P | 34 | 266484 | | 266517 | 2.03E-10 |
| 4014 | 4047 | P | 34 | 210415 | | 210448 | 2.03E-10 |
| 4014 | 4047 | P | 34 | 258763 | | 258796 | 2.03E-10 |
| 18301 | 18334 | F | 34 | 48743 | | 48776 | 2.03E-10 |
| 28119 | 28152 | P | 34 | 253361 | | 253394 | 2.03E-10 |
| 28119 | 28152 | F | 34 | 324905 | | 324938 | 2.03E-10 |
| 65859 | 65892 | F | 34 | 219139 | | 219172 | 2.03E-10 |
| 65859 | 65892 | P | 34 | 424215 | | 424248 | 2.03E-10 |
| 100418 | 100451 | F | 34 | 401867 | | 401900 | 2.03E-10 |
| 136156 | 136189 | P | 34 | 396283 | | 396316 | 2.03E-10 |
| 139490 | 139523 | F | 34 | 253362 | | 253395 | 2.03E-10 |
| 139490 | 139523 | P | 34 | 324904 | | 324937 | 2.03E-10 |
| 153007 | 153040 | F | 34 | 416189 | | 416222 | 2.03E-10 |
| 253361 | 253394 | P | 34 | 258735 | | 258768 | 2.03E-10 |
| 253361 | 253394 | P | 34 | 298497 | | 298530 | 2.03E-10 |
| 253362 | 253395 | P | 34 | 382760 | | 382793 | 2.03E-10 |
| 258735 | 258768 | F | 34 | 324905 | | 324938 | 2.03E-10 |
| 266484 | 266517 | F | 34 | 401867 | | 401900 | 2.03E-10 |
| 274107 | 274140 | P | 34 | 339513 | | 339546 | 2.03E-10 |
| 298497 | 298530 | F | 34 | 324905 | | 324938 | 2.03E-10 |
| 324904 | 324937 | F | 34 | 382760 | | 382793 | 2.03E-10 |
| 54516 | 54558 | P | 43 | 100417 | | 100459 | 2.58E-10 |
| 58550 | 58592 | P | 43 | 357400 | | 357442 | 2.58E-10 |
| 210461 | 210503 | F | 43 | 432980 | | 433022 | 2.58E-10 |
| 100469 | 100508 | F | 40 | 382754 | | 382793 | 3.48E-10 |
| 100497 | 100536 | F | 40 | 382754 | | 382793 | 3.48E-10 |
| 100525 | 100564 | F | 40 | 382754 | | 382793 | 3.48E-10 |
| 100553 | 100592 | F | 40 | 382754 | | 382793 | 3.48E-10 |
| 100581 | 100620 | F | 40 | 382754 | | 382793 | 3.48E-10 |
| 100609 | 100648 | F | 40 | 382754 | | 382793 | 3.48E-10 |
| 107755 | 107794 | P | 40 | 112310 | | 112349 | 3.48E-10 |
| 133003 | 133042 | P | 40 | 431694 | | 431733 | 3.48E-10 |
| 185316 | 185355 | F | 40 | 433455 | | 433494 | 3.48E-10 |
| 219134 | 219173 | P | 40 | 266484 | | 266523 | 3.48E-10 |
| 264868 | 264907 | F | 40 | 456694 | | 456733 | 3.48E-10 |
| 266484 | 266523 | F | 40 | 424214 | | 424253 | 3.48E-10 |
| 1076 | 1108 | F | 33 | 416190 | | 416222 | 8.12E-10 |
| 4015 | 4047 | F | 33 | 139490 | | 139522 | 8.12E-10 |
| 4015 | 4047 | P | 33 | 382761 | | 382793 | 8.12E-10 |
| 18316 | 18348 | P | 33 | 258749 | | 258781 | 8.12E-10 |
| 28119 | 28151 | P | 33 | 139490 | | 139522 | 8.12E-10 |
| 28119 | 28151 | F | 33 | 382761 | | 382793 | 8.12E-10 |
| 48744 | 48776 | F | 33 | 308631 | | 308663 | 8.12E-10 |
| 59093 | 59125 | F | 33 | 101468 | | 101500 | 8.12E-10 |
| 60189 | 60221 | F | 33 | 209139 | | 209171 | 8.12E-10 |
| 83572 | 83604 | P | 33 | 446214 | | 446246 | 8.12E-10 |
| 98383 | 98415 | F | 33 | 288210 | | 288242 | 8.12E-10 |
| 100419 | 100451 | F | 33 | 443585 | | 443617 | 8.12E-10 |
| 104357 | 104389 | P | 33 | 248124 | | 248156 | 8.12E-10 |
| 105741 | 105773 | P | 33 | 373400 | | 373432 | 8.12E-10 |
| 139490 | 139522 | P | 33 | 258735 | | 258767 | 8.12E-10 |
| 139490 | 139522 | P | 33 | 298497 | | 298529 | 8.12E-10 |
| 179800 | 179832 | P | 33 | 258749 | | 258781 | 8.12E-10 |
| 184613 | 184645 | P | 33 | 431169 | | 431201 | 8.12E-10 |
| 210471 | 210503 | P | 33 | 253363 | | 253395 | 8.12E-10 |
| 210471 | 210503 | F | 33 | 324904 | | 324936 | 8.12E-10 |
| 258735 | 258767 | F | 33 | 382761 | | 382793 | 8.12E-10 |
| 266485 | 266517 | F | 33 | 443585 | | 443617 | 8.12E-10 |
| 298497 | 298529 | F | 33 | 382761 | | 382793 | 8.12E-10 |
| 308631 | 308663 | P | 33 | 432991 | | 433023 | 8.12E-10 |
| 394839 | 394871 | P | 33 | 396073 | | 396105 | 8.12E-10 |
| 401868 | 401900 | P | 33 | 416190 | | 416222 | 8.12E-10 |
| 18323 | 18364 | F | 42 | 54516 | | 54557 | 9.61E-10 |
| 54516 | 54557 | F | 42 | 179807 | | 179848 | 9.61E-10 |
| 54516 | 54557 | P | 42 | 210442 | | 210483 | 9.61E-10 |
| 54516 | 54557 | P | 42 | 382731 | | 382772 | 9.61E-10 |
| 66434 | 66475 | F | 42 | 393859 | | 393900 | 9.61E-10 |
| 238812 | 238853 | F | 42 | 238848 | | 238889 | 9.61E-10 |
| 27351 | 27389 | P | 39 | 280427 | | 280465 | 1.32E-09 |
| 28119 | 28157 | F | 39 | 100616 | | 100654 | 1.32E-09 |
| 28119 | 28157 | P | 39 | 179780 | | 179818 | 1.32E-09 |
| 48739 | 48777 | P | 39 | 258734 | | 258772 | 1.32E-09 |
| 100469 | 100507 | F | 39 | 210465 | | 210503 | 1.32E-09 |
| 100497 | 100535 | F | 39 | 210465 | | 210503 | 1.32E-09 |
| 100525 | 100563 | F | 39 | 210465 | | 210503 | 1.32E-09 |
| 100553 | 100591 | F | 39 | 210465 | | 210503 | 1.32E-09 |
| 100581 | 100619 | F | 39 | 210465 | | 210503 | 1.32E-09 |
| 100609 | 100647 | F | 39 | 210465 | | 210503 | 1.32E-09 |
| 100616 | 100654 | F | 39 | 298497 | | 298535 | 1.32E-09 |
| 166332 | 166370 | F | 39 | 342660 | | 342698 | 1.32E-09 |
| 179780 | 179818 | P | 39 | 298497 | | 298535 | 1.32E-09 |
| 258734 | 258772 | F | 39 | 432990 | | 433028 | 1.32E-09 |
| 340776 | 340814 | F | 39 | 340798 | | 340836 | 1.32E-09 |
| 29319 | 29354 | F | 36 | 145862 | | 145897 | 1.37E-09 |
| 87443 | 87478 | P | 36 | 199719 | | 199754 | 1.37E-09 |
| 138483 | 138518 | F | 36 | 203106 | | 203141 | 1.37E-09 |
| 192850 | 192885 | P | 36 | 227414 | | 227449 | 1.37E-09 |
| 203106 | 203141 | P | 36 | 312286 | | 312321 | 1.37E-09 |
| 230201 | 230236 | P | 36 | 337316 | | 337351 | 1.37E-09 |
| 4016 | 4047 | P | 32 | 210472 | | 210503 | 3.25E-09 |
| 25491 | 25522 | P | 32 | 98702 | | 98733 | 3.25E-09 |
| 28119 | 28150 | F | 32 | 210472 | | 210503 | 3.25E-09 |
| 48745 | 48776 | P | 32 | 290606 | | 290637 | 3.25E-09 |
| 49300 | 49331 | F | 32 | 274109 | | 274140 | 3.25E-09 |
| 60192 | 60223 | P | 32 | 98750 | | 98781 | 3.25E-09 |
| 100637 | 100668 | F | 32 | 124169 | | 124200 | 3.25E-09 |
| 118724 | 118755 | F | 32 | 444889 | | 444920 | 3.25E-09 |
| 138485 | 138516 | P | 32 | 180384 | | 180415 | 3.25E-09 |
| 180384 | 180415 | F | 32 | 312288 | | 312319 | 3.25E-09 |
| 184673 | 184704 | F | 32 | 231083 | | 231114 | 3.25E-09 |
| 210472 | 210503 | F | 32 | 258735 | | 258766 | 3.25E-09 |
| 210472 | 210503 | F | 32 | 298497 | | 298528 | 3.25E-09 |
| 233652 | 233683 | P | 32 | 344314 | | 344345 | 3.25E-09 |
| 290606 | 290637 | F | 32 | 432991 | | 433022 | 3.25E-09 |
| 337064 | 337095 | F | 32 | 339846 | | 339877 | 3.25E-09 |
| 420683 | 420714 | P | 32 | 420683 | | 420714 | 3.25E-09 |
| 460894 | 460925 | P | 32 | 460894 | | 460925 | 3.25E-09 |
| 30954 | 30994 | P | 41 | 127038 | | 127078 | 3.57E-09 |
| 54516 | 54556 | P | 41 | 210386 | | 210426 | 3.57E-09 |
| 54516 | 54556 | P | 41 | 290577 | | 290617 | 3.57E-09 |
| 54516 | 54556 | P | 41 | 339795 | | 339835 | 3.57E-09 |
| 54516 | 54556 | F | 41 | 416182 | | 416222 | 3.57E-09 |
| 180525 | 180565 | F | 41 | 184806 | | 184846 | 3.57E-09 |
| 219133 | 219173 | F | 41 | 226139 | | 226179 | 3.57E-09 |
| 219133 | 219173 | F | 41 | 397145 | | 397185 | 3.57E-09 |
| 226139 | 226179 | P | 41 | 424214 | | 424254 | 3.57E-09 |
| 362418 | 362458 | F | 41 | 364648 | | 364688 | 3.57E-09 |
| 397145 | 397185 | P | 41 | 424214 | | 424254 | 3.57E-09 |
| 18302 | 18339 | F | 38 | 139490 | | 139527 | 5.02E-09 |
| 28119 | 28156 | F | 38 | 339824 | | 339861 | 5.02E-09 |
| 65859 | 65896 | P | 38 | 190165 | | 190202 | 5.02E-09 |
| 65859 | 65896 | P | 38 | 210382 | | 210419 | 5.02E-09 |
| 65859 | 65896 | P | 38 | 290573 | | 290610 | 5.02E-09 |
| 65859 | 65896 | P | 38 | 339791 | | 339828 | 5.02E-09 |
| 65859 | 65896 | F | 38 | 416189 | | 416226 | 5.02E-09 |
| 100443 | 100480 | P | 38 | 139490 | | 139527 | 5.02E-09 |
| 100471 | 100508 | P | 38 | 139490 | | 139527 | 5.02E-09 |
| 100499 | 100536 | P | 38 | 139490 | | 139527 | 5.02E-09 |
| 100527 | 100564 | P | 38 | 139490 | | 139527 | 5.02E-09 |
| 100555 | 100592 | P | 38 | 139490 | | 139527 | 5.02E-09 |
| 100583 | 100620 | P | 38 | 139490 | | 139527 | 5.02E-09 |
| 100611 | 100648 | P | 38 | 139490 | | 139527 | 5.02E-09 |
| 139490 | 139527 | F | 38 | 179786 | | 179823 | 5.02E-09 |
| 139490 | 139527 | P | 38 | 339819 | | 339856 | 5.02E-09 |
| 298497 | 298534 | F | 38 | 339824 | | 339861 | 5.02E-09 |
| 7749 | 7783 | P | 35 | 376334 | | 376368 | 5.33E-09 |
| 14131 | 14165 | P | 35 | 138484 | | 138518 | 5.33E-09 |
| 14131 | 14165 | F | 35 | 312286 | | 312320 | 5.33E-09 |
| 49024 | 49058 | P | 35 | 169480 | | 169514 | 5.33E-09 |
| 58582 | 58616 | P | 35 | 357376 | | 357410 | 5.33E-09 |
| 138484 | 138518 | P | 35 | 245260 | | 245294 | 5.33E-09 |
| 138484 | 138518 | F | 35 | 457637 | | 457671 | 5.33E-09 |
| 138484 | 138518 | F | 35 | 457666 | | 457700 | 5.33E-09 |
| 138484 | 138518 | F | 35 | 457695 | | 457729 | 5.33E-09 |
| 245260 | 245294 | F | 35 | 312286 | | 312320 | 5.33E-09 |
| 312286 | 312320 | P | 35 | 457637 | | 457671 | 5.33E-09 |
| 312286 | 312320 | P | 35 | 457666 | | 457700 | 5.33E-09 |
| 312286 | 312320 | P | 35 | 457695 | | 457729 | 5.33E-09 |
| 180411 | 180441 | F | 31 | 382765 | | 382795 | 1.30E-08 |
| 245947 | 245977 | P | 31 | 266519 | | 266549 | 1.30E-08 |
| 266519 | 266549 | F | 31 | 384364 | | 384394 | 1.30E-08 |
| 335970 | 336000 | P | 31 | 357485 | | 357515 | 1.30E-08 |
| 274030 | 274069 | P | 40 | 331016 | | 331055 | 1.32E-08 |
| 28118 | 28154 | P | 37 | 48741 | | 48777 | 1.90E-08 |
| 28118 | 28154 | F | 37 | 432990 | | 433026 | 1.90E-08 |
| 48741 | 48777 | P | 37 | 298496 | | 298532 | 1.90E-08 |
| 65860 | 65896 | P | 37 | 443581 | | 443617 | 1.90E-08 |
| 139491 | 139527 | P | 37 | 290601 | | 290637 | 1.90E-08 |
| 190169 | 190205 | P | 37 | 219136 | | 219172 | 1.90E-08 |
| 190169 | 190205 | F | 37 | 424215 | | 424251 | 1.90E-08 |
| 298496 | 298532 | F | 37 | 432990 | | 433026 | 1.90E-08 |
| 460887 | 460923 | R | 37 | 460887 | | 460923 | 1.90E-08 |
| 85873 | 85906 | F | 34 | 138484 | | 138517 | 2.07E-08 |
| 85873 | 85906 | P | 34 | 312287 | | 312320 | 2.07E-08 |
| 138485 | 138518 | P | 34 | 409375 | | 409408 | 2.07E-08 |
| 295380 | 295413 | F | 34 | 295412 | | 295445 | 2.07E-08 |
| 312286 | 312319 | F | 34 | 409375 | | 409408 | 2.07E-08 |
| 378090 | 378123 | F | 34 | 448399 | | 448432 | 2.07E-08 |
| 291820 | 291858 | F | 39 | 419838 | | 419876 | 4.89E-08 |
| 6620 | 6649 | P | 30 | 100638 | | 100667 | 5.20E-08 |
| 6620 | 6649 | P | 30 | 124170 | | 124199 | 5.20E-08 |
| 18310 | 18339 | F | 30 | 203116 | | 203145 | 5.20E-08 |
| 18310 | 18339 | P | 30 | 245256 | | 245285 | 5.20E-08 |
| 18310 | 18339 | F | 30 | 457646 | | 457675 | 5.20E-08 |
| 18310 | 18339 | F | 30 | 457675 | | 457704 | 5.20E-08 |
| 60192 | 60221 | P | 30 | 184617 | | 184646 | 5.20E-08 |
| 85882 | 85911 | P | 30 | 432986 | | 433015 | 5.20E-08 |
| 98752 | 98781 | P | 30 | 209142 | | 209171 | 5.20E-08 |
| 100443 | 100472 | P | 30 | 203116 | | 203145 | 5.20E-08 |
| 100443 | 100472 | F | 30 | 245256 | | 245285 | 5.20E-08 |
| 100443 | 100472 | P | 30 | 457646 | | 457675 | 5.20E-08 |
| 100443 | 100472 | P | 30 | 457675 | | 457704 | 5.20E-08 |
| 100471 | 100500 | P | 30 | 203116 | | 203145 | 5.20E-08 |
| 100471 | 100500 | F | 30 | 245256 | | 245285 | 5.20E-08 |
| 100471 | 100500 | P | 30 | 457646 | | 457675 | 5.20E-08 |
| 100471 | 100500 | P | 30 | 457675 | | 457704 | 5.20E-08 |
| 100499 | 100528 | P | 30 | 203116 | | 203145 | 5.20E-08 |
| 100499 | 100528 | F | 30 | 245256 | | 245285 | 5.20E-08 |
| 100499 | 100528 | P | 30 | 457646 | | 457675 | 5.20E-08 |
| 100499 | 100528 | P | 30 | 457675 | | 457704 | 5.20E-08 |
| 100527 | 100556 | P | 30 | 203116 | | 203145 | 5.20E-08 |
| 100527 | 100556 | F | 30 | 245256 | | 245285 | 5.20E-08 |
| 100527 | 100556 | P | 30 | 457646 | | 457675 | 5.20E-08 |
| 100527 | 100556 | P | 30 | 457675 | | 457704 | 5.20E-08 |
| 100555 | 100584 | P | 30 | 203116 | | 203145 | 5.20E-08 |
| 100555 | 100584 | F | 30 | 245256 | | 245285 | 5.20E-08 |
| 100555 | 100584 | P | 30 | 457646 | | 457675 | 5.20E-08 |
| 100555 | 100584 | P | 30 | 457675 | | 457704 | 5.20E-08 |
| 100583 | 100612 | P | 30 | 203116 | | 203145 | 5.20E-08 |
| 100583 | 100612 | F | 30 | 245256 | | 245285 | 5.20E-08 |
| 100583 | 100612 | P | 30 | 457646 | | 457675 | 5.20E-08 |
| 100583 | 100612 | P | 30 | 457675 | | 457704 | 5.20E-08 |
| 100611 | 100640 | P | 30 | 203116 | | 203145 | 5.20E-08 |
| 100611 | 100640 | F | 30 | 245256 | | 245285 | 5.20E-08 |
| 100611 | 100640 | P | 30 | 457646 | | 457675 | 5.20E-08 |
| 100611 | 100640 | P | 30 | 457675 | | 457704 | 5.20E-08 |
| 116871 | 116900 | P | 30 | 184511 | | 184540 | 5.20E-08 |
| 117331 | 117360 | P | 30 | 185971 | | 186000 | 5.20E-08 |
| 118564 | 118593 | P | 30 | 209142 | | 209171 | 5.20E-08 |
| 139489 | 139518 | P | 30 | 180411 | | 180440 | 5.20E-08 |
| 168397 | 168426 | F | 30 | 222539 | | 222568 | 5.20E-08 |
| 179794 | 179823 | F | 30 | 203116 | | 203145 | 5.20E-08 |
| 179794 | 179823 | P | 30 | 245256 | | 245285 | 5.20E-08 |
| 179794 | 179823 | F | 30 | 457646 | | 457675 | 5.20E-08 |
| 179794 | 179823 | F | 30 | 457675 | | 457704 | 5.20E-08 |
| 190922 | 190951 | P | 30 | 451950 | | 451979 | 5.20E-08 |
| 203116 | 203145 | P | 30 | 290601 | | 290630 | 5.20E-08 |
| 203116 | 203145 | P | 30 | 339819 | | 339848 | 5.20E-08 |
| 245256 | 245285 | F | 30 | 290601 | | 290630 | 5.20E-08 |
| 245256 | 245285 | F | 30 | 339819 | | 339848 | 5.20E-08 |
| 290601 | 290630 | P | 30 | 457646 | | 457675 | 5.20E-08 |
| 290601 | 290630 | P | 30 | 457675 | | 457704 | 5.20E-08 |
| 339819 | 339848 | P | 30 | 457646 | | 457675 | 5.20E-08 |
| 339819 | 339848 | P | 30 | 457675 | | 457704 | 5.20E-08 |
| 28119 | 28154 | F | 36 | 100448 | | 100483 | 7.20E-08 |
| 28119 | 28154 | F | 36 | 100476 | | 100511 | 7.20E-08 |
| 28119 | 28154 | F | 36 | 100504 | | 100539 | 7.20E-08 |
| 28119 | 28154 | F | 36 | 100532 | | 100567 | 7.20E-08 |
| 28119 | 28154 | F | 36 | 100560 | | 100595 | 7.20E-08 |
| 28119 | 28154 | F | 36 | 100588 | | 100623 | 7.20E-08 |
| 33973 | 34008 | F | 36 | 413950 | | 413985 | 7.20E-08 |
| 85870 | 85905 | P | 36 | 180384 | | 180419 | 7.20E-08 |
| 100448 | 100483 | F | 36 | 298497 | | 298532 | 7.20E-08 |
| 100476 | 100511 | F | 36 | 298497 | | 298532 | 7.20E-08 |
| 100504 | 100539 | F | 36 | 298497 | | 298532 | 7.20E-08 |
| 100532 | 100567 | F | 36 | 298497 | | 298532 | 7.20E-08 |
| 100560 | 100595 | F | 36 | 298497 | | 298532 | 7.20E-08 |
| 100588 | 100623 | F | 36 | 298497 | | 298532 | 7.20E-08 |
| 100616 | 100651 | F | 36 | 258735 | | 258770 | 7.20E-08 |
| 124162 | 124197 | P | 36 | 179769 | | 179804 | 7.20E-08 |
| 139490 | 139525 | F | 36 | 308631 | | 308666 | 7.20E-08 |
| 179783 | 179818 | P | 36 | 258735 | | 258770 | 7.20E-08 |
| 210412 | 210447 | P | 36 | 308631 | | 308666 | 7.20E-08 |
| 258735 | 258770 | F | 36 | 339824 | | 339859 | 7.20E-08 |
| 258760 | 258795 | P | 36 | 308631 | | 308666 | 7.20E-08 |
| 308631 | 308666 | P | 36 | 382758 | | 382793 | 7.20E-08 |
| 23783 | 23815 | F | 33 | 206218 | | 206250 | 8.04E-08 |
| 99367 | 99399 | F | 33 | 149726 | | 149758 | 8.04E-08 |
| 180384 | 180416 | F | 33 | 409377 | | 409409 | 8.04E-08 |
| 206684 | 206716 | F | 33 | 221646 | | 221678 | 8.04E-08 |
| 18330 | 18367 | F | 38 | 65859 | | 65896 | 1.81E-07 |
| 27392 | 27429 | F | 38 | 49660 | | 49697 | 1.81E-07 |
| 49660 | 49697 | F | 38 | 357900 | | 357937 | 1.81E-07 |
| 60193 | 60230 | F | 38 | 431169 | | 431206 | 1.81E-07 |
| 65859 | 65896 | F | 38 | 153007 | | 153044 | 1.81E-07 |
| 65859 | 65896 | F | 38 | 179814 | | 179851 | 1.81E-07 |
| 65859 | 65896 | P | 38 | 210439 | | 210476 | 1.81E-07 |
| 65859 | 65896 | F | 38 | 226145 | | 226182 | 1.81E-07 |
| 65859 | 65896 | P | 38 | 382728 | | 382765 | 1.81E-07 |
| 65859 | 65896 | F | 38 | 397151 | | 397188 | 1.81E-07 |
| 139490 | 139527 | P | 38 | 432986 | | 433023 | 1.81E-07 |
| 224039 | 224076 | F | 38 | 285744 | | 285781 | 1.81E-07 |
| 4014 | 4048 | F | 35 | 48743 | | 48777 | 2.72E-07 |
| 4014 | 4048 | P | 35 | 432990 | | 433024 | 2.72E-07 |
| 14130 | 14164 | F | 35 | 409374 | | 409408 | 2.72E-07 |
| 18301 | 18335 | F | 35 | 253361 | | 253395 | 2.72E-07 |
| 18301 | 18335 | P | 35 | 324904 | | 324938 | 2.72E-07 |
| 30887 | 30921 | P | 35 | 127111 | | 127145 | 2.72E-07 |
| 40586 | 40620 | P | 35 | 320192 | | 320226 | 2.72E-07 |
| 48742 | 48776 | P | 35 | 210415 | | 210449 | 2.72E-07 |
| 48742 | 48776 | P | 35 | 258763 | | 258797 | 2.72E-07 |
| 54522 | 54556 | P | 35 | 190169 | | 190203 | 2.72E-07 |
| 54523 | 54557 | F | 35 | 153007 | | 153041 | 2.72E-07 |
| 54523 | 54557 | F | 35 | 397151 | | 397185 | 2.72E-07 |
| 98493 | 98527 | F | 35 | 288321 | | 288355 | 2.72E-07 |
| 100447 | 100481 | P | 35 | 253361 | | 253395 | 2.72E-07 |
| 100447 | 100481 | F | 35 | 324904 | | 324938 | 2.72E-07 |
| 100475 | 100509 | P | 35 | 253361 | | 253395 | 2.72E-07 |
| 100475 | 100509 | F | 35 | 324904 | | 324938 | 2.72E-07 |
| 100503 | 100537 | P | 35 | 253361 | | 253395 | 2.72E-07 |
| 100503 | 100537 | F | 35 | 324904 | | 324938 | 2.72E-07 |
| 100531 | 100565 | P | 35 | 253361 | | 253395 | 2.72E-07 |
| 100531 | 100565 | F | 35 | 324904 | | 324938 | 2.72E-07 |
| 100559 | 100593 | P | 35 | 253361 | | 253395 | 2.72E-07 |
| 100559 | 100593 | F | 35 | 324904 | | 324938 | 2.72E-07 |
| 100587 | 100621 | P | 35 | 253361 | | 253395 | 2.72E-07 |
| 100587 | 100621 | F | 35 | 324904 | | 324938 | 2.72E-07 |
| 100615 | 100649 | P | 35 | 253361 | | 253395 | 2.72E-07 |
| 100615 | 100649 | F | 35 | 324904 | | 324938 | 2.72E-07 |
| 153007 | 153041 | F | 35 | 219139 | | 219173 | 2.72E-07 |
| 153007 | 153041 | P | 35 | 424214 | | 424248 | 2.72E-07 |
| 179785 | 179819 | F | 35 | 253361 | | 253395 | 2.72E-07 |
| 179785 | 179819 | P | 35 | 324904 | | 324938 | 2.72E-07 |
| 203108 | 203142 | P | 35 | 409374 | | 409408 | 2.72E-07 |
| 210415 | 210449 | F | 35 | 432991 | | 433025 | 2.72E-07 |
| 210469 | 210503 | P | 35 | 308632 | | 308666 | 2.72E-07 |
| 245259 | 245293 | F | 35 | 409374 | | 409408 | 2.72E-07 |
| 253361 | 253395 | P | 35 | 339823 | | 339857 | 2.72E-07 |
| 258763 | 258797 | F | 35 | 432991 | | 433025 | 2.72E-07 |
| 264911 | 264945 | F | 35 | 456731 | | 456765 | 2.72E-07 |
| 324904 | 324938 | F | 35 | 339823 | | 339857 | 2.72E-07 |
| 409374 | 409408 | P | 35 | 457638 | | 457672 | 2.72E-07 |
| 409374 | 409408 | P | 35 | 457667 | | 457701 | 2.72E-07 |
| 14133 | 14164 | F | 32 | 180384 | | 180415 | 3.12E-07 |
| 27425 | 27456 | P | 32 | 180647 | | 180678 | 3.12E-07 |
| 99337 | 99368 | F | 32 | 288759 | | 288790 | 3.12E-07 |
| 180384 | 180415 | P | 32 | 203108 | | 203139 | 3.12E-07 |
| 180384 | 180415 | F | 32 | 245262 | | 245293 | 3.12E-07 |
| 180384 | 180415 | P | 32 | 457638 | | 457669 | 3.12E-07 |
| 180384 | 180415 | P | 32 | 457667 | | 457698 | 3.12E-07 |
| 180384 | 180415 | P | 32 | 457696 | | 457727 | 3.12E-07 |
| 180647 | 180678 | P | 32 | 357933 | | 357964 | 3.12E-07 |
| 276604 | 276635 | P | 32 | 438600 | | 438631 | 3.12E-07 |
| 1076 | 1112 | F | 37 | 65860 | | 65896 | 6.66E-07 |
| 65860 | 65896 | P | 37 | 401864 | | 401900 | 6.66E-07 |
| 66449 | 66485 | F | 37 | 393874 | | 393910 | 6.66E-07 |
| 97489 | 97525 | P | 37 | 405648 | | 405684 | 6.66E-07 |
| 180824 | 180860 | P | 37 | 405648 | | 405684 | 6.66E-07 |
| 192421 | 192457 | P | 37 | 278754 | | 278790 | 6.66E-07 |
| 460888 | 460924 | C | 37 | 460891 | | 460927 | 6.66E-07 |
| 1076 | 1109 | F | 34 | 54524 | | 54557 | 1.03E-06 |
| 1076 | 1109 | F | 34 | 219140 | | 219173 | 1.03E-06 |
| 1076 | 1109 | P | 34 | 424214 | | 424247 | 1.03E-06 |
| 4014 | 4047 | F | 34 | 18301 | | 18334 | 1.03E-06 |
| 4014 | 4047 | P | 34 | 100448 | | 100481 | 1.03E-06 |
| 4014 | 4047 | P | 34 | 100476 | | 100509 | 1.03E-06 |
| 4014 | 4047 | P | 34 | 100504 | | 100537 | 1.03E-06 |
| 4014 | 4047 | P | 34 | 100532 | | 100565 | 1.03E-06 |
| 4014 | 4047 | P | 34 | 100560 | | 100593 | 1.03E-06 |
| 4014 | 4047 | P | 34 | 100588 | | 100621 | 1.03E-06 |
| 4014 | 4047 | P | 34 | 100616 | | 100649 | 1.03E-06 |
| 4014 | 4047 | F | 34 | 179785 | | 179818 | 1.03E-06 |
| 4014 | 4047 | P | 34 | 339824 | | 339857 | 1.03E-06 |
| 18270 | 18303 | P | 34 | 453897 | | 453930 | 1.03E-06 |
| 18301 | 18334 | P | 34 | 28119 | | 28152 | 1.03E-06 |
| 18301 | 18334 | P | 34 | 258735 | | 258768 | 1.03E-06 |
| 18301 | 18334 | P | 34 | 298497 | | 298530 | 1.03E-06 |
| 48743 | 48776 | F | 34 | 253361 | | 253394 | 1.03E-06 |
| 48743 | 48776 | P | 34 | 324905 | | 324938 | 1.03E-06 |
| 54524 | 54557 | P | 34 | 401867 | | 401900 | 1.03E-06 |
| 65859 | 65892 | P | 34 | 100419 | | 100452 | 1.03E-06 |
| 65859 | 65892 | P | 34 | 266485 | | 266518 | 1.03E-06 |
| 219140 | 219173 | P | 34 | 401867 | | 401900 | 1.03E-06 |
| 253361 | 253394 | P | 34 | 432991 | | 433024 | 1.03E-06 |
| 253362 | 253395 | F | 34 | 308631 | | 308664 | 1.03E-06 |
| 308631 | 308664 | P | 34 | 324904 | | 324937 | 1.03E-06 |
| 324905 | 324938 | F | 34 | 432991 | | 433024 | 1.03E-06 |
| 401867 | 401900 | F | 34 | 424214 | | 424247 | 1.03E-06 |
| 460888 | 460921 | P | 34 | 460888 | | 460921 | 1.03E-06 |
| 460888 | 460921 | C | 34 | 460889 | | 460922 | 1.03E-06 |
| 460889 | 460922 | P | 34 | 460889 | | 460922 | 1.03E-06 |
| 4015 | 4045 | P | 31 | 180409 | | 180439 | 1.21E-06 |
| 12650 | 12680 | F | 31 | 431201 | | 431231 | 1.21E-06 |
| 18310 | 18340 | F | 31 | 457704 | | 457734 | 1.21E-06 |
| 23251 | 23281 | F | 31 | 444131 | | 444161 | 1.21E-06 |
| 23795 | 23825 | F | 31 | 199206 | | 199236 | 1.21E-06 |
| 23906 | 23936 | P | 31 | 114828 | | 114858 | 1.21E-06 |
| 28121 | 28151 | F | 31 | 180409 | | 180439 | 1.21E-06 |
| 57506 | 57536 | F | 31 | 206212 | | 206242 | 1.21E-06 |
| 60198 | 60228 | F | 31 | 397097 | | 397127 | 1.21E-06 |
| 100442 | 100472 | P | 31 | 457704 | | 457734 | 1.21E-06 |
| 100470 | 100500 | P | 31 | 457704 | | 457734 | 1.21E-06 |
| 100498 | 100528 | P | 31 | 457704 | | 457734 | 1.21E-06 |
| 100526 | 100556 | P | 31 | 457704 | | 457734 | 1.21E-06 |
| 100554 | 100584 | P | 31 | 457704 | | 457734 | 1.21E-06 |
| 100582 | 100612 | P | 31 | 457704 | | 457734 | 1.21E-06 |
| 100610 | 100640 | P | 31 | 457704 | | 457734 | 1.21E-06 |
| 106982 | 107012 | P | 31 | 107954 | | 107984 | 1.21E-06 |
| 118557 | 118587 | P | 31 | 397097 | | 397127 | 1.21E-06 |
| 120238 | 120268 | P | 31 | 345466 | | 345496 | 1.21E-06 |
| 136203 | 136233 | F | 31 | 378251 | | 378281 | 1.21E-06 |
| 179794 | 179824 | F | 31 | 457704 | | 457734 | 1.21E-06 |
| 180409 | 180439 | F | 31 | 210417 | | 210447 | 1.21E-06 |
| 180409 | 180439 | P | 31 | 253362 | | 253392 | 1.21E-06 |
| 180409 | 180439 | F | 31 | 258737 | | 258767 | 1.21E-06 |
| 180409 | 180439 | F | 31 | 258765 | | 258795 | 1.21E-06 |
| 180409 | 180439 | F | 31 | 298499 | | 298529 | 1.21E-06 |
| 180409 | 180439 | F | 31 | 324907 | | 324937 | 1.21E-06 |
| 199188 | 199218 | P | 31 | 378251 | | 378281 | 1.21E-06 |
| 290600 | 290630 | P | 31 | 457704 | | 457734 | 1.21E-06 |
| 339818 | 339848 | P | 31 | 457704 | | 457734 | 1.21E-06 |
| 360416 | 360446 | F | 31 | 364698 | | 364728 | 1.21E-06 |
| 459385 | 459415 | P | 31 | 460897 | | 460927 | 1.21E-06 |
| 53229 | 53264 | F | 36 | 118556 | | 118591 | 2.45E-06 |
| 99017 | 99052 | F | 36 | 288484 | | 288519 | 2.45E-06 |
| 137981 | 138016 | F | 36 | 325531 | | 325566 | 2.45E-06 |
| 236676 | 236711 | P | 36 | 305304 | | 305339 | 2.45E-06 |
| 264681 | 264716 | F | 36 | 456515 | | 456550 | 2.45E-06 |
| 280427 | 280462 | P | 36 | 357862 | | 357897 | 2.45E-06 |
| 4015 | 4047 | F | 33 | 308631 | | 308663 | 3.86E-06 |
| 23782 | 23814 | F | 33 | 143760 | | 143792 | 3.86E-06 |
| 28119 | 28151 | P | 33 | 308631 | | 308663 | 3.86E-06 |
| 48744 | 48776 | F | 33 | 139490 | | 139522 | 3.86E-06 |
| 48744 | 48776 | P | 33 | 382761 | | 382793 | 3.86E-06 |
| 54524 | 54556 | P | 33 | 443585 | | 443617 | 3.86E-06 |
| 85874 | 85906 | P | 33 | 409376 | | 409408 | 3.86E-06 |
| 114827 | 114859 | F | 33 | 206333 | | 206365 | 3.86E-06 |
| 175586 | 175618 | P | 33 | 202131 | | 202163 | 3.86E-06 |
| 219140 | 219172 | P | 33 | 443585 | | 443617 | 3.86E-06 |
| 253363 | 253395 | P | 33 | 290605 | | 290637 | 3.86E-06 |
| 257732 | 257764 | F | 33 | 415189 | | 415221 | 3.86E-06 |
| 258735 | 258767 | P | 33 | 308631 | | 308663 | 3.86E-06 |
| 290605 | 290637 | F | 33 | 324904 | | 324936 | 3.86E-06 |
| 298497 | 298529 | P | 33 | 308631 | | 308663 | 3.86E-06 |
| 325908 | 325940 | P | 33 | 394318 | | 394350 | 3.86E-06 |
| 382761 | 382793 | F | 33 | 432991 | | 433023 | 3.86E-06 |
| 424215 | 424247 | F | 33 | 443585 | | 443617 | 3.86E-06 |
| 460888 | 460920 | R | 33 | 460888 | | 460920 | 3.86E-06 |
| 460888 | 460920 | F | 33 | 460890 | | 460922 | 3.86E-06 |
| 460890 | 460922 | R | 33 | 460890 | | 460922 | 3.86E-06 |
| 460895 | 460927 | R | 33 | 460895 | | 460927 | 3.86E-06 |
| 12650 | 12679 | P | 30 | 53204 | | 53233 | 4.68E-06 |
| 18310 | 18339 | F | 30 | 85882 | | 85911 | 4.68E-06 |
| 26784 | 26813 | F | 30 | 298011 | | 298040 | 4.68E-06 |
| 58740 | 58769 | P | 30 | 355791 | | 355820 | 4.68E-06 |
| 85882 | 85911 | P | 30 | 100443 | | 100472 | 4.68E-06 |
| 85882 | 85911 | P | 30 | 100471 | | 100500 | 4.68E-06 |
| 85882 | 85911 | P | 30 | 100499 | | 100528 | 4.68E-06 |
| 85882 | 85911 | P | 30 | 100527 | | 100556 | 4.68E-06 |
| 85882 | 85911 | P | 30 | 100555 | | 100584 | 4.68E-06 |
| 85882 | 85911 | P | 30 | 100583 | | 100612 | 4.68E-06 |
| 85882 | 85911 | P | 30 | 100611 | | 100640 | 4.68E-06 |
| 85882 | 85911 | F | 30 | 179794 | | 179823 | 4.68E-06 |
| 85882 | 85911 | P | 30 | 290601 | | 290630 | 4.68E-06 |
| 85882 | 85911 | P | 30 | 339819 | | 339848 | 4.68E-06 |
| 97958 | 97987 | F | 30 | 287786 | | 287815 | 4.68E-06 |
| 98751 | 98780 | P | 30 | 431169 | | 431198 | 4.68E-06 |
| 99190 | 99219 | F | 30 | 288659 | | 288688 | 4.68E-06 |
| 107825 | 107854 | F | 30 | 111647 | | 111676 | 4.68E-06 |
| 118563 | 118592 | P | 30 | 431169 | | 431198 | 4.68E-06 |
| 120627 | 120656 | F | 30 | 341851 | | 341880 | 4.68E-06 |
| 137502 | 137531 | F | 30 | 195131 | | 195160 | 4.68E-06 |
| 180409 | 180438 | F | 30 | 210474 | | 210503 | 4.68E-06 |
| 197567 | 197596 | P | 30 | 355791 | | 355820 | 4.68E-06 |
| 203116 | 203145 | P | 30 | 432986 | | 433015 | 4.68E-06 |
| 218918 | 218947 | P | 30 | 290075 | | 290104 | 4.68E-06 |
| 245256 | 245285 | F | 30 | 432986 | | 433015 | 4.68E-06 |
| 271568 | 271597 | P | 30 | 278722 | | 278751 | 4.68E-06 |
| 287535 | 287564 | F | 30 | 314941 | | 314970 | 4.68E-06 |
| 308977 | 309006 | P | 30 | 443590 | | 443619 | 4.68E-06 |
| 432986 | 433015 | P | 30 | 457646 | | 457675 | 4.68E-06 |
| 432986 | 433015 | P | 30 | 457675 | | 457704 | 4.68E-06 |
| 459388 | 459417 | F | 30 | 460895 | | 460924 | 4.68E-06 |
| 459388 | 459417 | R | 30 | 460898 | | 460927 | 4.68E-06 |
| 24506 | 24540 | F | 35 | 296094 | | 296128 | 8.97E-06 |
| 29465 | 29499 | P | 35 | 128538 | | 128572 | 8.97E-06 |
| 30300 | 30334 | P | 35 | 127691 | | 127725 | 8.97E-06 |
| 34142 | 34176 | P | 35 | 369546 | | 369580 | 8.97E-06 |
| 53230 | 53264 | P | 35 | 60194 | | 60228 | 8.97E-06 |
| 98501 | 98535 | F | 35 | 288329 | | 288363 | 8.97E-06 |
| 233609 | 233643 | P | 35 | 344366 | | 344400 | 8.97E-06 |
| 261501 | 261535 | P | 35 | 292347 | | 292381 | 8.97E-06 |
| 264604 | 264638 | F | 35 | 456431 | | 456465 | 8.97E-06 |
| 4016 | 4047 | P | 32 | 290606 | | 290637 | 1.45E-05 |
| 28119 | 28150 | F | 32 | 290606 | | 290637 | 1.45E-05 |
| 43040 | 43071 | F | 32 | 43094 | | 43125 | 1.45E-05 |
| 48745 | 48776 | P | 32 | 210472 | | 210503 | 1.45E-05 |
| 75079 | 75110 | F | 32 | 230420 | | 230451 | 1.45E-05 |
| 151648 | 151679 | P | 32 | 226651 | | 226682 | 1.45E-05 |
| 168438 | 168469 | F | 32 | 379453 | | 379484 | 1.45E-05 |
| 180061 | 180092 | P | 32 | 184377 | | 184408 | 1.45E-05 |
| 180534 | 180565 | F | 32 | 184815 | | 184846 | 1.45E-05 |
| 185106 | 185137 | P | 32 | 335607 | | 335638 | 1.45E-05 |
| 210472 | 210503 | F | 32 | 432991 | | 433022 | 1.45E-05 |
| 258735 | 258766 | F | 32 | 290606 | | 290637 | 1.45E-05 |
| 258744 | 258775 | P | 32 | 416181 | | 416212 | 1.45E-05 |
| 290606 | 290637 | F | 32 | 298497 | | 298528 | 1.45E-05 |
| 459386 | 459417 | C | 32 | 460886 | | 460917 | 1.45E-05 |
| 460888 | 460919 | P | 32 | 460888 | | 460919 | 1.45E-05 |
| 16647 | 16680 | P | 34 | 71285 | | 71318 | 3.28E-05 |
| 17786 | 17819 | P | 34 | 83812 | | 83845 | 3.28E-05 |
| 48138 | 48171 | P | 34 | 378789 | | 378822 | 3.28E-05 |
| 98217 | 98250 | F | 34 | 288040 | | 288073 | 3.28E-05 |
| 98226 | 98259 | F | 34 | 288049 | | 288082 | 3.28E-05 |
| 144370 | 144403 | P | 34 | 448990 | | 449023 | 3.28E-05 |
| 205004 | 205037 | P | 34 | 231107 | | 231140 | 3.28E-05 |
| 229684 | 229717 | P | 34 | 433194 | | 433227 | 3.28E-05 |
| 238824 | 238857 | F | 34 | 238860 | | 238893 | 3.28E-05 |
| 459384 | 459417 | R | 34 | 460892 | | 460925 | 3.28E-05 |
| 6622 | 6652 | F | 31 | 179769 | | 179799 | 5.44E-05 |
| 27605 | 27635 | F | 31 | 276526 | | 276556 | 5.44E-05 |
| 48752 | 48782 | F | 31 | 85882 | | 85912 | 5.44E-05 |
| 72169 | 72199 | P | 31 | 308795 | | 308825 | 5.44E-05 |
| 122434 | 122464 | F | 31 | 448958 | | 448988 | 5.44E-05 |
| 136226 | 136256 | P | 31 | 143731 | | 143761 | 5.44E-05 |
| 137709 | 137739 | P | 31 | 419803 | | 419833 | 5.44E-05 |
| 145705 | 145735 | P | 31 | 402968 | | 402998 | 5.44E-05 |
| 149458 | 149488 | P | 31 | 203125 | | 203155 | 5.44E-05 |
| 166350 | 166380 | P | 31 | 435469 | | 435499 | 5.44E-05 |
| 175808 | 175838 | P | 31 | 450223 | | 450253 | 5.44E-05 |
| 197347 | 197377 | P | 31 | 355867 | | 355897 | 5.44E-05 |
| 205082 | 205112 | F | 31 | 209354 | | 209384 | 5.44E-05 |
| 227988 | 228018 | F | 31 | 451326 | | 451356 | 5.44E-05 |
| 238809 | 238839 | F | 31 | 238863 | | 238893 | 5.44E-05 |
| 275103 | 275133 | F | 31 | 291301 | | 291331 | 5.44E-05 |
| 317736 | 317766 | F | 31 | 434412 | | 434442 | 5.44E-05 |
| 432985 | 433015 | P | 31 | 457704 | | 457734 | 5.44E-05 |
| 445386 | 445416 | P | 31 | 449006 | | 449036 | 5.44E-05 |
| 459387 | 459417 | P | 31 | 460893 | | 460923 | 5.44E-05 |
| 460888 | 460918 | R | 31 | 460888 | | 460918 | 5.44E-05 |
| 460888 | 460918 | F | 31 | 460892 | | 460922 | 5.44E-05 |
| 460892 | 460922 | R | 31 | 460892 | | 460922 | 5.44E-05 |
| 18298 | 18330 | P | 33 | 180411 | | 180443 | 1.20E-04 |
| 24969 | 25001 | F | 33 | 296573 | | 296605 | 1.20E-04 |
| 34002 | 34034 | P | 33 | 369677 | | 369709 | 1.20E-04 |
| 98983 | 99015 | F | 33 | 288450 | | 288482 | 1.20E-04 |
| 128116 | 128148 | P | 33 | 146413 | | 146445 | 1.20E-04 |
| 137992 | 138024 | F | 33 | 325542 | | 325574 | 1.20E-04 |
| 194740 | 194772 | F | 33 | 273798 | | 273830 | 1.20E-04 |
| 236714 | 236746 | P | 33 | 305270 | | 305302 | 1.20E-04 |
| 316917 | 316949 | F | 33 | 432816 | | 432848 | 1.20E-04 |
| 319268 | 319300 | F | 33 | 460295 | | 460327 | 1.20E-04 |
| 337679 | 337711 | P | 33 | 337679 | | 337711 | 1.20E-04 |
| 435672 | 435704 | P | 33 | 438669 | | 438701 | 1.20E-04 |
| 14127 | 14156 | P | 30 | 48752 | | 48781 | 2.04E-04 |
| 14129 | 14158 | P | 30 | 18308 | | 18337 | 2.04E-04 |
| 14129 | 14158 | F | 30 | 100445 | | 100474 | 2.04E-04 |
| 14129 | 14158 | F | 30 | 100473 | | 100502 | 2.04E-04 |
| 14129 | 14158 | F | 30 | 100501 | | 100530 | 2.04E-04 |
| 14129 | 14158 | F | 30 | 100529 | | 100558 | 2.04E-04 |
| 14129 | 14158 | F | 30 | 100557 | | 100586 | 2.04E-04 |
| 14129 | 14158 | F | 30 | 100585 | | 100614 | 2.04E-04 |
| 14129 | 14158 | F | 30 | 100613 | | 100642 | 2.04E-04 |
| 14129 | 14158 | P | 30 | 179792 | | 179821 | 2.04E-04 |
| 14129 | 14158 | F | 30 | 290603 | | 290632 | 2.04E-04 |
| 14129 | 14158 | F | 30 | 339821 | | 339850 | 2.04E-04 |
| 17020 | 17049 | P | 30 | 27862 | | 27891 | 2.04E-04 |
| 18311 | 18340 | P | 30 | 409370 | | 409399 | 2.04E-04 |
| 18324 | 18353 | P | 30 | 124157 | | 124186 | 2.04E-04 |
| 18336 | 18365 | F | 30 | 219145 | | 219174 | 2.04E-04 |
| 18336 | 18365 | P | 30 | 424213 | | 424242 | 2.04E-04 |
| 28128 | 28157 | F | 30 | 124157 | | 124186 | 2.04E-04 |
| 31124 | 31153 | P | 30 | 126880 | | 126909 | 2.04E-04 |
| 31445 | 31474 | P | 30 | 417452 | | 417481 | 2.04E-04 |
| 53237 | 53266 | F | 30 | 98752 | | 98781 | 2.04E-04 |
| 54537 | 54566 | P | 30 | 266475 | | 266504 | 2.04E-04 |
| 58309 | 58338 | P | 30 | 60163 | | 60192 | 2.04E-04 |
| 59364 | 59393 | F | 30 | 59421 | | 59450 | 2.04E-04 |
| 60164 | 60193 | F | 30 | 87381 | | 87410 | 2.04E-04 |
| 87406 | 87435 | P | 30 | 448925 | | 448954 | 2.04E-04 |
| 100429 | 100458 | F | 30 | 124157 | | 124186 | 2.04E-04 |
| 100442 | 100471 | F | 30 | 409370 | | 409399 | 2.04E-04 |
| 100470 | 100499 | F | 30 | 409370 | | 409399 | 2.04E-04 |
| 100498 | 100527 | F | 30 | 409370 | | 409399 | 2.04E-04 |
| 100526 | 100555 | F | 30 | 409370 | | 409399 | 2.04E-04 |
| 100554 | 100583 | F | 30 | 409370 | | 409399 | 2.04E-04 |
| 100582 | 100611 | F | 30 | 409370 | | 409399 | 2.04E-04 |
| 100610 | 100639 | F | 30 | 409370 | | 409399 | 2.04E-04 |
| 117380 | 117409 | P | 30 | 206213 | | 206242 | 2.04E-04 |
| 124157 | 124186 | P | 30 | 179808 | | 179837 | 2.04E-04 |
| 124157 | 124186 | F | 30 | 210396 | | 210425 | 2.04E-04 |
| 124157 | 124186 | F | 30 | 210453 | | 210482 | 2.04E-04 |
| 124157 | 124186 | F | 30 | 290587 | | 290616 | 2.04E-04 |
| 124157 | 124186 | F | 30 | 298506 | | 298535 | 2.04E-04 |
| 124157 | 124186 | F | 30 | 339805 | | 339834 | 2.04E-04 |
| 124157 | 124186 | F | 30 | 382742 | | 382771 | 2.04E-04 |
| 124157 | 124186 | P | 30 | 416183 | | 416212 | 2.04E-04 |
| 134378 | 134407 | P | 30 | 434341 | | 434370 | 2.04E-04 |
| 139498 | 139527 | F | 30 | 203116 | | 203145 | 2.04E-04 |
| 139498 | 139527 | P | 30 | 245256 | | 245285 | 2.04E-04 |
| 139498 | 139527 | F | 30 | 457646 | | 457675 | 2.04E-04 |
| 139498 | 139527 | F | 30 | 457675 | | 457704 | 2.04E-04 |
| 147534 | 147563 | F | 30 | 309086 | | 309115 | 2.04E-04 |
| 179795 | 179824 | P | 30 | 409370 | | 409399 | 2.04E-04 |
| 179820 | 179849 | F | 30 | 219145 | | 219174 | 2.04E-04 |
| 179820 | 179849 | P | 30 | 424213 | | 424242 | 2.04E-04 |
| 192636 | 192665 | P | 30 | 227646 | | 227675 | 2.04E-04 |
| 198419 | 198448 | P | 30 | 211042 | | 211071 | 2.04E-04 |
| 203116 | 203145 | P | 30 | 210410 | | 210439 | 2.04E-04 |
| 203116 | 203145 | P | 30 | 210467 | | 210496 | 2.04E-04 |
| 203116 | 203145 | P | 30 | 258758 | | 258787 | 2.04E-04 |
| 203116 | 203145 | F | 30 | 308639 | | 308668 | 2.04E-04 |
| 203116 | 203145 | P | 30 | 382756 | | 382785 | 2.04E-04 |
| 210410 | 210439 | F | 30 | 245256 | | 245285 | 2.04E-04 |
| 210410 | 210439 | P | 30 | 457646 | | 457675 | 2.04E-04 |
| 210410 | 210439 | P | 30 | 457675 | | 457704 | 2.04E-04 |
| 210467 | 210496 | F | 30 | 245256 | | 245285 | 2.04E-04 |
| 210467 | 210496 | P | 30 | 457646 | | 457675 | 2.04E-04 |
| 210467 | 210496 | P | 30 | 457675 | | 457704 | 2.04E-04 |
| 213389 | 213418 | P | 30 | 433961 | | 433990 | 2.04E-04 |
| 219128 | 219157 | P | 30 | 258749 | | 258778 | 2.04E-04 |
| 219145 | 219174 | P | 30 | 266483 | | 266512 | 2.04E-04 |
| 219145 | 219174 | P | 30 | 382730 | | 382759 | 2.04E-04 |
| 222325 | 222354 | F | 30 | 346296 | | 346325 | 2.04E-04 |
| 229705 | 229734 | P | 30 | 433165 | | 433194 | 2.04E-04 |
| 245256 | 245285 | F | 30 | 258758 | | 258787 | 2.04E-04 |
| 245256 | 245285 | F | 30 | 382756 | | 382785 | 2.04E-04 |
| 245258 | 245287 | P | 30 | 308637 | | 308666 | 2.04E-04 |
| 258749 | 258778 | F | 30 | 424230 | | 424259 | 2.04E-04 |
| 258758 | 258787 | P | 30 | 457646 | | 457675 | 2.04E-04 |
| 258758 | 258787 | P | 30 | 457675 | | 457704 | 2.04E-04 |
| 261922 | 261951 | P | 30 | 374350 | | 374379 | 2.04E-04 |
| 275158 | 275187 | F | 30 | 291353 | | 291382 | 2.04E-04 |
| 289452 | 289481 | P | 30 | 373545 | | 373574 | 2.04E-04 |
| 290600 | 290629 | F | 30 | 409370 | | 409399 | 2.04E-04 |
| 308639 | 308668 | F | 30 | 457646 | | 457675 | 2.04E-04 |
| 308639 | 308668 | F | 30 | 457675 | | 457704 | 2.04E-04 |
| 334618 | 334647 | F | 30 | 433259 | | 433288 | 2.04E-04 |
| 339818 | 339847 | F | 30 | 409370 | | 409399 | 2.04E-04 |
| 376577 | 376606 | P | 30 | 431318 | | 431347 | 2.04E-04 |
| 378160 | 378189 | F | 30 | 394872 | | 394901 | 2.04E-04 |
| 382756 | 382785 | P | 30 | 457646 | | 457675 | 2.04E-04 |
| 382756 | 382785 | P | 30 | 457675 | | 457704 | 2.04E-04 |
| 399833 | 399862 | P | 30 | 400964 | | 400993 | 2.04E-04 |
| 424765 | 424794 | F | 30 | 456912 | | 456941 | 2.04E-04 |
| 459385 | 459414 | C | 30 | 459388 | | 459417 | 2.04E-04 |
| 459387 | 459416 | F | 30 | 459389 | | 459418 | 2.04E-04 |
| 459387 | 459416 | F | 30 | 460896 | | 460925 | 2.04E-04 |
| 459387 | 459416 | R | 30 | 460897 | | 460926 | 2.04E-04 |
| 459388 | 459417 | P | 30 | 459388 | | 459417 | 2.04E-04 |
| 459388 | 459417 | R | 30 | 460888 | | 460917 | 2.04E-04 |
| 459388 | 459417 | P | 30 | 460889 | | 460918 | 2.04E-04 |
| 459388 | 459417 | F | 30 | 460889 | | 460918 | 2.04E-04 |
| 459388 | 459417 | C | 30 | 460890 | | 460919 | 2.04E-04 |
| 459388 | 459417 | R | 30 | 460890 | | 460919 | 2.04E-04 |
| 459388 | 459417 | P | 30 | 460891 | | 460920 | 2.04E-04 |
| 459388 | 459417 | F | 30 | 460891 | | 460920 | 2.04E-04 |
| 459388 | 459417 | C | 30 | 460892 | | 460921 | 2.04E-04 |
| 459388 | 459417 | F | 30 | 460893 | | 460922 | 2.04E-04 |
| 459389 | 459418 | C | 30 | 460887 | | 460916 | 2.04E-04 |
| 459390 | 459419 | C | 30 | 459391 | | 459420 | 2.04E-04 |
| 460887 | 460916 | C | 30 | 460896 | | 460925 | 2.04E-04 |
| 460888 | 460917 | P | 30 | 460888 | | 460917 | 2.04E-04 |
| 460888 | 460917 | C | 30 | 460893 | | 460922 | 2.04E-04 |
| 460893 | 460922 | P | 30 | 460893 | | 460922 | 2.04E-04 |
| 1076 | 1107 | F | 32 | 308979 | | 309010 | 4.35E-04 |
| 8948 | 8979 | P | 32 | 280543 | | 280574 | 4.35E-04 |
| 18331 | 18362 | F | 32 | 308979 | | 309010 | 4.35E-04 |
| 29411 | 29442 | P | 32 | 128595 | | 128626 | 4.35E-04 |
| 30082 | 30113 | F | 32 | 146614 | | 146645 | 4.35E-04 |
| 30905 | 30936 | P | 32 | 127096 | | 127127 | 4.35E-04 |
| 60119 | 60150 | F | 32 | 305406 | | 305437 | 4.35E-04 |
| 89949 | 89980 | F | 32 | 118554 | | 118585 | 4.35E-04 |
| 91235 | 91266 | P | 32 | 459387 | | 459418 | 4.35E-04 |
| 97812 | 97843 | F | 32 | 287642 | | 287673 | 4.35E-04 |
| 100420 | 100451 | P | 32 | 308979 | | 309010 | 4.35E-04 |
| 109558 | 109589 | P | 32 | 307048 | | 307079 | 4.35E-04 |
| 118554 | 118585 | F | 32 | 209319 | | 209350 | 4.35E-04 |
| 128688 | 128719 | P | 32 | 145861 | | 145892 | 4.35E-04 |
| 143761 | 143792 | F | 32 | 206218 | | 206249 | 4.35E-04 |
| 153008 | 153039 | F | 32 | 308979 | | 309010 | 4.35E-04 |
| 175869 | 175900 | P | 32 | 201810 | | 201841 | 4.35E-04 |
| 179815 | 179846 | F | 32 | 308979 | | 309010 | 4.35E-04 |
| 190170 | 190201 | P | 32 | 308979 | | 309010 | 4.35E-04 |
| 197287 | 197318 | P | 32 | 419855 | | 419886 | 4.35E-04 |
| 210387 | 210418 | P | 32 | 308979 | | 309010 | 4.35E-04 |
| 210444 | 210475 | P | 32 | 308979 | | 309010 | 4.35E-04 |
| 226146 | 226177 | F | 32 | 308979 | | 309010 | 4.35E-04 |
| 266486 | 266517 | P | 32 | 308979 | | 309010 | 4.35E-04 |
| 290578 | 290609 | P | 32 | 308979 | | 309010 | 4.35E-04 |
| 308979 | 309010 | P | 32 | 339796 | | 339827 | 4.35E-04 |
| 308979 | 309010 | P | 32 | 382733 | | 382764 | 4.35E-04 |
| 308979 | 309010 | F | 32 | 397152 | | 397183 | 4.35E-04 |
| 308979 | 309010 | P | 32 | 401869 | | 401900 | 4.35E-04 |
| 308979 | 309010 | F | 32 | 416190 | | 416221 | 4.35E-04 |
| 317633 | 317664 | F | 32 | 434304 | | 434335 | 4.35E-04 |
| 331064 | 331095 | F | 32 | 343422 | | 343453 | 4.35E-04 |
| 380109 | 380140 | F | 32 | 399488 | | 399519 | 4.35E-04 |
| 459384 | 459415 | C | 32 | 460894 | | 460925 | 4.35E-04 |
| 459390 | 459421 | C | 32 | 460898 | | 460929 | 4.35E-04 |
| 460888 | 460919 | F | 32 | 460894 | | 460925 | 4.35E-04 |
| 23755 | 23785 | P | 31 | 136224 | | 136254 | 1.58E-03 |
| 23906 | 23936 | P | 31 | 206334 | | 206364 | 1.58E-03 |
| 26799 | 26829 | F | 31 | 298026 | | 298056 | 1.58E-03 |
| 27180 | 27210 | F | 31 | 298307 | | 298337 | 1.58E-03 |
| 29460 | 29490 | F | 31 | 146003 | | 146033 | 1.58E-03 |
| 30967 | 30997 | P | 31 | 127035 | | 127065 | 1.58E-03 |
| 30996 | 31026 | P | 31 | 127006 | | 127036 | 1.58E-03 |
| 48744 | 48774 | P | 31 | 180409 | | 180439 | 1.58E-03 |
| 48752 | 48782 | F | 31 | 203116 | | 203146 | 1.58E-03 |
| 48752 | 48782 | P | 31 | 245255 | | 245285 | 1.58E-03 |
| 48752 | 48782 | F | 31 | 457646 | | 457676 | 1.58E-03 |
| 48752 | 48782 | F | 31 | 457675 | | 457705 | 1.58E-03 |
| 49056 | 49086 | P | 31 | 169447 | | 169477 | 1.58E-03 |
| 49302 | 49332 | P | 31 | 262804 | | 262834 | 1.58E-03 |
| 49585 | 49615 | P | 31 | 280468 | | 280498 | 1.58E-03 |
| 54516 | 54546 | P | 31 | 100457 | | 100487 | 1.58E-03 |
| 54516 | 54546 | P | 31 | 100485 | | 100515 | 1.58E-03 |
| 54516 | 54546 | P | 31 | 100513 | | 100543 | 1.58E-03 |
| 54516 | 54546 | P | 31 | 100541 | | 100571 | 1.58E-03 |
| 54516 | 54546 | P | 31 | 100569 | | 100599 | 1.58E-03 |
| 54516 | 54546 | P | 31 | 100597 | | 100627 | 1.58E-03 |
| 59351 | 59381 | F | 31 | 59480 | | 59510 | 1.58E-03 |
| 71626 | 71656 | F | 31 | 275539 | | 275569 | 1.58E-03 |
| 77109 | 77139 | F | 31 | 143923 | | 143953 | 1.58E-03 |
| 77810 | 77840 | P | 31 | 416355 | | 416385 | 1.58E-03 |
| 91236 | 91266 | R | 31 | 460887 | | 460917 | 1.58E-03 |
| 91236 | 91266 | F | 31 | 460901 | | 460931 | 1.58E-03 |
| 93964 | 93994 | F | 31 | 252558 | | 252588 | 1.58E-03 |
| 97498 | 97528 | P | 31 | 405645 | | 405675 | 1.58E-03 |
| 99461 | 99491 | P | 31 | 144190 | | 144220 | 1.58E-03 |
| 100450 | 100480 | F | 31 | 180409 | | 180439 | 1.58E-03 |
| 100478 | 100508 | F | 31 | 180409 | | 180439 | 1.58E-03 |
| 100506 | 100536 | F | 31 | 180409 | | 180439 | 1.58E-03 |
| 100534 | 100564 | F | 31 | 180409 | | 180439 | 1.58E-03 |
| 100562 | 100592 | F | 31 | 180409 | | 180439 | 1.58E-03 |
| 100590 | 100620 | F | 31 | 180409 | | 180439 | 1.58E-03 |
| 100618 | 100648 | F | 31 | 180409 | | 180439 | 1.58E-03 |
| 124157 | 124187 | P | 31 | 226138 | | 226168 | 1.58E-03 |
| 124157 | 124187 | F | 31 | 266495 | | 266525 | 1.58E-03 |
| 133008 | 133038 | F | 31 | 380051 | | 380081 | 1.58E-03 |
| 143894 | 143924 | P | 31 | 331065 | | 331095 | 1.58E-03 |
| 166050 | 166080 | F | 31 | 280468 | | 280498 | 1.58E-03 |
| 179786 | 179816 | P | 31 | 180409 | | 180439 | 1.58E-03 |
| 180409 | 180439 | P | 31 | 308631 | | 308661 | 1.58E-03 |
| 180409 | 180439 | F | 31 | 339826 | | 339856 | 1.58E-03 |
| 180409 | 180439 | F | 31 | 432993 | | 433023 | 1.58E-03 |
| 180833 | 180863 | P | 31 | 405645 | | 405675 | 1.58E-03 |
| 185930 | 185960 | P | 31 | 291332 | | 291362 | 1.58E-03 |
| 199717 | 199747 | P | 31 | 205155 | | 205185 | 1.58E-03 |
| 210409 | 210439 | P | 31 | 457704 | | 457734 | 1.58E-03 |
| 210466 | 210496 | P | 31 | 457704 | | 457734 | 1.58E-03 |
| 252633 | 252663 | P | 31 | 425132 | | 425162 | 1.58E-03 |
| 258742 | 258772 | F | 31 | 266493 | | 266523 | 1.58E-03 |
| 258757 | 258787 | P | 31 | 457704 | | 457734 | 1.58E-03 |
| 262804 | 262834 | F | 31 | 339512 | | 339542 | 1.58E-03 |
| 275067 | 275097 | P | 31 | 330358 | | 330388 | 1.58E-03 |
| 276811 | 276841 | F | 31 | 412611 | | 412641 | 1.58E-03 |
| 291842 | 291872 | F | 31 | 419862 | | 419892 | 1.58E-03 |
| 300282 | 300312 | F | 31 | 407270 | | 407300 | 1.58E-03 |
| 303700 | 303730 | F | 31 | 438581 | | 438611 | 1.58E-03 |
| 339686 | 339716 | P | 31 | 400865 | | 400895 | 1.58E-03 |
| 343416 | 343446 | P | 31 | 424415 | | 424445 | 1.58E-03 |
| 382755 | 382785 | P | 31 | 457704 | | 457734 | 1.58E-03 |
| 459387 | 459417 | F | 31 | 460886 | | 460916 | 1.58E-03 |
| 459387 | 459417 | P | 31 | 460901 | | 460931 | 1.58E-03 |
| 459390 | 459420 | R | 31 | 460883 | | 460913 | 1.58E-03 |
| 460889 | 460919 | F | 31 | 460897 | | 460927 | 1.58E-03 |
| 460890 | 460920 | C | 31 | 460897 | | 460927 | 1.58E-03 |
| 10348 | 10377 | P | 30 | 27600 | | 27629 | 5.70E-03 |
| 10402 | 10431 | P | 30 | 426137 | | 426166 | 5.70E-03 |
| 10480 | 10509 | F | 30 | 97501 | | 97530 | 5.70E-03 |
| 10480 | 10509 | F | 30 | 180836 | | 180865 | 5.70E-03 |
| 14129 | 14158 | F | 30 | 432988 | | 433017 | 5.70E-03 |
| 24466 | 24495 | F | 30 | 296054 | | 296083 | 5.70E-03 |
| 26453 | 26482 | F | 30 | 297710 | | 297739 | 5.70E-03 |
| 30221 | 30250 | P | 30 | 127775 | | 127804 | 5.70E-03 |
| 30626 | 30655 | P | 30 | 127369 | | 127398 | 5.70E-03 |
| 30986 | 31015 | P | 30 | 127017 | | 127046 | 5.70E-03 |
| 31047 | 31076 | P | 30 | 126956 | | 126985 | 5.70E-03 |
| 37371 | 37400 | F | 30 | 254714 | | 254743 | 5.70E-03 |
| 43423 | 43452 | F | 30 | 43462 | | 43491 | 5.70E-03 |
| 48753 | 48782 | F | 30 | 138494 | | 138523 | 5.70E-03 |
| 58306 | 58335 | F | 30 | 102310 | | 102339 | 5.70E-03 |
| 58999 | 59028 | P | 30 | 181913 | | 181942 | 5.70E-03 |
| 60162 | 60191 | P | 30 | 102314 | | 102343 | 5.70E-03 |
| 83653 | 83682 | F | 30 | 181761 | | 181790 | 5.70E-03 |
| 85882 | 85911 | F | 30 | 139498 | | 139527 | 5.70E-03 |
| 85882 | 85911 | P | 30 | 210410 | | 210439 | 5.70E-03 |
| 85882 | 85911 | P | 30 | 210467 | | 210496 | 5.70E-03 |
| 85882 | 85911 | P | 30 | 258758 | | 258787 | 5.70E-03 |
| 85882 | 85911 | F | 30 | 308639 | | 308668 | 5.70E-03 |
| 85882 | 85911 | P | 30 | 382756 | | 382785 | 5.70E-03 |
| 87383 | 87412 | P | 30 | 102310 | | 102339 | 5.70E-03 |
| 89879 | 89908 | F | 30 | 223134 | | 223163 | 5.70E-03 |
| 89952 | 89981 | P | 30 | 397098 | | 397127 | 5.70E-03 |
| 91236 | 91265 | R | 30 | 459379 | | 459408 | 5.70E-03 |
| 91236 | 91265 | P | 30 | 460889 | | 460918 | 5.70E-03 |
| 91236 | 91265 | C | 30 | 460898 | | 460927 | 5.70E-03 |
| 97471 | 97500 | F | 30 | 287343 | | 287372 | 5.70E-03 |
| 98725 | 98754 | P | 30 | 449038 | | 449067 | 5.70E-03 |
| 98995 | 99024 | F | 30 | 288462 | | 288491 | 5.70E-03 |
| 105801 | 105830 | F | 30 | 307646 | | 307675 | 5.70E-03 |
| 108550 | 108579 | F | 30 | 222445 | | 222474 | 5.70E-03 |
| 117397 | 117426 | F | 30 | 223189 | | 223218 | 5.70E-03 |
| 124157 | 124186 | F | 30 | 190179 | | 190208 | 5.70E-03 |
| 124157 | 124186 | P | 30 | 397145 | | 397174 | 5.70E-03 |
| 127936 | 127965 | P | 30 | 146596 | | 146625 | 5.70E-03 |
| 129079 | 129108 | P | 30 | 145505 | | 145534 | 5.70E-03 |
| 138494 | 138523 | F | 30 | 203117 | | 203146 | 5.70E-03 |
| 138494 | 138523 | P | 30 | 245255 | | 245284 | 5.70E-03 |
| 138494 | 138523 | F | 30 | 457647 | | 457676 | 5.70E-03 |
| 138494 | 138523 | F | 30 | 457676 | | 457705 | 5.70E-03 |
| 139498 | 139527 | F | 30 | 457704 | | 457733 | 5.70E-03 |
| 162600 | 162629 | P | 30 | 313009 | | 313038 | 5.70E-03 |
| 180409 | 180438 | F | 30 | 290608 | | 290637 | 5.70E-03 |
| 184450 | 184479 | P | 30 | 305576 | | 305605 | 5.70E-03 |
| 188020 | 188049 | P | 30 | 433357 | | 433386 | 5.70E-03 |
| 197501 | 197530 | P | 30 | 355824 | | 355853 | 5.70E-03 |
| 203117 | 203146 | P | 30 | 409370 | | 409399 | 5.70E-03 |
| 206383 | 206412 | P | 30 | 246207 | | 246236 | 5.70E-03 |
| 209322 | 209351 | P | 30 | 397098 | | 397127 | 5.70E-03 |
| 211680 | 211709 | F | 30 | 432065 | | 432094 | 5.70E-03 |
| 219145 | 219174 | P | 30 | 290575 | | 290604 | 5.70E-03 |
| 219145 | 219174 | P | 30 | 339793 | | 339822 | 5.70E-03 |
| 219145 | 219174 | F | 30 | 416195 | | 416224 | 5.70E-03 |
| 226139 | 226168 | P | 30 | 258744 | | 258773 | 5.70E-03 |
| 252908 | 252937 | F | 30 | 447164 | | 447193 | 5.70E-03 |
| 258744 | 258773 | P | 30 | 397145 | | 397174 | 5.70E-03 |
| 262805 | 262834 | P | 30 | 274111 | | 274140 | 5.70E-03 |
| 308639 | 308668 | F | 30 | 457704 | | 457733 | 5.70E-03 |
| 317993 | 318022 | F | 30 | 333082 | | 333111 | 5.70E-03 |
| 336341 | 336370 | P | 30 | 357623 | | 357652 | 5.70E-03 |
| 338374 | 338403 | P | 30 | 435462 | | 435491 | 5.70E-03 |
| 409370 | 409399 | F | 30 | 432985 | | 433014 | 5.70E-03 |
| 416195 | 416224 | P | 30 | 424213 | | 424242 | 5.70E-03 |
| 425419 | 425448 | P | 30 | 435754 | | 435783 | 5.70E-03 |
| 459388 | 459417 | P | 30 | 460887 | | 460916 | 5.70E-03 |
| 459388 | 459417 | C | 30 | 460894 | | 460923 | 5.70E-03 |
| 459389 | 459418 | F | 30 | 460886 | | 460915 | 5.70E-03 |
| 459390 | 459419 | R | 30 | 460892 | | 460921 | 5.70E-03 |
| 459390 | 459419 | P | 30 | 460895 | | 460924 | 5.70E-03 |
| 460888 | 460917 | F | 30 | 460898 | | 460927 | 5.70E-03 |

| **Table S4: Total number of Simple Sequence Repeats (SSRs) identified within the chloroplast genome of *J. spinosa.*** | | | | | | | | | | | | | | | | |
| --- | --- | --- | --- | --- | --- | --- | --- | --- | --- | --- | --- | --- | --- | --- | --- | --- |
| **Repeats** | **3** | **4** | **5** | **6** | **7** | **8** | **9** | **10** | **11** | **12** | **13** | **14** | **15** | **16** | **total** |  |
| A | - | - | - | - | - | - | - | 12 | 10 | 3 | 2 |  |  |  | 27 |  |
| C | - | - | - | - | - | - | - |  |  |  |  |  |  | 1 | 1 |  |
| G | - | - | - | - | - | - | - | 1 |  |  |  |  |  |  | 1 |  |
| T | - | - | - | - | - | - | - | 13 | 7 | 4 | 4 |  | 1 |  | 29 |  |
| AT | - | - | 5 | 1 | 1 | 1 |  |  |  |  |  |  |  |  | 8 |  |
| TA | - | - | 2 | 2 | 1 |  |  | 1 |  |  |  |  |  |  | 6 |  |
| TC | - | - | 1 |  |  |  |  |  |  |  |  |  |  |  | 1 |  |
| AAT | - |  | 1 |  |  |  |  |  |  |  |  |  |  |  | 1 |  |
| ATA | - | 3 |  |  |  |  |  |  |  |  |  |  |  |  | 3 |  |
| TAA | - | 1 |  |  |  |  |  |  |  |  |  |  |  |  | 1 |  |
| AAAT | 2 |  |  |  |  |  |  |  |  |  |  |  |  |  | 2 |  |
| AATA | 2 |  |  |  |  |  |  |  |  |  |  |  |  |  | 2 |  |
| ATTA | 1 | 1 |  |  |  |  |  |  |  |  |  |  |  |  | 2 |  |
| TCTT | 1 |  |  |  |  |  |  |  |  |  |  |  |  |  | 1 |  |
| TGAA | 1 |  |  |  |  |  |  |  |  |  |  |  |  |  | 1 |  |
| TTAT | 3 |  |  |  |  |  |  |  |  |  |  |  |  |  | 3 |  |
| TTTC | 2 |  |  |  |  |  |  |  |  |  |  |  |  |  | 2 |  |
| AATTT | 1 |  |  |  |  |  |  |  |  |  |  |  |  |  | 1 |  |
| CAAAA | 1 |  |  |  |  |  |  |  |  |  |  |  |  |  | 1 |  |
| TATTC | 1 |  |  |  |  |  |  |  |  |  |  |  |  |  | 1 |  |
| TTTTA | 1 |  |  |  |  |  |  |  |  |  |  |  |  |  | 1 |  |
| TCAGAA | 1 |  |  |  |  |  |  |  |  |  |  |  |  |  | 1 |  |

| **Table S5 Pattern of Tandem Repeat distribution in the chloroplast genome of *J. spinosa.*** | | | | | | |
| --- | --- | --- | --- | --- | --- | --- |
| **No.** | **Size** | **Copy Number** | **Percent Matches** | **Start** | **End** | **Repeat Sequence** |
| 1 | 13 | 2.1 | 100 | 624 | 650 | AACTTTAGTATTA |
| 2 | 17 | 1.9 | 88 | 1096 | 1129 | TAAATAAAAAATAACTAC |
| 3 | 15 | 2.1 | 100 | 2863 | 2893 | TTCATCATATATGAA |
| 4 | 13 | 3.5 | 75 | 9804 | 9845 | ATAGAATATTAAA |
| 5 | 22 | 2.2 | 96 | 9802 | 9850 | TTATAGAATATTAAAATAGAAT |
| 6 | 13 | 5.5 | 74 | 11432 | 11505 | TACTTATGTTATA |
| 7 | 15 | 3.3 | 79 | 11535 | 11589 | ATTAAGTAAAATGAA |
| 8 | 15 | 2 | 100 | 14641 | 14670 | TTCTATTTCTATTCT |
| 9 | 2 | 37.5 | 73 | 14685 | 14756 | TA |
| 10 | 7 | 4.9 | 85 | 14754 | 14785 | TATTTAT |
| 11 | 12 | 4.2 | 76 | 14762 | 14813 | ATTTATTATTTA |
| 12 | 26 | 1.9 | 95 | 14825 | 14874 | TTTCTAGTACCTATTTCTAAACAGGG |
| 13 | 14 | 3.2 | 100 | 18531 | 18575 | TTACTTTCTATTCG |
| 14 | 10 | 3.6 | 92 | 29741 | 29775 | TTCAATCTAT |
| 15 | 23 | 2 | 86 | 31417 | 31462 | ATCTATCAAAATAAAGATACATA |
| 16 | 15 | 2 | 100 | 31506 | 31535 | CTTTATACATTAAAA |
| 17 | 20 | 2.1 | 86 | 33175 | 33213 | AAAATAAATAAGCAAATAA |
| 18 | 18 | 3.2 | 80 | 34643 | 34706 | ATAAGTTAGAGAGTAATT |
| 19 | 11 | 3.2 | 87 | 39474 | 39507 | TATATAATATA |
| 20 | 23 | 2.4 | 81 | 39479 | 39530 | AATATATAATTCTAATTATTTAG |
| 21 | 23 | 2 | 91 | 39496 | 39541 | TTAGAATATATAATTCTAATTAT |
| 22 | 23 | 2.7 | 82 | 39548 | 39607 | AATTAGAATTATAAAATTTTTAT |
| 23 | 17 | 2 | 100 | 39773 | 39806 | TATTGGTATATTATATT |
| 24 | 15 | 1.9 | 100 | 43810 | 43838 | GCTGTTGCACCAGGG |
| 25 | 22 | 2 | 90 | 48596 | 48638 | TTTGTAATAGATCTACGGGGTG |
| 26 | 12 | 3.6 | 79 | 52679 | 52729 | CTTTTTCTTTCATATT |
| 27 | 19 | 5.4 | 71 | 55138 | 55237 | TTCTATTTTTAATATTATT |
| 28 | 38 | 3.2 | 76 | 55119 | 55239 | TTCTATTTTTTATTTATATTTCTATTTTTATATATTAT |
| 29 | 13 | 2 | 100 | 59265 | 59290 | ATTAGTTGATAAT |
| 30 | 11 | 2.7 | 95 | 61210 | 61240 | TTATTAGTTTT |
| 31 | 13 | 2 | 100 | 61242 | 61267 | TACGAGATTTTAC |
| 32 | 14 | 1.9 | 100 | 61462 | 61488 | ATTAGTTAATAATA |
| 33 | 17 | 2.2 | 90 | 72274 | 72310 | AAATTACTAATAAAGCG |
| 34 | 21 | 1.9 | 94 | 73010 | 73048 | TAAATCCAAGCGACTCTTTCC |
| 35 | 15 | 2.1 | 93 | 75676 | 75706 | TAGCTAAGAGAAAAT |
| 36 | 22 | 3.2 | 78 | 85921 | 85987 | TATATTAATATATAATATATTA |
| 37 | 7 | 6.6 | 79 | 85939 | 85984 | ATTATAT |
| 38 | 8 | 9.5 | 73 | 85911 | 85987 | AATATATT |
| 39 | 21 | 3.8 | 84 | 94349 | 94431 | TTTGTCCAAGTCACTTCTTCTT |
| 40 | 21 | 3.2 | 87 | 94371 | 94438 | TTTTTGTCCAAGTTACTTCTT |
| 41 | 18 | 5.2 | 90 | 96797 | 96886 | TATTGATGATAGTGACGA |
| 42 | 9 | 8.6 | 69 | 96809 | 96885 | CGATAGTGA |
| 43 | 13 | 2 | 100 | 99454 | 99479 | TGTGATTCCTGTT |
| 44 | 18 | 2.1 | 95 | 105091 | 105128 | TTTTCTATTATATTAGTA |
| 45 | 32 | 2.1 | 97 | 113163 | 113228 | CATTGTTCAACTCTTTGACAACACGAAAAAAC |
| 46 | 16 | 2.1 | 100 | 119941 | 119973 | TAAACTACAAAAACTT |
| 47 | 32 | 2.1 | 97 | 136970 | 137035 | TGGTTTTTTCATGTTGTCAAAGAGTTGAACAA |
| 48 | 18 | 2.1 | 95 | 145070 | 145107 | AATACTAATAGAATAGAA |
| 49 | 13 | 2 | 100 | 150719 | 150744 | AACAGGAATCACA |
| 50 | 18 | 5.2 | 90 | 153312 | 153401 | ATATCGTCACTATCATCA |
| 51 | 9 | 8.6 | 69 | 153312 | 153388 | ATATCATCA |
| 52 | 21 | 4.1 | 84 | 155761 | 155849 | AAAAAGAGAAGTAACTTAGAC |

| **Table S6 Scattered repeats in the chloroplast genome of *J. spinosa.*** | | | | | | |
| --- | --- | --- | --- | --- | --- | --- |
| **Start site of the first part** | **End site of the first part** | **Type** | **Alignment length** | **The repeat length of the second part** | **The starting site of the second part** | **E-value** |
| 7563 | 7593 | C | 31 | 39632 | 39662 | 1.89E-04 |
| 14692 | 14722 | C | 31 | 39610 | 39640 | 1.89E-04 |
| 14729 | 14760 | C | 32 | 72914 | 72945 | 5.23E-05 |
| 18530 | 18560 | F | 31 | 18544 | 18574 | 1.56E-09 |
| 96805 | 96835 | F | 31 | 96859 | 96889 | 6.53E-06 |
| 153307 | 153337 | F | 31 | 153361 | 153391 | 6.53E-06 |
| 24670 | 24700 | F | 31 | 87015 | 87045 | 1.89E-04 |
| 94370 | 94401 | F | 32 | 94391 | 94422 | 1.74E-06 |
| 155774 | 155805 | F | 32 | 155795 | 155826 | 1.74E-06 |
| 113162 | 113195 | F | 34 | 113194 | 113227 | 2.49E-09 |
| 136969 | 137002 | F | 34 | 137001 | 137034 | 2.49E-09 |
| 153337 | 153370 | F | 34 | 153355 | 153388 | 2.49E-09 |
| 9176 | 9209 | F | 34 | 38565 | 38598 | 3.94E-06 |
| 96820 | 96853 | F | 34 | 96856 | 96889 | 3.94E-06 |
| 47244 | 47282 | F | 39 | 104172 | 104210 | 2.79E-12 |
| 42184 | 42224 | F | 41 | 44414 | 44454 | 4.29E-10 |
| 96808 | 96849 | F | 42 | 96844 | 96885 | 1.15E-10 |
| 153311 | 153352 | F | 42 | 153347 | 153388 | 1.15E-10 |
| 96820 | 96867 | F | 48 | 96838 | 96885 | 4.24E-14 |
| 153311 | 153358 | F | 48 | 153329 | 153376 | 4.24E-14 |
| 96808 | 96859 | F | 52 | 96826 | 96877 | 2.12E-16 |
| 153319 | 153370 | F | 52 | 153337 | 153388 | 2.12E-16 |
| 14727 | 14757 | R | 31 | 72914 | 72944 | 6.53E-06 |
| 96805 | 96835 | P | 31 | 153307 | 153337 | 6.53E-06 |
| 96859 | 96889 | P | 31 | 153361 | 153391 | 6.53E-06 |
| 94370 | 94401 | P | 32 | 155774 | 155805 | 1.74E-06 |
| 94391 | 94422 | P | 32 | 155795 | 155826 | 1.74E-06 |
| 1120 | 1151 | P | 32 | 1167 | 1198 | 5.23E-05 |
| 113162 | 113195 | P | 34 | 136969 | 137002 | 2.49E-09 |
| 113194 | 113227 | P | 34 | 137001 | 137034 | 2.49E-09 |
| 29124 | 29157 | P | 34 | 29124 | 29157 | 1.23E-07 |
| 96820 | 96853 | P | 34 | 153307 | 153340 | 3.94E-06 |
| 96856 | 96889 | P | 34 | 153343 | 153376 | 3.94E-06 |
| 74957 | 74992 | P | 36 | 74957 | 74992 | 1.52E-12 |
| 47244 | 47282 | P | 39 | 145986 | 146024 | 2.79E-12 |
| 65005 | 65045 | P | 41 | 65005 | 65045 | 4.29E-10 |
| 96808 | 96849 | P | 42 | 153311 | 153352 | 1.15E-10 |
| 96844 | 96885 | P | 42 | 153347 | 153388 | 1.15E-10 |
| 79426 | 79469 | P | 44 | 79426 | 79469 | 2.33E-17 |
| 96820 | 96867 | P | 48 | 153311 | 153358 | 4.24E-14 |
| 96838 | 96885 | P | 48 | 153329 | 153376 | 4.24E-14 |
| 96808 | 96859 | P | 52 | 153319 | 153370 | 2.12E-16 |
| 96826 | 96877 | P | 52 | 153337 | 153388 | 2.12E-16 |
| 0 | 612 | P | 613 | 89584 | 90196 | 0.00E+00 |
| 90197 | 115784 | P | 25588 | 134412 | 159999 | 0.00E+00 |

| **Table S7: Codon usage for *J. spinosa* mitochondrion genome. Total: 11738 codons, using 40 CDS** | | | | | | | | | |
| --- | --- | --- | --- | --- | --- | --- | --- | --- | --- |
| **Amino Acid** | **Codon** | **Number** |  | **RSCU** | **Amino Acid** | **Codon** | **Number** | **RSCU** |  |
| Phe | UUU | 428 | 765 | 1.12 | Ala | GCU | 298 | 1.59 | 750 |
|  | UUC | 337 |  | 0.88 |  | GCC | 175 | 0.93 |  |
| Leu | UUA | 297 | 1261 | 1.41 |  | GCA | 184 | 0.98 |  |
|  | UUG | 250 |  | 1.19 |  | GCG | 93 | 0.5 |  |
|  | CUU | 286 |  | 1.36 | His | CAU | 219 | 1.53 | 286 |
|  | CUC | 129 |  | 0.61 |  | CAC | 67 | 0.47 |  |
|  | CUA | 186 |  | 0.89 | Gln | CAA | 248 | 1.51 | 329 |
|  | CUG | 113 |  | 0.54 |  | CAG | 81 | 0.49 |  |
| Ile | AUU | 382 | 861 | 1.33 | Asn | AAU | 245 | 1.31 | 373 |
|  | AUC | 247 |  | 0.86 |  | AAC | 128 | 0.69 |  |
|  | AUA | 232 |  | 0.81 | Lys | AAA | 306 | 1.15 | 532 |
| Met | AUG | 300 | 300 | 1 |  | AAG | 226 | 0.85 |  |
| Val | GUU | 211 | 714 | 1.18 | Asp | GAU | 255 | 1.37 | 373 |
|  | GUC | 142 |  | 0.8 |  | GAC | 118 | 0.63 |  |
|  | GUA | 202 |  | 1.13 | Glu | GAA | 335 | 1.37 | 488 |
|  | GUG | 159 |  | 0.89 |  | GAG | 153 | 0.63 |  |
| Ser | UCU | 257 | 1080 | 1.43 | Cys | UGU | 103 | 1.21 | 170 |
|  | UCC | 167 |  | 0.93 |  | UGC | 67 | 0.79 |  |
|  | UCA | 200 |  | 1.11 | Trp | UGG | 186 | 1 | 186 |
|  | UCG | 160 |  | 0.89 | Arg | CGU | 174 | 1.25 | 838 |
|  | AGU | 183 |  | 1.02 |  | CGC | 81 | 0.58 |  |
|  | AGC | 113 |  | 0.63 |  | CGA | 187 | 1.34 |  |
| Pro | CCU | 243 | 656 | 1.48 |  | CGG | 94 | 0.67 |  |
|  | CCC | 120 |  | 0.73 |  | AGA | 200 | 1.43 |  |
|  | CCA | 180 |  | 1.1 |  | AGG | 102 | 0.73 |  |
|  | CCG | 113 |  | 0.69 | Gly | GGU | 253 | 1.27 | 794 |
| Thr | ACU | 201 | 592 | 1.36 |  | GGC | 108 | 0.54 |  |
|  | ACC | 157 |  | 1.06 |  | GGA | 293 | 1.48 |  |
|  | ACA | 144 |  | 0.97 |  | GGG | 140 | 0.71 |  |
|  | ACG | 90 |  | 0.61 | TER | UAA | 18 | 1.42 | 38 |
| Tyr | UAU | 265 | 352 | 1.51 |  | UGA | 12 | 0.95 |  |
|  | UAC | 87 |  | 0.49 |  | UAG | 8 | 0.63 |  |

| **Table S8 Codon usage for *J. spinosa* chloroplast genome. Total: 26192 codons, using 84 CDS.** | | | | | | | | | |
| --- | --- | --- | --- | --- | --- | --- | --- | --- | --- |
| **Amino Acid** | **Codon** | **Number** |  | **RSCU** | **Amino Acid** | **Codon** | **Number** | **RSCU** |  |
| Phe | UUU | 959 | 1492 | 1.29 | Ala | GCU | 621 | 1.78 | 1394 |
|  | UUC | 533 |  | 0.71 |  | GCC | 226 | 0.65 |  |
| Leu | UUA | 862 | 2756 | 1.88 |  | GCA | 387 | 1.11 |  |
|  | UUG | 565 |  | 1.23 |  | GCG | 160 | 0.46 |  |
|  | CUU | 595 |  | 1.3 | His | CAU | 479 | 1.52 | 629 |
|  | CUC | 176 |  | 0.38 |  | CAC | 150 | 0.48 |  |
|  | CUA | 379 |  | 0.83 | Gln | CAA | 715 | 1.53 | 935 |
|  | CUG | 179 |  | 0.39 |  | CAG | 220 | 0.47 |  |
| Ile | AUU | 1099 | 2254 | 1.46 | Asn | AAU | 994 | 1.55 | 1284 |
|  | AUC | 458 |  | 0.61 |  | AAC | 290 | 0.45 |  |
|  | AUA | 697 |  | 0.93 | Lys | AAA | 1017 | 1.46 | 1389 |
| Met | AUG | 601 | 601 | 1 |  | AAG | 372 | 0.54 |  |
| Val | GUU | 515 | 1414 | 1.46 | Asp | GAU | 878 | 1.61 | 1091 |
|  | GUC | 176 |  | 0.5 |  | GAC | 213 | 0.39 |  |
|  | GUA | 515 |  | 1.46 | Glu | GAA | 1028 | 1.5 | 1367 |
|  | GUG | 208 |  | 0.59 |  | GAG | 339 | 0.5 |  |
| Ser | UCU | 566 | 2035 | 1.67 | Cys | UGU | 231 | 1.53 | 302 |
|  | UCC | 328 |  | 0.97 |  | UGC | 71 | 0.47 |  |
|  | UCA | 420 |  | 1.24 | Trp | UGG | 458 | 1 | 458 |
|  | UCG | 184 |  | 0.54 | Arg | CGU | 353 | 1.35 | 1573 |
|  | AGU | 420 |  | 1.24 |  | CGC | 101 | 0.39 |  |
|  | AGC | 117 |  | 0.34 |  | CGA | 357 | 1.36 |  |
| Pro | CCU | 429 | 1072 | 1.6 |  | CGG | 112 | 0.67 |  |
|  | CCC | 194 |  | 0.72 |  | AGA | 485 | 0.43 |  |
|  | CCA | 302 |  | 1.13 |  | AGG | 165 | 0.63 |  |
|  | CCG | 147 |  | 0.55 | Gly | GGU | 576 | 1.31 | 1765 |
| Thr | ACU | 528 | 1331 | 1.59 |  | GGC | 165 | 0.37 |  |
|  | ACC | 251 |  | 0.75 |  | GGA | 718 | 1.63 |  |
|  | ACA | 407 |  | 1.22 |  | GGG | 306 | 0.69 |  |
|  | ACG | 145 |  | 0.44 | TER | UAA | 42 | 1.5 | 84 |
| Tyr | UAU | 775 | 966 | 1.6 |  | UGA | 25 | 0.89 |  |
|  | UAC | 191 |  | 0.4 |  | UAG | 17 | 0.61 |  |

| **Table S9 RNA editing sites in mitochondrial coding regions of *J. spinosa.*** | | | | | |
| --- | --- | --- | --- | --- | --- |
| **Gene name** | **Editing position in Genome** | **Edited Type** | **Codon position** | **Codon Change** | **Amino Acid Change** |
| *atp1* | 102822 | C>T | 3 | ACC->ACT | T->T |
| *atp1* | 103072 | C>T | 1 | CGA->TGA | R->* |
| *atp1* | 103615 | C>T | 1 | CCC->TCC | P->S |
| *atp1* | 103640 | C>T | 2 | TCG->TTG | S->L |
| *atp1* | 103754 | C>T | 2 | TCA->TTA | S->L |
| *atp1* | 103792 | C>T | 1 | CTT->TTT | L->F |
| *atp1* | 103868 | C>T | 2 | CCG->CTG | P->L |
| *atp1* | 103991 | C>T | 2 | CCA->CTA | P->L |
| *atp1* | 104066 | C>T | 2 | CCA->CTA | P->L |
| *atp4* | 177916 | C>T | 2 | CCG->CTG | P->L |
| *atp4* | 177919 | C>T | 2 | CCT->CTT | P->L |
| *atp4* | 177940 | C>T | 2 | CCC->CTC | P->L |
| *atp4* | 177952 | C>T | 2 | TCG->TTG | S->L |
| *atp4* | 178078 | C>T | 2 | TCA->TTA | S->L |
| *atp6* | 448557 | C>T | 1 | CAA->TAA | Q->* |
| *atp6* | 448568 | C>T | 2 | ACA->ATA | T->I |
| *atp6* | 448604 | C>T | 2 | TCT->TTT | S->F |
| *atp6* | 448611 | C>T | 1 | CAT->TAT | H->Y |
| *atp6* | 448619 | C>T | 2 | TCA->TTA | S->L |
| *atp6* | 448640 | C>T | 2 | CCG->CTG | P->L |
| *atp6* | 448676 | C>T | 2 | CCT->CTT | P->L |
| *atp6* | 448727 | C>T | 2 | TCC->TTC | S->F |
| *atp6* | 449005 | C>T | 3 | CTC->CTT | L->L |
| *atp6* | 449013 | C>T | 1 | CGT->TGT | R->C |
| *atp6* | 449021 | C>T | 2 | TCG->TTG | S->L |
| *atp6* | 449039 | C>T | 2 | TCG->TTG | S->L |
| *atp6* | 449046 | C>T | 1 | CGC->TGC | R->C |
| *atp6* | 449102 | C>T | 2 | CCG->CTG | P->L |
| *atp6* | 449108 | C>T | 2 | CCG->CTG | P->L |
| *atp6* | 449159 | C>T | 2 | TCG->TTG | S->L |
| *atp6* | 449238 | C>T | 1 | CCA->TCA | P->S |
| *atp6* | 449255 | C>T | 2 | TCT->TTT | S->F |
| *atp8* | 184388 | C>T | 2 | TCA->TTA | S->L |
| *atp8* | 184399 | C>T | 1 | CTC->TTC | L->F |
| *atp8* | 184417 | C>T | 1 | CCC->TCC | P->S |
| *atp8* | 184418 | C>T | 2 | CCC->CTC | P->L |
| *atp8* | 184419 | C>T | 3 | CCC->CCT | P->P |
| *atp8* | 184793 | C>T | 2 | CCA->CTA | P->L |
| *atp8* | 184811 | C>T | 2 | TCA->TTA | S->L |
| *atp9* | 410538 | C>T | 2 | TCA->TTA | S->L |
| *atp9* | 410652 | C>T | 2 | TCA->TTA | S->L |
| *atp9* | 410709 | C>T | 2 | CCA->CTA | P->L |
| *atp9* | 410730 | C>T | 2 | CCA->CTA | P->L |
| *atp9* | 410733 | C>T | 2 | TCC->TTC | S->F |
| *atp9* | 410741 | C>T | 1 | CGA->TGA | R->* |
| *cob* | 193824 | C>T | 1 | CCT->TCT | P->S |
| *cob* | 193827 | C>T | 1 | CCT->TCT | P->S |
| *cob* | 193926 | C>T | 1 | CAC->TAC | H->Y |
| *cob* | 194000 | C>T | 2 | CCA->CTA | P->L |
| *cob* | 194055 | C>T | 1 | CAT->TAT | H->Y |
| *cob* | 194100 | C>T | 1 | CCC->TCC | P->S |
| *cob* | 194171 | C>T | 2 | TCT->TTT | S->F |
| *cob* | 194340 | C>T | 1 | CAT->TAT | H->Y |
| *cob* | 194583 | C>T | 1 | CAT->TAT | H->Y |
| *cob* | 194620 | C>T | 3 | CTC->CTT | L->L |
| *cob* | 194622 | C>T | 1 | CTC->TTC | L->F |
| *cob* | 194790 | C>T | 1 | CCG->TCG | P->S |
| *cox1* | 355726 | C>T | 2 | CCG->CTG | P->L |
| *cox1* | 355792 | C>T | 2 | TCA->TTA | S->L |
| *cox1* | 355820 | C>T | 1 | CGT->TGT | R->C |
| *cox1* | 355823 | C>T | 1 | CGT->TGT | R->C |
| *cox1* | 355946 | C>T | 1 | CTC->TTC | L->F |
| *cox1* | 355951 | C>T | 2 | CCG->CTG | P->L |
| *cox1* | 356039 | C>T | 1 | CAC->TAC | H->Y |
| *cox1* | 356146 | C>T | 2 | CCG->CTG | P->L |
| *cox1* | 356510 | C>T | 1 | CGG->TGG | R->W |
| *cox1* | 356557 | C>T | 2 | TCT->TTT | S->F |
| *cox1* | 356635 | C>T | 2 | CCA->CTA | P->L |
| *cox1* | 356674 | C>T | 2 | TCA->TTA | S->L |
| *cox1* | 356710 | C>T | 2 | TCC->TTC | S->F |
| *cox1* | 356773 | C>T | 2 | TCT->TTT | S->F |
| *cox2* | 262216 | C>T | 2 | TCA->TTA | S->L |
| *cox2* | 262313 | C>T | 1 | CGG->TGG | R->W |
| *cox2* | 262414 | C>T | 2 | CCA->CTA | P->L |
| *cox2* | 262439 | C>T | 1 | CGG->TGG | R->W |
| *cox2* | 262529 | C>T | 1 | CGG->TGG | R->W |
| *cox2* | 262665 | C>T | 3 | CTC->CTT | L->L |
| *cox3* | 186430 | C>T | 2 | CCG->CTG | P->L |
| *cox3* | 186580 | C>T | 2 | CCT->CTT | P->L |
| *cox3* | 186589 | C>T | 2 | CCT->CTT | P->L |
| *matR* | 125676 | C>T | 2 | TCA->TTA | S->L |
| *matR* | 125694 | C>T | 2 | CCA->CTA | P->L |
| *matR* | 125764 | C>T | 1 | CAC->TAC | H->Y |
| *matR* | 125786 | C>T | 3 | TAC->TAT | Y->Y |
| *matR* | 125800 | C>T | 1 | CGC->TGC | R->C |
| *matR* | 125820 | C>T | 2 | CCT->CTT | P->L |
| *matR* | 125840 | C>T | 3 | TCC->TCT | S->S |
| *matR* | 125841 | C>T | 2 | TCC->TTC | S->F |
| *matR* | 126176 | C>T | 3 | ACC->ACT | T->T |
| *matR* | 127107 | C>T | 2 | TCG->TTG | S->L |
| *matR* | 127194 | C>T | 2 | CCA->CTA | P->L |
| *matR* | 127327 | C>T | 1 | CCA->TCA | P->S |
| *matR* | 127505 | C>T | 3 | ATC->ATT | I->I |
| *mttB* | 280738 | C>T | 1 | CGT->TGT | R->C |
| *mttB* | 280795 | C>T | 1 | CCG->TCG | P->S |
| *mttB* | 280857 | C>T | 2 | CCA->CTA | P->L |
| *mttB* | 280900 | C>T | 1 | CCA->TCA | P->S |
| *mttB* | 280933 | C>T | 1 | CAT->TAT | H->Y |
| *nad1* | 124888 | C>T | 1 | CGG->TGG | R->W |
| *nad1* | 124907 | C>T | 3 | TTC->TTT | F->F |
| *nad1* | 124918 | C>T | 1 | CGG->TGG | R->W |
| *nad1* | 125061 | C>T | 2 | CCG->CTG | P->L |
| *nad1* | 125073 | C>T | 2 | CCA->CTA | P->L |
| *nad1* | 125076 | C>T | 2 | TCT->TTT | S->F |
| *nad1* | 125082 | C>T | 2 | TCG->TTG | S->L |
| *nad1* | 125091 | C>T | 2 | CCA->CTA | P->L |
| *nad1* | 277406 | C>T | 1 | CCT->TCT | P->S |
| *nad2* | 4042 | C>T | 2 | TCA->TTA | S->L |
| *nad2* | 4083 | C>T | 3 | CCC->CCT | P->P |
| *nad2* | 4091 | C>T | 1 | CTA->TTA | L->L |
| *nad2* | 4096 | C>T | 2 | TCC->TTC | S->F |
| *nad2* | 4099 | C>T | 2 | TCA->TTA | S->L |
| *nad2* | 4201 | C>T | 2 | GCG->GTG | A->V |
| *nad2* | 5722 | C>T | 1 | CGT->TGT | R->C |
| *nad2* | 5752 | C>T | 1 | CCA->TCA | P->S |
| *nad2* | 5871 | C>T | 2 | TCG->TTG | S->L |
| *nad2* | 5940 | C>T | 2 | TCA->TTA | S->L |
| *nad2* | 6036 | C>T | 2 | ACT->ATT | T->I |
| *nad2* | 6070 | C>T | 1 | CAT->TAT | H->Y |
| *nad2* | 6078 | C>T | 2 | CCT->CTT | P->L |
| *nad2* | 6189 | C>T | 2 | TCT->TTT | S->F |
| *nad2* | 6210 | C>T | 2 | TCT->TTT | S->F |
| *nad3* | 168243 | C>T | 2 | TCA->TTA | S->L |
| *nad3* | 168282 | C>T | 2 | CCG->CTG | P->L |
| *nad3* | 168299 | C>T | 1 | CTA->TTA | L->L |
| *nad3* | 168317 | C>T | 1 | CCA->TCA | P->S |
| *nad3* | 168318 | C>T | 2 | CCA->CTA | P->L |
| *nad3* | 168384 | C>T | 2 | TCC->TTC | S->F |
| *nad3* | 168446 | C>T | 1 | CCT->TCT | P->S |
| *nad3* | 168447 | C>T | 2 | CCT->CTT | P->L |
| *nad3* | 168453 | C>T | 2 | CCG->CTG | P->L |
| *nad3* | 168504 | C>T | 2 | CCG->CTG | P->L |
| *nad3* | 168520 | C>T | 3 | TCC->TCT | S->S |
| *nad3* | 168582 | C>T | 2 | TCG->TTG | S->L |
| *nad3* | 168587 | C>T | 1 | CGG->TGG | R->W |
| *nad4* | 242148 | C>T | 2 | TCT->TTT | S->F |
| *nad4* | 242163 | C>T | 2 | CCT->CTT | P->L |
| *nad4* | 242193 | C>T | 2 | ACT->ATT | T->I |
| *nad4* | 242196 | C>T | 2 | CCT->CTT | P->L |
| *nad4* | 242436 | C>T | 2 | TCA->TTA | S->L |
| *nad4* | 242481 | C>T | 2 | ACA->ATA | T->I |
| *nad4* | 242487 | C>T | 2 | TCT->TTT | S->F |
| *nad4* | 242495 | C>T | 1 | CGT->TGT | R->C |
| *nad4* | 242522 | C>T | 1 | CGC->TGC | R->C |
| *nad4* | 242555 | C>T | 1 | CCC->TCC | P->S |
| *nad4* | 242556 | C>T | 2 | CCC->CTC | P->L |
| *nad4* | 242568 | C>T | 2 | CCA->CTA | P->L |
| *nad4* | 243013 | C>T | 1 | CCG->TCG | P->S |
| *nad4* | 244160 | C>T | 3 | ATC->ATT | I->I |
| *nad4* | 244211 | C>T | 3 | TTC->TTT | F->F |
| *nad4* | 244319 | C>T | 2 | TCC->TTC | S->F |
| *nad4* | 244388 | C>T | 2 | TCT->TTT | S->F |
| *nad4* | 244448 | C>T | 2 | TCA->TTA | S->L |
| *nad4L* | 178455 | C>T | 2 | CCA->CTA | P->L |
| *nad4L* | 178464 | C>T | 2 | TCA->TTA | S->L |
| *nad4L* | 178473 | C>T | 2 | TCA->TTA | S->L |
| *nad5* | 78408 | C>T | 2 | TCA->TTA | S->L |
| *nad5* | 78933 | C>T | 2 | ACG->ATG | T->M |
| *nad5* | 78945 | C>T | 2 | TCT->TTT | S->F |
| *nad5* | 78973 | C>T | 1 | CCA->TCA | P->S |
| *nad5* | 79044 | C>T | 2 | TCG->TTG | S->L |
| *nad5* | 79083 | C>T | 2 | TCA->TTA | S->L |
| *nad5* | 79095 | C>T | 2 | TCG->TTG | S->L |
| *nad5* | 79132 | C>T | 1 | CTT->TTT | L->F |
| *nad5* | 79179 | C>T | 2 | TCT->TTT | S->F |
| *nad5* | 79200 | C>T | 2 | GCC->GTC | A->V |
| *nad5* | 79210 | C>T | 1 | CGT->TGT | R->C |
| *nad5* | 79314 | C>T | 2 | ACA->ATA | T->I |
| *nad5* | 79410 | C>T | 2 | TCT->TTT | S->F |
| *nad5* | 79566 | C>T | 2 | CCG->CTG | P->L |
| *nad5* | 79587 | C>T | 3 | TTC->TTT | F->F |
| *nad5* | 80503 | C>T | 2 | CCG->CTG | P->L |
| *nad7* | 324639 | C>T | 1 | CGC->TGC | R->C |
| *nad7* | 324668 | C>T | 2 | CCT->CTT | P->L |
| *nad7* | 324669 | C>T | 1 | CCT->TCT | P->S |
| *nad7* | 324674 | C>T | 2 | TCG->TTG | S->L |
| *nad7* | 324710 | C>T | 2 | TCG->TTG | S->L |
| *nad7* | 325887 | C>T | 2 | TCA->TTA | S->L |
| *nad7* | 326082 | C>T | 2 | TCA->TTA | S->L |
| *nad7* | 326121 | C>T | 2 | TCA->TTA | S->L |
| *nad7* | 326130 | C>T | 2 | TCA->TTA | S->L |
| *nad7* | 326149 | C>T | 1 | CGT->TGT | R->C |
| *nad7* | 326214 | C>T | 2 | TCA->TTA | S->L |
| *nad7* | 326221 | C>T | 1 | CAT->TAT | H->Y |
| *nad7* | 327469 | C>T | 2 | TCA->TTA | S->L |
| *nad7* | 327478 | C>T | 2 | TCT->TTT | S->F |
| *nad7* | 328500 | C>T | 2 | TCA->TTA | S->L |
| *nad7* | 328506 | C>T | 2 | TCA->TTA | S->L |
| *nad7* | 328539 | C>T | 2 | TCC->TTC | S->F |
| *nad7* | 328545 | C>T | 2 | TCG->TTG | S->L |
| *nad9* | 155277 | C>T | 1 | CAT->TAT | H->Y |
| *nad9* | 155300 | C>T | 2 | TCG->TTG | S->L |
| *nad9* | 155375 | C>T | 2 | TCT->TTT | S->F |
| *nad9* | 155391 | G>A | 1 | GAA->AAA | E->K |
| *nad9* | 155453 | C>T | 2 | TCC->TTC | S->F |
| *rpl16* | 31419 | C>T | 2 | TCC->TCT | S->S |
| *rpl16* | 31433 | C>T | 2 | CCA->CTA | P->L |
| *rpl16* | 31499 | C>T | 2 | TCA->TTA | S->L |
| *rpl16* | 31505 | C>T | 2 | TCG->TTG | S->L |
| *rpl5* | 196599 | C>T | 2 | CCG->CTG | P->L |
| *rpl5* | 196602 | C>T | 2 | CCA->CTA | P->L |
| *rpl5* | 196785 | C>T | 2 | TCG->TTG | S->L |
| *rpl5* | 196797 | C>T | 2 | TCG->TTG | S->L |
| *rpl5* | 196945 | C>T | 1 | CGC->TGC | R->C |
| *rpl5* | 196948 | C>T | 1 | CCG->TCG | P->S |
| *rpl5* | 197022 | C>T | 2 | TCG->TTG | S->L |
| *rpl5* | 197055 | C>T | 2 | CCG->CTG | P->L |
| *rpl5* | 197067 | C>T | 2 | CCG->CTG | P->L |
| *rpl5* | 197079 | C>T | 2 | TCA->TTA | S->L |
| *rpl5* | 197107 | C>T | 1 | CCA->TCA | P->S |
| *rps1* | 400682 | C>T | 2 | TCC->TTC | S->F |
| *rps10* | 357388 | C>T | 2 | TCG->TTG | S->L |
| *rps10* | 358260 | C>T | 1 | CGG->TGG | R->W |
| *rps10* | 358482 | C>T | 1 | CGC->TGC | R->C |
| *rps10* | 358496 | C>T | 2 | ACG->ATG | T->M |
| *rps12* | 168714 | C>T | 2 | TCG->TTG | S->L |
| *rps12* | 168743 | C>T | 1 | CGC->TGC | R->C |
| *rps12* | 168747 | C>T | 2 | CCG->CTG | P->L |
| *rps12* | 168789 | C>T | 2 | CCA->CTA | P->L |
| *rps12* | 168839 | C>T | 1 | CAC->TAC | H->Y |
| *rps12* | 168864 | C>T | 2 | TCG->TTG | S->L |
| *rps12* | 168912 | C>T | 2 | TCG->TTG | S->L |
| *rps12* | 168927 | C>T | 2 | TCC->TTC | S->F |
| *rps13* | 278391 | C>T | 3 | TCG->TTG | S->L |
| *rps14* | 196284 | C>T | 1 | CCT->TCT | P->S |
| *rps14* | 196361 | C>T | 2 | TCC->TTC | S->F |
| *rps14* | 196372 | C>T | 3 | AAC->AAT | N->N |
| *rps19* | 121374 | C>T | 2 | TCT->TTT | S->F |
| *rps19* | 121431 | C>T | 2 | CCT->CTT | P->L |
| *rps19* | 121432 | C>T | 1 | CCT->TCT | P->S |
| *rps19* | 121457 | C>T | 3 | TCC->TCT | S->S |
| *rps3* | 31024 | C>T | 1 | CCT->TCT | P->S |
| *sdh3* | 40291 | C>T | 1 | CCA->TCA | P->S |
| *sdh3* | 40298 | C>T | 2 | TCA->TTA | S->L |
| *sdh3* | 40428 | C>T | 3 | ATC->ATT | I->I |
| *sdh4* | 186931 | C>T | 3 | CCC->CCT | P->P |
| *sdh4* | 187047 | C>T | 2 | CCA->CTA | P->L |
| *sdh4* | 187095 | C>T | 2 | CCA->CTA | P->L |
| *sdh4* | 187151 | C>T | 1 | CAT->TAT | H->Y |
| *sdh4* | 187245 | C>T | 2 | TCT->TTT | S->F |

| **Table S10 RNA editing sites in chloroplast coding regions of *J. spinosa.*** | | | | | |
| --- | --- | --- | --- | --- | --- |
| **Gene name** | **Editing position in Genome** | **Edited Type** | **Codon position** | **Codon Change** | **Amino Acid Change** |
| *atpA* | 12393 | C->T | 1 | CCT -> TCT | P->S |
| *atpF* | 14479 | C->T | 2 | CCA -> CTA | P->L |
| *clpP* | 75191 | C->T | 3 | CGC -> CGT | R->R |
| *ndhA* | 125738 | C->T | 2 | TCC -> TTC | S->F |
| *ndhA* | 127512 | C->T | 2 | TCT -> TTT | S->F |
| *ndhB* | 148287 | C->T | 3 | TTC -> TTT | F->F |
| *ndhB* | 149863 | C->T | 2 | CCA -> CTA | P->L |
| *ndhC* | 54375 | C->T | 2 | TCA -> TTA | S->L |
| *ndhC* | 54522 | G->A | 2 | CGA -> CAA | R->Q |
| *ndhC* | 54481 | G->A | 1 | GAT -> AAT | D->N |
| *ndhC* | 54457 | C->T | 1 | CCG -> TCG | P->S |
| *ndhD* | 121715 | C->T | 2 | TCA -> TTA | S->L |
| *ndhD* | 121727 | C->T | 2 | TCA -> TTA | S->L |
| *ndhD* | 122138 | C->T | 2 | TCT -> TTT | S->F |
| *ndhD* | 122147 | C->T | 2 | TCT -> TTT | S->F |
| *ndhD* | 122351 | C->T | 2 | TCA -> TTA | S->L |
| *ndhD* | 122642 | C->T | 2 | TCA -> TTA | S->L |
| *ndhE* | 123725 | C->T | 2 | TCA -> TTA | S->L |
| *ndhF* | 117793 | C->T | 2 | TCA -> TTA | S->L |
| *ndhG* | 124673 | C->T | 2 | TCA -> TTA | S->L |
| *ndhK* | 54169 | G->A | 2 | AGT -> AAT | S->N |
| *ndhK* | 54194 | C->T | 1 | CTT -> TTT | L->F |
| *ndhK* | 54144 | C->T | 2 | ACC -> ATC | T->I |
| *ndhK* | 54220 | C->T | 2 | TCA -> TTA | S->L |
| *ndhK* | 53917 | C->T | 2 | ACC -> ATC | T->I |
| *ndhK* | 53975 | G->A | 1 | GAA -> AAA | E->K |
| *petB* | 80865 | C->T | 3 | GTC -> GTT | V->V |
| *petL* | 70725 | C->T | 2 | CTT -> CTT | L->L |
| *petL* | 70773 | C->T | 2 | ATC -> ATC | I->I |
| *psaB* | 40838 | C->T | 2 | TCG -> TTG | S->L |
| *psaI* | 63847 | C->T | 2 | TCT -> TTT | S->F |
| *psaI* | 63852 | C->T | 1 | CAT -> TAT | H->Y |
| *psbB* | 78007 | C->T | 3 | ATC -> ATT | I->I |
| *psbZ* | 39102 | G->A | 2 | TCA -> TTA | S->L |
| *rpl16* | 86976 | C->T | 1 | CCC -> TCC | P->S |
| *rpoA* | 84021 | C->T | 2 | TCT -> TTT | S->F |
| *rpoB* | 26456 | G->A | 1 | GCA -> ACA | A->T |
| *rpoB* | 26423 | G->A | 1 | GCA -> ACA | A->T |
| *rpoB* | 26663 | C->T | 1 | CGT -> TGT | R->C |
| *rpoB* | 28525 | C->T | 2 | TCA -> TTA | S->L |
| *rpoB* | 28510 | C->T | 2 | TCG -> TTG | S->L |
| *rpoB* | 25898 | G->A | 1 | GAG -> AAG | E->K |
| *rpoB* | 26691 | G->A | 2 | GGG -> GAG | G->E |
| *rpoB* | 26258 | G->A | 1 | GTT -> ATT | V->I |
| *rpoB* | 26486 | G->A | 2 | GGA -> GAA | G->E |
| *rpoC1* | 25765 | C->T | 3 | GCC -> GCT | A->A |
| *rps12* | 104026 | C->T | 2 | TCA -> TTA | S->L |
| *rps12* | 146170 | C->T | 2 | TCA -> TTA | S->L |
| *rps14* | 40614 | C->T | 2 | TCA -> TTA | S->L |
| *rps14* | 40545 | C->T | 2 | TCA -> TTA | S->L |
| *rps16* | 6186 | C->T | 2 | TCA -> TTA | S->L |
| *rps18* | 73224 | C->T | 2 | TCG -> TTG | S->L |
| *rps2* | 18273 | C->T | 2 | ACA -> ATA | T->I |
| *rps2* | 18159 | C->T | 2 | TCA -> TTA | S->L |
| *ycf1* | 134401 | C->T | 2 | TCT -> TTT | S->F |
| *psbF* | 68972 | C->T | 2 | TCT -> TTT | S->F |

| **Table S11 The homologous DNA fragmentsbetween mitochondrial genome and chloroplast genome of *J. spinosa* (Genes shown in red represent complete transfers.).** | | | | | | | | | |
| --- | --- | --- | --- | --- | --- | --- | --- | --- | --- |
| **Plastome** | **start** | **end** | **Pt annotation** | **% identity** | **Alignmentlength** | **Mitogenome** | **start** | **end** | **Mt annotation** |
| Pt | 88852 | 101497 | *rpl22,rps19,rpl2,rpl23,trnH_GUG,trnI-CAU,trnM_CAU,ycf2,trnL_UAG,trnL-CAA,ndhB* | 98.627 | 12675 | Mt | 231484 | 244124 | *trnI-CAU,trnL-CAA* |
| Pt | 147473 | 159990 | *ndhB,trnL-CAA,trnL_UAG,ycf2,trnI-CAU,trnM_CAU,trnH_GUG,rpl23,rpl2* | 98.725 | 12547 | Mt | 244124 | 231602 | *trnI-CAU,trnL-CAA* |
| Pt | 133848 | 139476 | *ycf1,trnN_GUU,trnR_ACG,rrn5,trnA_UGC, rrn4,trnA_UGC* | 99.858 | 5631 | Mt | 292873 | 298502 | *trnN-GUU,trnR-ACG* |
| Pt | 109494 | 115066 | *rrn23,rrn4.5,rrn5,trnR-ACG,trnN-GUU,ycf1* | 99.874 | 5575 | Mt | 298502 | 292929 | *trnN-GUU,trnR-ACG* |
| Pt | 80553 | 86101 | *petB,petD,rpoA,rps11,rpl36,rps8,rpl14* | 96.182 | 5553 | Mt | 40511 | 35056 | *IGS*(*rrn26,atp9*) |
| Pt | 39573 | 43868 | *trnG-GCC,trnM-CAU,rps14,psaB,psaA* | 98.557 | 4298 | Mt | 360433 | 364716 | *IGS*(*atp1,nad6*) |
| Pt | 140820 | 144459 | *trnA-UGC,trnI-GAU,rrn16,trnV-GAC* | 98.257 | 3671 | Mt | 289796 | 286141 | *trnV-GAC,trnI-GAU* |
| Pt | 104511 | 108150 | *trnV-GAC,rrn16,trnI-GAU* | 98.257 | 3671 | Mt | 286141 | 289796 | *trnV-GAC,trnI-GAU* |
| Pt | 43847 | 47302 | *ycf3* | 97.557 | 3479 | Mt | 77763 | 81223 | *IGS*(*ccmFn,trnQ-UUG*) |
| Pt | 21820 | 22901 | *rpoC2* | 99.261 | 1082 | Mt | 1082 | 1 | *IGS*(*trnY-GUA,rps7*) |
| Pt | 87695 | 88862 | *rps3,rpl22* | 93.691 | 1173 | Mt | 276978 | 278137 | *IGS*(*atp8,nad4L*) |
| Pt | 22902 | 23645 | *rpoC1* | 99.462 | 744 | Mt | 461675 | 460932 | *IGS*(*trnY-GUA,rps7*) |
| Pt | 70393 | 71082 | *petL,petG* | 83.807 | 704 | Mt | 306462 | 305810 | *trnP-UGG,trnW-CCA* |
| Pt | 107470 | 107834 | *rrn16* | 96.438 | 365 | Mt | 373902 | 373548 | *IGS*(*atp1,nad6*) |
| Pt | 141136 | 141500 | *trnI-GAU* | 96.438 | 365 | Mt | 373548 | 373902 | *IGS*(*atp1,nad6*) |
| Pt | 144474 | 144804 | *IGS*(*trnV-GAC,rps7*) | 97.281 | 331 | Mt | 286155 | 285825 | *IGS*(*atp4,trnV-GAC*) |
| Pt | 104166 | 104496 | *IGS*(*rps7,trnV-GAC*) | 97.281 | 331 | Mt | 285825 | 286155 | *IGS*(*atp4,trnV-GAC*) |
| Pt | 142219 | 143082 | *rrn16* | 74.132 | 893 | Mt | 98579 | 97719 | *IGS*(*rrn18,rrn5*) |
| Pt | 105888 | 106751 | *rrn16* | 74.132 | 893 | Mt | 97719 | 98579 | *IGS*(*rrn18,rrn5*) |
| Pt | 66424 | 66590 | *cemA* | 97.041 | 169 | Mt | 34887 | 35055 | *IGS*(*rrn26,atp9*) |
| Pt | 48185 | 48480 | *IGS*(*ycf3,trnS-GGA*) | 80.333 | 300 | Mt | 3907 | 3625 | *trnS-GGA* |
| Pt | 32166 | 32285 | *IGS*(*psbM,trnD-GUC*) | 90.833 | 120 | Mt | 221289 | 221170 | *trnD-GUC* |
| Pt | 113827 | 113910 | *IGS*(*trnN-GUU,trnN-GUU*) | 97.619 | 84 | Mt | 449599 | 449516 | *trnN-GUU* |
| Pt | 135060 | 135143 | *ycf1* | 97.619 | 84 | Mt | 449516 | 449599 | *trnN-GUU* |
| Pt | 56 | 142 | *IGS*(*rpl23,rpl2*) | 96.552 | 87 | Mt | 231467 | 231382 | *trnH-GUG* |
| Pt | 55847 | 55925 | *trnV-UAC* | 94.937 | 79 | Mt | 92810 | 92732 | *trnM-CAU* |
| Pt | 133743 | 133798 | *ycf1* | 98.214 | 56 | Mt | 292873 | 292928 | *IGS*(*nad3,trnN-GUU*) |
| Pt | 8570 | 8660 | *psbK* | 79.787 | 94 | Mt | 3810 | 3899 | *IGS*(*rps7,trnS-GGA*) |
| Pt | 90987 | 91061 | *rpl2* | 81.609 | 87 | Mt | 344401 | 344315 | *ccmC* |
| Pt | 157909 | 157983 | *ycf2* | 81.609 | 87 | Mt | 344315 | 344401 | *ccmC* |
| Pt | 33816 | 33852 | *IGS*(*trnE-UUC,trnT_UGU*) | 94.595 | 37 | Mt | 220989 | 220953 | *IGS*(*nad4,trnD-GUC*) |
| Pt | 31676 | 31704 | *psbM* | 100 | 29 | Mt | 238188 | 238216 | *IGS*(*trnI-CAU,trnL-CAA*) |
| Pt | 143066 | 143094 | *rrn16* | 100 | 29 | Mt | 314971 | 314943 | *IGS*(*trnW-CCA,ccmFc*) |
| Pt | 105876 | 105904 | *IGS*(*rps7,trnV-GAC*) | 100 | 29 | Mt | 314943 | 314971 | *IGS*(*trnW-CCA,ccmFc*) |

| **Table S12 GenBank accession numbers of 11 chloroplast genomes of plant species used in this study.** | | | | |  |
| --- | --- | --- | --- | --- | --- |
| **Name** | **Family** | **Genus** | **GenBank accessions** | **Size (bp)** |  |
| *Arabidopsis thaliana* | Brassicaceae | *Arabidopsis* | NC_000932 | 154478 |  |
| *Brassica napus* | Brassicaceae | *Brassica* | NC_016734 | 152860 |  |
| *Vasconcellea carvalhoae* | Caricaceae | *Vasconcellea* | NC_065369 | 158723 |  |
| *Carica papaya* | Caricaceae | *Carica* | NC_010323 | 160100 |  |
| *Vasconcellea cundinamarcensis* | Caricaceae | *Vasconcellea* | NC_049867 | 158712 |  |
| *Capparis spinosa* | Capparaceae | *Capparis* | MT041701 | 157728 |  |
| *Aethionema arabicum* | Brassicaceae | *Aethionema* | NC_034367 | 154234 |  |
| *Tarenaya hassleriana* | Cleomaceae | *Tarenaya* | NC_034364 | 157688 |  |
| *Gynandropsis gynandra* | Cleomaceae | *Gynandropsis* | NC_054276 | 158152 |  |
| *Bretschneidera sinensis* | Akaniaceae | *Bretschneidera* | OR756295 | 159043 |  |
| *Liquidambar acalycina* | Hamamelidaceae | *Liquidambar* | MN879312 | 160434 | Outgroups |

| **Table S13 Number of structural variations compared with *C. papaya*** | | | | |
| --- | --- | --- | --- | --- |
|  | *ycf1* | | | |
| Genus name | length | INS | DEL | SNP |
| *J. spinosa* | 5568 | 2 | 4 | 130 |
| *V. cundinamarcensis* | 5565 | 2 | 4 | 179 |
| *V. carvalhoae* | 5565 | 2 | 4 | 175 |
|  | *rps19* | | | |
|  | length | INS | DEL | SNP |
| *J. spinosa* | 278 | 0 | 0 | 4 |
| *V. cundinamarcensis* | 278 | 0 | 0 | 4 |
| *V. carvalhoae* | 278 | 0 | 0 | 4 |
|  | *rpoC2* | | | |
|  | length | INS | DEL | SNP |
| *J. spinosa* | 4184 | 1 | 0 | 30 |
| *V. cundinamarcensis* | 2423 | 2 | 0 | 31 |
| *V. carvalhoae* | 2423 | 2 | 0 | 31 |

| **Table S14 Gene composition in this mitochondrial genome of *C. papaya.*** | | |
| --- | --- | --- |
| **Group of genes** | **Name of genes** | **Number of genes** |
| Subunit of ATPase | *atp1, atp4, atp6, atp8, atp9(×2)* | 6 |
| Cytochrome c biogenesis | *ccmB, ccmC, ccmFC*, ccmFN(×2)* | 5 |
| Apocytochrome b | *cob* | 1 |
| Subunit of cytochrome c oxidase | *cox1, cox2*, cox3* | 3 |
| Maturase R | *matR* | 1 |
| Transport membrane protein | *mttB* | 1 |
| Subunit of NADH dehydrogenase | *nad1****, nad2****, nad3, nad4***, nad4L, nad5*, nad6, nad7****, nad9* | 9 |
| Small subunit of ribosome | *rps1, rps10*, rps12, rps13, rps14, rps19, rps3*, rps4, rps7* | 9 |
| Large subunit of ribosome | *rpl10, rpl16, rpl2*, rpl5* | 4 |
| Subunit of succinate dehydrogenase | *sdh3, sdh4* | 2 |
| tRNA genes | *trnC-GCA,trnD-GUC,trnE-UUC,trnF-GAA,trnfM-CAU（×2）,trnG-GCC,trnH-GUG,trnI-CAU（×2）,trnK-UUU,trnL-CAA,trnM-CAU,trnN-GUU,trnP-UGG（×2）,trnQ-UUG,trnS-GCU,trnS-GGA,trnS-UGA,trnW-CCA,trnY-GUA* | 22 |
| rRNA genes | *rrn18, rrn26, rrn5* | 3 |

Notes:*:intron number; Gene (×2) : Number of copies of multi-copy genes.

| **Table S15 Top ten self-BLAST hits of the *C. papaya* mitochondrial genome.** | | | | | | | | | | |
| --- | --- | --- | --- | --- | --- | --- | --- | --- | --- | --- |
| Query ID | Subject ID | % identity | | Alignment length | Mismatches | Query start | Query end | Subject start | Subject end | E-value |
| mt | mt | | 100 | 476890 | 0 | 1 | 476890 | 1 | 476890 | 0 |
| mt | mt | | 100 | 11105 | 0 | 465786 | 476890 | 218701 | 229805 | 0 |
| mt | mt | | 100 | 11105 | 0 | 218701 | 229805 | 465786 | 476890 | 0 |
| mt | mt | | 99.825 | 571 | 1 | 421628 | 422198 | 152874 | 152304 | 0 |
| mt | mt | | 99.825 | 571 | 1 | 152304 | 152874 | 422198 | 421628 | 0 |
| mt | mt | | 83.686 | 662 | 77 | 167088 | 167733 | 150851 | 150205 | 1.05E-168 |
| mt | mt | | 83.71 | 663 | 75 | 150205 | 150851 | 167733 | 167088 | 1.05E-168 |
| mt | mt | | 98.706 | 309 | 4 | 440916 | 441224 | 401551 | 401243 | 8.28E-155 |
| mt | mt | | 100 | 297 | 0 | 401255 | 401551 | 441212 | 440916 | 8.28E-155 |
| mt | mt | | 94.393 | 214 | 10 | 229601 | 229812 | 114160 | 113947 | 4.22E-88 |
